# Assessing the Effectiveness of Policies Relating to Breastfeeding Promotion, Protection, and Support in Southeast Asia: Protocol for a Mixed Methods Study

**DOI:** 10.2196/21286

**Published:** 2020-09-21

**Authors:** Tuan T Nguyen, Amy Weissman, Jennifer Cashin, Tran T Ha, Paul Zambrano, Roger Mathisen

**Affiliations:** 1 Alive & Thrive FHI 360 Hanoi Vietnam; 2 Asia Pacific Regional Office FHI 360 Bangkok Thailand; 3 Alive & Thrive FHI 360 Yangon Myanmar; 4 Research and Training Center for Community Development (RTCCD) Hanoi Vietnam; 5 Alive & Thrive FHI 360 Manila Philippines

**Keywords:** breastfeeding, breast milk substitute, Code of Marketing of Breast-milk Substitutes (The Code), maternity protection, maternal, infant, and young child nutrition, mixed methods study, Southeast Asia

## Abstract

**Background:**

Despite its well-known benefits, breastfeeding practices remain suboptimal worldwide, including in Southeast Asia. Many countries in the region have thus enacted policies, such as maternity protection and the World Health Assembly International Code of Marketing of Breast-milk Substitutes (the Code), that protect, promote, and support breastfeeding. Yet the impact of such national legislation on breastfeeding practices is not well understood.

**Objective:**

This study aims to review the content, implementation, and potential impact of policies relating to maternity protection and the Code in Myanmar, the Philippines, Thailand, and Vietnam.

**Methods:**

This mixed methods study includes a desk review, trend and secondary data analyses, and quantitative and qualitative data collection. Desk reviews will examine and compare the contents, implementation strategies, coverage, monitoring, and enforcement of national policies focusing on maternity protection and the Code in each country with global standards. Trend and secondary data analyses will examine the potential impact of these policies on relevant variables such as breast milk substitute (BMS) sales and women’s workforce participation. Quantitative data collection and analysis will be conducted to examine relevant stakeholders’ and beneficiaries’ perceptions about these policies. In each country, we will conduct up to 24 in-depth interviews (IDI) with stakeholders at national and provincial levels and 12 employers or 12 health workers. Per country, we will survey approximately 930 women who are pregnant or have a child aged 0-11 months, of whom approximately 36 will be invited for an IDI; 12 partners of the interviewed mothers or fathers of children from 0-11 months will also be interviewed.

**Results:**

This study, funded in June 2018, was approved by the Institutional Review Boards of the relevant organizations (FHI 360: April 16, 2019 and May 18, 2020; and Hanoi University of Public Health: December 6, 2019). The dates of data collection are as follows: Vietnam: November and December 2019, May and June 2020; the Philippines: projected August 2020; Myanmar and Thailand: pending based on permissions and funding. Results are expected to be published in January 2021. As of July 2020, we had enrolled 1150 participants. We will present a comparison of key contents of the policies across countries and against international standards and recommendations and a comparison of implementation strategies, coverage, monitoring, and enforcement across countries. We will also present findings from secondary data and trend data analyses to propose the potential impact of a new or amended policy. For the surveys with women, we will present associations between exposure to maternity protection or BMS promotion on infant and young child feeding practices and their determinants. Findings from IDIs will highlight relevant stakeholders’ and beneficiaries’ perceptions.

**Conclusions:**

This study will increase the understanding of the effectiveness of policy interventions to improve breastfeeding, which will be used to advocate for stronger policy adoption and enforcement in study countries and beyond.

**International Registered Report Identifier (IRRID):**

DERR1-10.2196/21286

## Introduction

### Breastfeeding Benefits and Determinants

The benefits of breastfeeding for child survival, health, and development as well as maternal health and wellbeing have been well-documented in low-income, middle-income, and high-income countries [1]. By improving breastfeeding rates, an estimated 595,379 deaths due to diarrhea and pneumonia among children 6-59 months of age and 98,243 maternal deaths due to breast and ovarian cancer and type 2 diabetes, along with 974,956 cases of childhood obesity, could be prevented annually [2]. In addition to being better protected against infectious diseases and overweight and obesity, children who have been breastfed are at reduced risk for type 2 diabetes [3], perform better on intelligence tests [3], have higher educational attainment [4], and have higher adult earnings [5]. Women who have breastfed are more likely to maintain a healthy body weight [6,7] and less likely to suffer from depression [8]. Given the benefits of breastfeeding, the World Health Organization (WHO) and the United Nations Children’s Fund (UNICEF) recommend that mothers start breastfeeding their babies within the first hour of life and exclusively for the first 6 months and continue to breastfeed along with appropriate complementary feeding up to 2 years of age and beyond [9]. Breastfeeding generates significant economic gains for households, communities, and countries. Analysis by the World Bank suggests that every dollar invested in breastfeeding generates US $35 in economic returns [10]. Globally, the total economic losses due to health system treatment costs, premature mortality, and cognitive losses are estimated to be US $341.3 billion per year or about 0.7% of global gross national income [2].

Despite all that is known about the benefits of breastfeeding, breastfeeding practices remain suboptimal worldwide [1,11]. Although nearly all women are biologically capable of breastfeeding, the decision to breastfeed is influenced by a variety of societal, community, household, and individual factors [11]. According to Rollins et al [11], breastfeeding is determined by factors at structural or societal levels in different settings like the health system, workplace, and community and at the individual level. Within the health care system, providers strongly influence feeding decisions during the period before, during, and after birth and are the most likely sources of support when challenges occur [12,13]. The extent to which health care providers are equipped to deal with breastfeeding challenges can impact breastfeeding practices [1,11]. Family and community are also important sources of breastfeeding support at the interpersonal level, and women whose partners support breastfeeding breastfeed longer [14,15]. On an individual level, a mother’s decision to breastfeed is influenced by the advice and support she receives, her confidence and self-efficacy to breastfeed, as well as positioning and attachment [16-19].

Breastfeeding is also influenced at the structural level, such as by policies that can support, protect, or promote the practice, including maternity protection and the international code on the marketing of breast milk substitutes (BMS).

### Maternity Protection

Across the globe, a mother’s return to work after childbirth is one of the primary reasons for not breastfeeding or for early cessation of breastfeeding [20]. Increasing the duration of paid maternity leave has been shown to be an effective intervention for improving breastfeeding rates. Recent analysis of trend data from 38 low-income and middle-income countries illustrates that increasing the duration of paid maternity leave is associated with a significantly higher prevalence of early initiation of breastfeeding, exclusive breastfeeding among infants <6 months old, and longer duration of breastfeeding [21]. Mothers can continue breastfeeding after returning to work if maternity leave or childcare is available and if breastfeeding or the expressing of breast milk is supported [22]. Multiple studies have shown that providing working mothers with time, space, and support for breastfeeding when they return to work can increase breastfeeding duration and adherence to recommended breastfeeding practices [23-25].

The International Labor Organization (ILO) Maternity Protection Convention, 2000 (No. 183) [26] and its accompanying Recommendation (No. 191) [27] call for the establishment of an integrated set of essential measures to help initiate, establish, and maintain optimal breastfeeding practices: (1) maternity leave for at least 14 weeks with full pay; (2) prenatal, childbirth, and postnatal health care for both the mother and her child and cash benefits for women who do not qualify for social insurance; (3) protection for a pregnant or nursing worker from engaging in work that could be detrimental to her health or that of her child; (4) the right to return to the same or similar paid position at the same salary rate; (5) protection from discrimination at work; and (6) the right to one or more daily breaks or a reduction in working time. Compliance and commitment to the ILO Convention vary significantly by country [26,28], with nearly 50% of countries not meeting the minimum 14 weeks (Conventions 183) and less than 25% meeting the convention No. 191 of 18 weeks or more. The sources of funding for maternity entitlements also vary from public financing, employers, employee contributions, and a mix of these [28]. Some policies provide support to women after returning to work, while others do not [28]. To effectively advocate for appropriate maternity protection policies, it is necessary to study the impact of existing policies as well as perceptions of maternity protection among employees, employers, and policy makers.

### World Health Assembly International Code of Marketing of Breast-Milk Substitutes

Breastfeeding is also affected by the aggressive marketing and widespread availability of BMS. Such marketing can influence social norms in favor of BMS and undermine mothers’ confidence to breastfeed, resulting in suboptimal breastfeeding practices [29]. To limit inappropriate marketing practices and the harmful effects of marketing of BMS, feeding bottles, and teats, the World Health Assembly (WHA) adopted the International Code of Marketing of Breast-milk Substitutes (referred to as the Code) [30] and subsequent WHA resolutions.

As of April 2020, 136 of 194 (70%) WHO member states have implemented legal measures related to the Code. Of these, only 25 countries have measures “substantially aligned” with the Code based on a WHO/UNICEF/ International Baby Food Action Network checklist of provisions of the Code and relevant WHA resolutions [31]. Further, although for legal measures to be effective they must include clear provisions enabling authorized agencies to take corrective action when Code violations are identified, only 73 (73/194, 38%) countries clearly identify which government agencies are responsible for monitoring compliance, and only 82 (82/194, 42%) countries define sanctions for violations [31].

Although there is evidence of the negative impact of the provision of free BMS samples or promotion through trusted health workers [32,33], there has been little exploration of how national policies on the Code have impacted breastfeeding. Similarly, no studies have directly evaluated the impact of the Code on marketing and promotion practices, exposure to advertising, attitudes towards BMS and breastfeeding, and BMS sales [29].

### Study Goal and Objectives

The goal of this study is to determine the impact of breastfeeding-related policies in Myanmar, the Philippines, Thailand, and Vietnam. To do so, researchers will review the content, implementation, and potential impact of policies relating to breastfeeding promotion, protection, and support in these countries; however, depending on the local context, the study in each country will examine key policies related to maternity protection and the Code.

Specific study objectives are to (1) review the content of national policies on maternity protection and the Code; (2) review the implementation, coverage, monitoring, and enforcement of these policies across countries; (3) examine the potential impact of these policies on relevant outcomes (eg, for maternity protection, impact on workforce participation by women; for the Code, impact on exposure to BMS marketing); and (4) examine perceptions of relevant stakeholders and beneficiaries (eg, pregnant and lactating women) about these policies (eg, perceived benefits, limitations, difficulties, areas for improvement, recommendations).

## Methods

### Study Setting

The study will be conducted in Myanmar, the Philippines, Thailand, and Vietnam. In recent years, each of these countries has made a significant policy change related to maternity protection or the Code.

#### Myanmar

Working mothers in Myanmar are entitled to 14 weeks of paid maternity leave (18 weeks for twins) in the private sector under the Social Security Law (2012) and the Leave and Holidays Act (1951, amended 2014) [34]. Mothers working in the public sector are entitled to 6 months paid maternity leave according to the revised Handbook of Civil Servant Rights (2013) [34]. Employers are required to pay a woman’s wages while she is on maternity leave, regardless of whether she is enrolled in the social security system. Women who are enrolled in the social security system are entitled to additional cash and noncash benefits during pregnancy and after delivery. According to the Factories Act (1951, amended 2016), in factories where more than 100 female workers are employed, employers must set up and maintain a childcare room [34]. There is no legislation that addresses nursing breaks at work.

In 2014, Myanmar’s Food and Drug Board of Authority issued the Order of Marketing of Formulated Food for Infant and Young Child under the National Food Law, which regulates the marketing and labeling of BMS, complementary foods, and bottles and teats intended for children under 2 years of age.

#### The Philippines

In the Philippines, working mothers are entitled to 9 weeks of paid maternity leave, all of which is funded by social security [28]. Since 2018, the Republic Act has provided more supportive policies, including increasing the maternity leave period to 105 days for female workers with an option to extend for an additional 30 days without pay and granting an additional 15 days for solo mothers. This substantially increased the previous entitlement of 9 weeks and covers public, private, and informal sector workers. The Expanded Breastfeeding Promotion Act (2009) of the Philippines provides that expenses for establishing lactation stations are tax-deductible. The Department of Labor and Employment issued a policy to guide the establishment of lactation stations in varied workplace settings and have included these spaces in the monitoring of labor law compliance of private enterprises.

The Philippines was among the first countries to pass national legislation on the Code in 1986. In 2006, the Implementing Rules and Regulations of the Code were revised to align with international standards and were approved [35]. Executive Order 51 covers infant formula, follow-up formula, feeding bottles and teats, and other related products, but not complementary foods or milk for mothers. While monitoring of the Code and its Implementing Rules and Regulations resulted in the processing of 24 alleged violations between 2011 and 2012 [36], monitoring and enforcement at national and subnational levels remain a challenge. In 2018, a new **Republic Act** revised the definition of BMS by law to include any type of milk marketed for feeding of infants aged up to 36 months.

#### Thailand

Thailand has not yet ratified ILO Convention 183, which provides maternity protection. In 1990, Thailand approved the Social Security Act B.E 2553 entitling mothers to 90 days of maternity leave. During maternity leave, 50% of the salary is paid by the employer for 45 days, and 50% is paid by social security for 90 days. To be eligible for social security payments, the insured person must have paid contributions for at least 7 months during the 15 months prior to the date of receiving medical benefits. Currently, there is no mention of breastfeeding breaks in the Thai law.

In 2017, the National Legislative Assembly passed the Marketing Control on Food for Infants and Young Children Act to restrict the marketing of food for infants and young children. Under the revised legislation, the government of Thailand prohibits all marketing of BMS for children under 12 months of age, with further regulations that apply to marketing of similar products to children under 3 years of age.

#### Vietnam

The National Assembly extended paid maternity leave from 4 to 6 months, effective from 2013. Working mothers in Vietnam are also entitled to paid nursing breaks (up to 60 minutes) by law, for up to 12 months [28]. The funding is from the social security fund.

In 2012, the National Assembly voted in favor of implementing a total ban on the promotion of BMS for children up to 2 years of age. The government guiding decree on marketing and use of feeding products for young children, feeding bottles, teats, and pacifiers was approved in 2014 [37].

### Study Design and Methods

**Figure 1:**
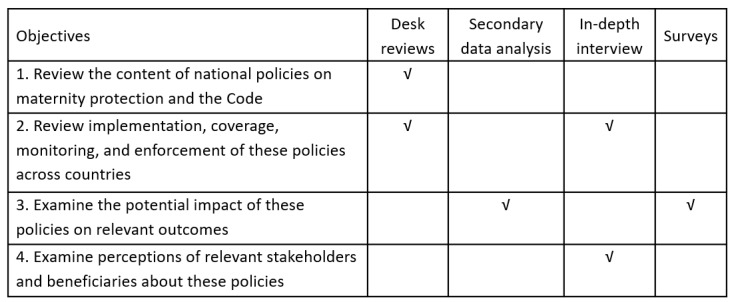
Methods for each study objective.

**Figure 2:**
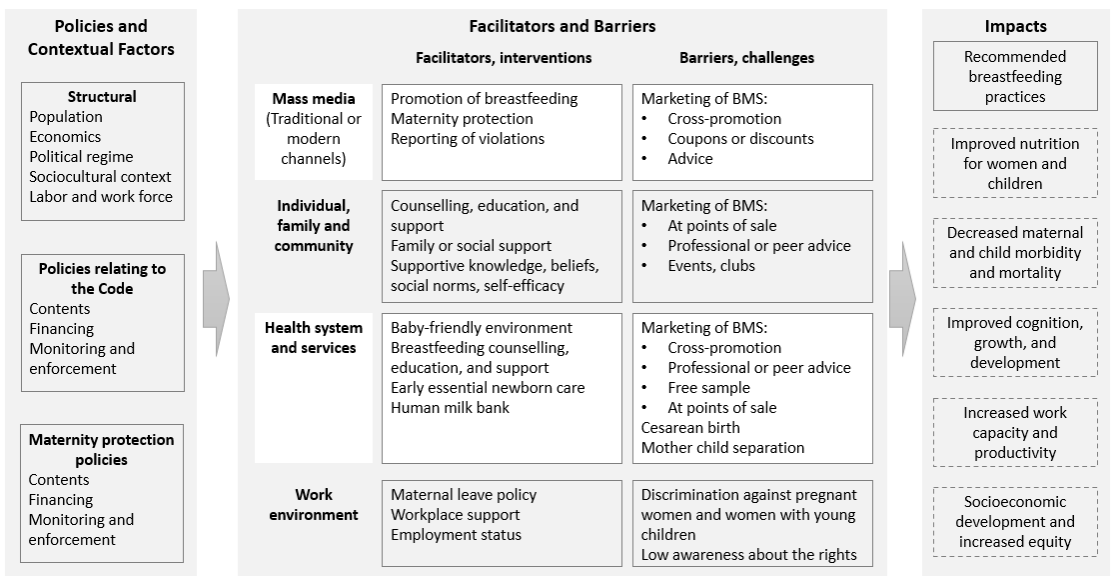
Conceptual model. The dashed lines indicate content not measured in the study. BMS: breast milk substitute.

This is a mixed-methods study, employing desk reviews, secondary data analysis, and primary quantitative and qualitative data collection (Figure 1). Our study was designed using a conceptual framework (Figure 2) that was developed based on previous literature [11,38-46].

#### Desk Review Policy Content and Implementation

For each country, we will collect and review relevant maternity protection and the Code policies. These will be collected through government websites (eg, Parliament, Ministry of Health) and other official sources. Key informants, consultants, or country staff will help to identify and collect documents not publicly available. Researchers will extract key information from the collected documents using a semiquantitative policy data extraction form (Multimedia Appendix 1). Two researchers will review each policy, compare the results, and discuss the differences before creating the final form. The extracted information will then be compared to international standards (eg, the Code and subsequent WHA resolutions and ILO Maternity Protection Convention 2000 [No. 183] [26] and Maternity Protection Recommendation 2000 [No. 191] [27]).

We will review the implementation, coverage, monitoring, and enforcement of relevant policies focusing on maternity protection and the Code. Key informants, consultants, or country staff will help to identify and collect documents and data related to the number and proportion of working mothers receiving maternity benefits, details of monitoring and reporting activities, as well as the number of the Code violations and penalties enforced.

#### Secondary Data Analysis

Secondary data will be drawn from national maternal, infant, and young child nutrition and health surveys, such as Multiple Indicator Cluster Surveys (MICS), Demographic and Health Surveys (DHS), or labor force surveys. Because the availability of secondary data sets and trends may vary across countries, we will identify all relevant data (eg, based on reviewing survey questionnaires, codebook, and reports) and secure access via locally contracted research firms. Univariate, bivariate, and multivariate data analysis will be used to analyze individual-level data.

We will analyze data using an interrupted time-series approach to quantify the change in the slope or level of selected outcomes before and after the implementation of a policy [47]. For maternity protection, the trend data could be the number of women participating in the workforce, recipients, and expenditures that might be accessed and used. For the Code, trend data will be advertising occurrence and expenditure of BMS companies by product and channel (obtained through media agencies or a rapid media audit) and sales of BMS by BMS type (purchased from Euromonitor [48]). The research team has successfully used interrupted time-series analysis in a prior publication [49].

#### Primary Qualitative and Quantitative Data Collection

We will conduct in-depth interviews (IDIs) with stakeholders, including policy makers or authorities at national and subnational levels; staff of the United Nations, nongovernmental organizations (NGOs), research organizations, and media; and employers and health workers (Table 1). Participants must meet the following eligibility criteria: be at least 18 years of age; willing to participate in the study; and have knowledge, a role, or a responsibility relating to the maternity protection policies or the Code.

**Table 1:** Study participants, research methods, and sample size per country.

Participant group		In-depth interview, n (per country)	Quantitative survey, n (per country)
**National level**			
	Policy makers or authorities	7	N/A^a^
	Stakeholders from the United Nations, nongovernmental organizations, research organizations, media	7	N/A
**Subnational levels (state, region, or province)**			
	Policy makers or authorities	10	N/A
	Employers (study with maternity protection component)	12	N/A
	Health workers (study with the Code component)	12	N/A
**Women**			
	Pregnant	12	310
	Mothers with children aged 0-5 months	12	310
	Mothers with children aged 6-11 months	12	310
Partners of the interviewed mothers with children aged 0-11 months		12	N/A
Total sample size		Up to 96	930

^a^N/A: not applicable.

We will also collect primary data from pregnant women and mothers with children aged 0-11 months using IDI guides and structured questionnaires; we will collect data from these women’s partners using IDI guides (Table 1). These participants must also be at least 18 years of age and willing to participate in the study.

The sample size for IDIs is based on information saturation. If saturation is achieved sooner, fewer IDIs will be conducted, while if saturation is not achieved, the sample will be expanded [50].

The sample size for the quantitative survey of women was determined by using the simple sampling equation with some adjustment for the design effect [51]. We chose this equation because we seek descriptive information from each group of women in each country, without comparing before and after or across groups. The quantitative sample size is based on an estimated *P* of 50% (largest sample size), confidence level of 95%, absolute precision of 7%, and design effect of 1.5. In each country, the minimum sample size is 310 women for each of the 3 following groups: pregnant women, mothers of children aged 0-5 months, and mothers of children aged 6-11 months. Thus, in each country, we will interview 930 women divided equally across the selected provinces.

**Figure 3:**
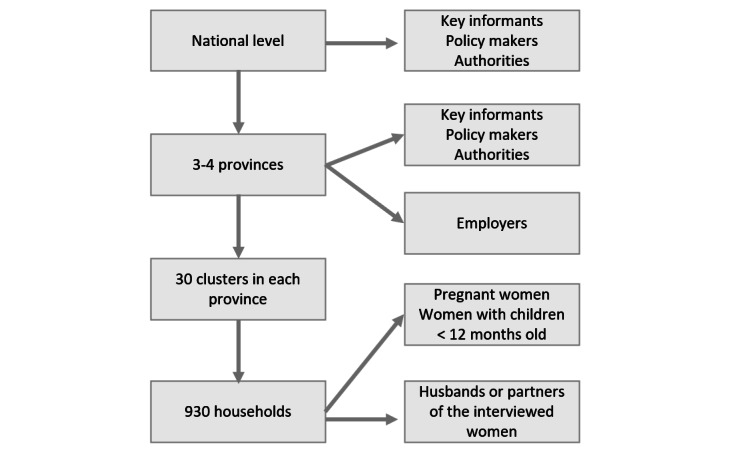
Sampling procedure.

Primary data collection will be conducted at the national, provincial, and community levels (Figure 3). For the quantitative survey in each country, we will use a stratified multistage random sampling procedure, where stratification will be by province, and communities will be selected in the first stage of sampling.

The identification of key informants at the national level will start with member lists of working groups and committees related to food, nutrition, consumer protection, and labor. Key informants for the study of the Code will include representatives from the Ministry of Health, Ministry of Trade, UNICEF, and WHO, among others. Key informants for the study of maternity protection policies include those from the Ministry of Labor, Social Insurance, ILO, UNICEF, and WHO, among others. The key informants will help to identify other potential participants from NGOs, research institutions, and media at the national level.

At the provincial level, research firms will identify a potential list of provinces in which to conduct the study with the support from national key informants and the Alive & Thrive team. The selection of provinces will largely be based on feasibility considerations. The primary criteria are urban provinces or provinces with industrial zones with female workers and research firms that have good connections and working relationships in these provinces. Provinces will be excluded if they are deemed not feasible or inefficient for study conduct, such as remote provinces with low population density or those that are not safe for the research teams. Provinces that generate income mainly from the agricultural or self-employed sectors will not be included because of the lower chance of obtaining a sufficient sample size of formally employed women. In each country, from the list of eligible provinces, the research firm will purposively select 3-4 provinces to improve capture variation by region. We acknowledge that with this sampling strategy, generalizability of the findings will be limited to the selected provinces.

The research firm will contact potential participants by phone to explain the purpose, requirements, and logistics of the survey, and the firm will follow-up with an official letter. The next step entails a kickoff meeting at the provincial level to develop a detailed list of potential participants. The provincial key informants will introduce the research firm and help to schedule an appropriate time and place for the interview. Informed consent will be obtained by interviewers before the IDI. If the appointment is missed, researchers will be allowed to reschedule up to 2 times.

**Figure 4:**
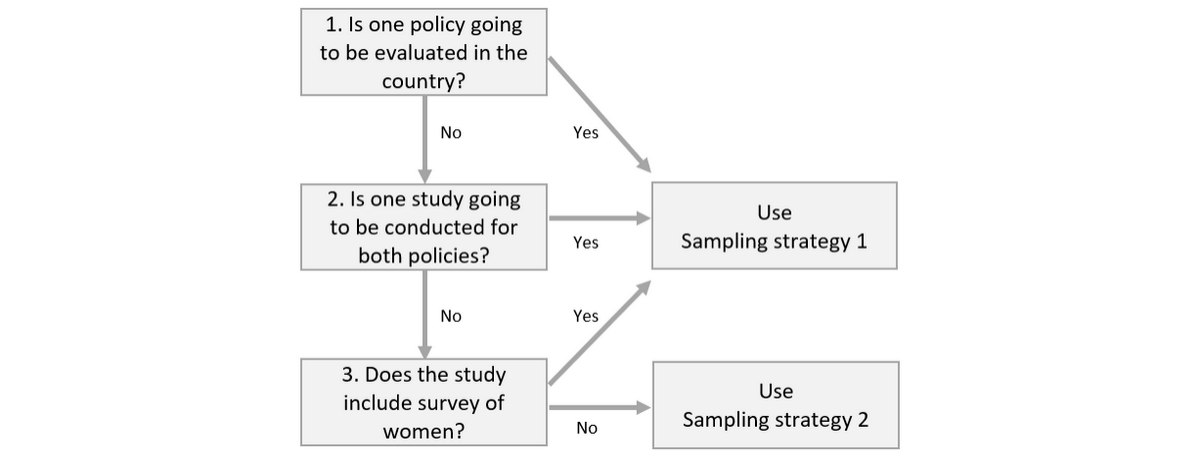
Decision tree for the selection of sampling strategies for women and partners in each province.

At the community level, for each country, researchers will choose which policy(ies) to evaluate and if the study will include both maternity protection and the Code. This decision will be based on each country’s situation and need, availability of funding, and the capacity of the research firm. The community-level sampling strategy relies on this decision (Figure 4). Specifically, if a study includes survey of women, we will use sampling strategy 1, while if a study includes only IDI, we will use sampling strategy 2.

For sampling strategy 1 (with a survey of women), from each of the selected provinces, we will employ a multiple-stage cluster sampling design in each province. Stage 1 is the selection of districts. We will select 3-4 districts in each province using a simple random selection technique. Stage 2 is the selection of clusters and primary sampling units (PSUs). We will define a sample frame of clusters and use the probability-proportional-to-the-size cluster sampling technique to select 30 clusters in the selected districts. A cluster will be a next lower level of subdistricts, which could be communities, villages, or population groups depending on the country context. The proposed minimum number of mothers of children in each PSU is about 1.5 to 2 times the sample size needed in each PSU to ensure we have a sufficient number of participants. Stage 3 is the selection of participants. We will select pregnant women and mothers of children aged 0-5 and 6-11 months in each cluster using systematic random sampling. In selected clusters, 3 lists of potential participants will be created: (1) pregnant women, (2) mothers of children aged 0-5 months, and (3) mothers of children aged 6-11 months. Based on the list and required sample size in each cluster, a list of potential participants will be selected using systematic random sampling.

Based on the list of potential participants, the community representative will call the potential participants to invite them to participate in the study. If they agree to participate, the community representative will introduce participants to the study representative in person and leave the interviewer with the participant. During the interview, the interviewer will introduce himself or herself as well as the purpose of the study, obtain informed consent, and conduct the interview. The interview will be conducted in a quiet, private location that is neither a health facility nor workplace to minimize the participants’ risk of exposure or discomfort. Three attempts to visit the household will be made to complete the interviews with selected and eligible participants. If the interviewers could not meet the participants after 3 attempts, the participants will be replaced with one in the pre-identified list.

A subset of women surveyed will be invited for an IDI. Also, a subset of partners of mothers with children 0-11 months old will be invited to participate in an IDI. After completing the interview using the structured questionnaire, the interviewers will propose women or partners for an IDI, and the team leader will make the final decision of whom to invite for an IDI. The selection of women or their partners for an IDI will be based on the potential for increasing our understanding of how policies impact breastfeeding practices and collecting case studies. For example, IDIs with women will be conducted among those who (1) went back to work before completing maternity leave or completed maternity leave entitlement before returning to work, (2) weaned her child before or upon return to work or continued breastfeeding after returning to work, (3) experienced discrimination at work due to her pregnancy or birth or did not experience discrimination, or (4) knew the benefit of breastfeeding but stopped breastfeeding early and used infant formula or continued breastfeeding and did not use infant formula.

Sampling strategy 2 is for an IDI-only evaluation study (no survey of women). In each country, we will conduct IDIs with 12 pregnant women, 24 mothers of children aged 0-11 months, and 12 fathers of children aged 0-11 months (Table 1). In each of the 3-4 selected provinces, we will select 2-3 districts and 1 subdistrict from each district. Prior to the research team traveling to the field, they will share the eligibility criteria with a pre-identified community representative. In addition to the general eligibility criteria, we will seek women and partners of women who (1) went back to work before completing maternity leave or completed maternity leave entitlement before returning to work, (2) weaned her child before or upon return to work or continued breastfeeding after returning to work, or (3) experienced discrimination at work due to her pregnancy or birth or did not experience discrimination. The community representative will contact potential participants meeting the criteria to invite them to participate in the study. The community representative will make a list of those who agree to participate and schedule the day and time of the interview. The procedure for conducting the interview will be the same as in sampling strategy 1.

#### Study Contents and Variables

**Figure 5:**
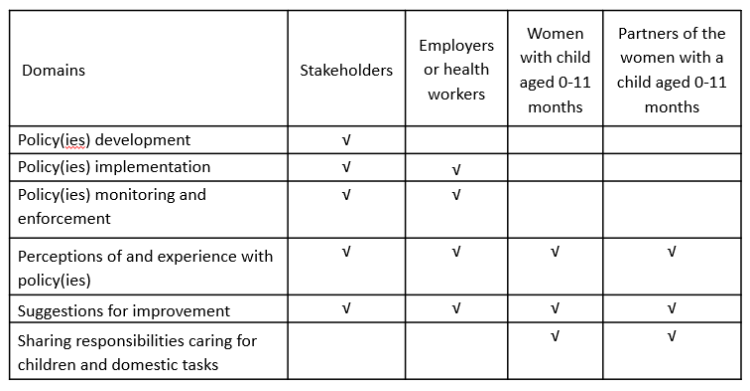
Domains of the in-depth interviews by study participant type.

Regarding the qualitative components, IDI domains are summarized in Figure 5 for each group of participants. The IDI guides were developed based on study objectives and research questions. Complete IDI guides are in Multimedia Appendix 2. Using projective techniques, interviews entail asking participants questions about images they are shown that relate to the study questions. These images will be adapted to each country context. Multimedia Appendix 3 has images designed to facilitate IDIs in Vietnam.

IDIs with key informants will explore implementation, monitoring, and enforcement of the policies; bottlenecks for implementation; and proposed solutions. IDIs with employers will explore their perceptions about the benefits and disadvantages of the policies for businesses and employees.

The purpose of the IDIs with pregnant women and mothers of children aged 0-11 months is to gain deeper insight into respondents’ understanding and perceptions of policies related to maternity protection and the Code including perceived benefits and challenges as they relate to study outcomes (at individual, family, employer, society levels) such as challenges of continuing breastfeeding when returning to work, benefits of maternity protection policies on continued breastfeeding, and changes in breastfeeding practices due to exposure to BMS products.

The purpose of the IDIs with partners of the interviewed pregnant women and mothers of children aged 0-11 months is to learn about respondents’ understanding and perceptions of policies related to maternity protection and the Code as well as paternity leave and the role of partners in taking care of children and in domestic tasks.

For the quantitative survey, we adapted questionnaires from other studies such as NetCode [52], ILO Maternity Protection [28], MICS [53], checklists and questionnaires for early essential newborn care [54,55], and a study by Alive & Thrive on the impact of interpersonal and mass media on breastfeeding practices and an online assessment on the perception of employed women on maternity protection [56-58].

We developed separate questionnaires for pregnant women (Multimedia Appendix 4) and women with children aged 0-11 months (Multimedia Appendix 5) with quite similar contents and structures. The 6 main sections are (1) background information, (2) perinatal care, (3) behavioral determinants (intentions, knowledge, beliefs, social norms, and self-efficacy), (4) media exposure and participation in social group and events, 5) exposure to BMS promotion, and (6) maternity protection (only asked of formally employed women). The questionnaire for women with a child aged 0-11 months has additional information related to the child’s characteristics, delivery, and postnatal care and the benefit of maternity protection received after birth (duration of paid leave, cash and medical benefits, work protection, breastfeeding upon return to work).

We pretested the data collection tools. IDI guides and survey questionnaires were initially developed in English and will be translated into each country’s official language. Another translator will back-translate questionnaires into English to ensure that the content and spirit of the original questions are maintained. Data collection tools will be pretested in face-to-face interviews with at least 2 pregnant women, 2 mothers with children aged 0-11 months (using survey questionnaire and IDI guides), and 2 fathers of children aged 0-11 months (using IDI guides). IDI guides for other participants will be reviewed by an external experienced researcher. Necessary modifications will be made based on feedback from pretesting

### Data Collection

#### Data Collection Team Formation and Training

Data collection teams formed by local research firms will be responsible for data collection. Each team will include one field supervisor and interviewers. ​​​Research firms will identify potential interviewers from a pool of candidates and select interviewers by screening curriculum vitae and conducting interviews.

Before data collection, the principal investigator (PI) of the research firms and the FHI 360 PI will train all interviewers and field supervisors for 4 days on the purpose of the study, terminology, concepts, data collection methods, tool pretesting, and reflection. The training will cover skills, including interview techniques, confidentiality, and privacy, as well as afford the study team the opportunity to practice administering the questionnaires and IDI guides. The training will ensure all interviewers and field supervisors understand the procedures and follow the standardized guidelines to guarantee data quality.

#### Informed Consent

A trained interviewer will explain the research study in detail, including the study objective and potential risks and benefits of participation, and obtain oral informed consent from the participants before any study procedures are performed (Multimedia Appendix 6). The consent form will then be signed by the researcher. Except for interviews using phone or mobile apps, the informed consent form will be printed and given to potential research participants. Interviews may be conducted by phone or mobile apps with national stakeholders. In this case, interviewers will share the informed consent form (eg, by email, shared screen) and obtain a digital signature before conducting the interview. All paper-based signed consent forms will be kept securely in a locked metal box in the study vehicle — separate from any interview notes or other study documents — and, once transferred to the firm’s main office, will be kept in a locked cabinet with limited access and then will be transferred to Alive & Thrive’s Southeast Asia Office (Hanoi, Vietnam).

#### Personal Identification Number (PIN)

After the informed consent process, participants will be assigned a personal identification number (PIN), which will be used to link all data collected from an individual in each of the research steps. The PIN is not linked with any information that would identify an individual participant. At the household level, the PIN will be first assigned to the woman participating in the survey. If she is selected for an IDI, her PIN will be unchanged. If her partner participates in an IDI, his PIN will be P (for partner) plus the PIN of the woman. This approach will help to link information of the survey questionnaire with the IDI with the women as well as to link information of a surveyed woman with her partner.

#### Interview Conduct

Trained interviewers will conduct the IDIs. We will audio record the interviews (with participant consent). For participants who do not consent to audio recording, the interviewers will take detailed notes during the interview.

For the quantitative survey of women, a computerized-assisted survey instrument will be used. The questionnaire will be set up in a web-based application and run on an Android tablet. The interviewer must ensure the completeness and logical flow of the questionnaire before leaving the household.

At the end of the quantitative interview, if the interviewer finds the woman suitable for an IDI (using criteria shared during the data collector training), he or she will ask if the woman is willing to participate in an IDI. If the woman agrees, the interviewer will ask for a contact telephone number as well as set a tentative date, time, and location for the IDI. After receiving confirmation from the coordinator at the central level, the qualitative interviewer will schedule the IDI. 

To facilitate quality assurance (eg, random checks of questionnaires to assess completeness and consistency), at the end of the quantitative interview, the interviewer will ask for the telephone number of the participant. Before asking for the telephone number, the interviewer will confirm that the telephone number will only be used to contact the woman in a rare case when the research team needs to clarify certain information provided or to ask about her interview experience. The interviewer will emphasize that providing the telephone number is optional.

### Data Management

#### IDI Data

The name of audio files collected during IDIs will be linked with the respondent’s PIN. After each day, the audio files will be uploaded to password-protected computers and a secure cloud account. Research assistants from the research firm will transcribe the tapes in the local language. The analytical memos for IDIs will be translated to English to support the report writing. At a later stage, the transcriptions and notes will be translated into English for further analysis. The completed text files will be input to Microsoft Word files and uploaded to NVivo for further analysis.

#### Quantitative Survey Data

Qualitative data will be collected and managed using a digital approach. The questionnaire will be converted to a digital data collection form (eg, Open Data Kit). A data management system will be developed in each country by the local research firm and validated by the FHI 360 staff to ensure that data are fit for analysis purposes.

During data collection, interviewers will enter the information directly into a password-protected tablet. When connected to the internet, data will be synchronized to a secure server of the platform provider (eg, Open Data Kit). Synchronization will be done at least once per day. After uploading, the data will be deleted from the tablets. Two back-up tablets will be provided to each data collection team.

In very rare cases when data collection could not be done using tablets, a paper questionnaire will be used. The interviewer will review the questionnaire for completeness before ending the interview. The supervisor will then review and approve the questionnaire. Completed questionnaires will be entered into the tablet within 24 hours in the field, with a note about why the tablet was not used during the interview. The completed paper questionnaires (if any), consent forms, and other study documents will be stored in a locked box in the vehicle during transportation and in the hotel room at night.

Supervisors at the central level will access data from the cloud, perform data checks and cleaning, and provide feedback about data quality to data collection teams within a day. This will identify and address any issues relating to the questionnaire and data collection in a timely manner.

Raw data will be submitted to Alive & Thrive immediately following the end of data collection. An assigned researcher or research assistant will perform required data management (eg, creating indicators from collected variables) prior to data analysis. Any changes in the dataset will be done through program syntaxes to allow for cross-checking and to ensure that findings are reproducible. Corresponding justifications and individuals who approve each change will be documented.

### Data Analysis

#### Primary Quantitative Data Analysis

For maternity protection, we will analyze associations between exposure to maternity protection (eg, taking leave, duration of leave, paid leave, lactation support at work, social norms) and infant and young child feeding practices. For the Code, we will analyze associations between exposure to BMS (eg, advertisement, advice, gift) and infant and young child feeding practices as well as women’s intentions, knowledge, beliefs, social norms, and self-efficacy.

Some data will complement the IDI with women related to perceptions of the maternity protection legislation and the Code. Each estimate will be accompanied by 95% CIs, and the cluster design effect (eg, by PSU) will be accounted for by using complex survey data analysis methods or with the robust option. Sampling weights as appropriate for each stratum (eg, province, woman group) will be calculated and used when combining data from different strata, for example for the overall country-level analysis. For overall and country-specific data analyses, we will use Stata version 15 (StataCorp LLC, College Station, TX).

#### Primary Qualitative Data Analysis

IDI content will be transcribed and analyzed. Transcripts will be produced using notes taken and recorded audio files. The full transcripts, written in the local language, will be analyzed by in-country research teams. Depending on the availability of a data platform, the analysis will be done in NVivo (or a similar program) or by color-coding the text. The transcripts will then be translated into English for further analysis by FHI 360 or Alive & Thrive staff or consultants to facilitate comparison across countries using NVivo.

The main steps will comprise codebook development, double-coding of a sample of transcripts with intercoder agreement checks, finalization of the codebook to reflect this step, and single coding of primary themes of the discussion, coding for subthemes, exploring any emerging themes, calculating the frequency of subthemes by using the matrix method, and drawing out quotes from the coded phrases [59]. The survey team will then produce a summary report of the findings.

### Study Monitoring and Quality Assurance

Study monitoring will be conducted by staff of FHI 360 and the research firms. At FHI 360, the study will be led by the study manager who is responsible for overall study administration, ensuring the availability of study resources (funding and staff) and for providing feedback and final decisions related to execution of the study. PIs will oversee technical aspects of the study, conduct overall monitoring of data collection, and ensure quality as well as review and sign off on key deliverables and coordinate activities at the regional level to ensure consistency across study sites. The PI or co-PI will also be responsible for identifying, documenting, and reporting protocol violations and social harm. Country research staff of Alive & Thrive will manage all aspects of the study in the assigned country and serve as the linkage between FHI 360 and the research firms.

In the national research firm, the study manager is responsible for technical and financial oversight as well as overall study management in accordance with the signed contract. During data collection, field supervisors will be responsible for ensuring that proper informed consent is provided to each participant and that data collection processes are followed in compliance with the protocol. Field supervisors will remain with the data collection team at all times. Field supervisors will carefully check that the consent process is being performed as approved. The PI will check with field supervisors every week. Field supervisors will travel with selected data collection teams to monitor their field work and to answer any questions during data collection, providing feedback to these teams, as needed.

The research study will be conducted in full compliance with the protocol, which will not be amended without prior approval by the PI or co-PI. Protocol violations will be documented on protocol violation reporting forms, and all will be reported to and addressed by the research teams as soon as possible following discovery. They are also responsible to ensure continuous safety monitoring of all research study participants and for alerting the protocol team if unexpected concerns arise. Compliance will also be checked during site visits by the PI or co-PI.

### Ethical Considerations

From study design to the dissemination of findings, we will follow the ethical guidelines of the Helsinki Declaration [60]. The protocol, questionnaires, IDI guides, consent form, other requested documents, and any subsequent modifications will be reviewed and approved by the institutional review boards (IRBs) of FHI 360 and in-country Ethical Committees. The investigators will make safety and progress reports to the local IRB at least annually and within 3 months after termination or completion. After completing the interview, we will give the interviewees a gift or amount of money (US $2-$3) to thank them and recognize their participation in this research.

## Results

This study was funded in June 2018 and approved by the relevant IRBs (FHI 360: April 16, 2019 and May 18, 2020; and Hanoi University of Public Health: December 6, 2019). Data collection will occur on the following date(s): Vietnam: November and December 2019, May and June 2020; the Philippines: projected August 2020; Myanmar and Thailand: pending based on permissions and funding. Expected results are to be published in January 2021. As of July 2020, we had enrolled 1150 participants in Vietnam.

We will analyze the data using a study conceptual model (Figure 2). We will present a comparison of key contents of the policies across countries and against international standards and recommendations as well as a comparison of implementation strategies, coverage, monitoring, and enforcement across countries. We will also present data analyzed using an interrupted time-series approach to quantify the change in the slope or level of selected outcomes before and after the effective date of a new or amended policy. Findings from IDI contents will include themes, subthemes, and quotes from the coded phrases. For the surveys with women, we will present associations between exposure to maternity protections (eg, taking leave, duration of leave, paid leave, lactation support at work, social norms) and infant and young child feeding practices. We will also present associations between exposure to infant formula promotion (eg, advertisement, advice, gifts) and infant and young child feeding practices as well as women’s intentions, knowledge, beliefs, social norms, and self-efficacy.

Key results from the study will then be contextualized within the conceptual model (Figure 2) for further examination of the structural and environmental determinants of breastfeeding behavior, namely how regulation (or not) of marketing and promotion of BMS might affect social and cultural perceptions and social norms around breastfeeding and how maternity protection and working environments support or impede breastfeeding.

The results will be disseminated through meetings with relevant ministries, institutions, and local and international NGOs in the study countries as well as within FHI 360. Study results are intended to be used by ministries and local stakeholders to advocate for relevant policy changes to improve maternity protection and strengthen the Code in the study countries and the Southeast Asia region. Findings will also be disseminated as presentations in scientific forums and in peer-reviewed publications to contribute to the global discourse of nutrition policy making.

## Discussion

### Strengths and Limitations

The unique strengths of this study are that it examines multiple countries, uses mixed methods, and considers 2 main policies relating to maternal, infant, and young child nutrition: the Code and maternity protection. We will collect and analyze various components from perinatal care, breastfeeding practices, behavioral determinants (eg, intentions, trials, adoption, and maintenance), and exposure to various types of interventions. We adapted standardized questionnaires, including those from the WHO NetCode Assessment Module and ILO Maternity Protection Assessment Toolkit to facilitate the comparison with other studies. We structured the research protocol and data collection tools into sections, which will allow their adaptation to other contexts and study purposes. In addition to tools relating to the data collection (Multimedia Appendices 1-6), we also included a PowerPoint presentation to summarize the study (Multimedia Appendix 7).

Yet, the study has limitations, which we recognize. Because the selection of provinces and districts will not be random, our results may only apply to study sites. Further, the cross-sectional design of the survey cannot be used to conclude causal relationships. However, we have increased the plausibility of causality by selecting age ranges for which exposure or covariate variables occurred before or at about the same time as the studied practices. In addition, if available, secondary data of repeated surveys or trend data analysis will be used to strengthen the causality by ensuring temporal criteria (eg, have data before and after the policy enactment).

### Conclusion

This study will increase understanding of the effectiveness of policy interventions to address the structural determinants of breastfeeding, namely national policies on maternity protection and the Code. By assessing the potential impact of these policies and bottlenecks for successful implementation through the perspective of various stakeholders, including policymakers, implementors, and beneficiaries, this study will build an evidence base that can be used to advocate for stronger policy adoption and enforcement in study countries and beyond.

## Appendix

Multimedia Appendix 1 Semi-quantitative policy data extraction form.

Multimedia Appendix 2 In-depth interview guides.

Multimedia Appendix 3 Images to facilitate in-depth interviews.

Multimedia Appendix 4 Survey questionnaire for pregnant women.

Multimedia Appendix 5 Survey questionnaire for women with children aged 0-11 months.

Multimedia Appendix 6 Informed consents.

Multimedia Appendix 7 PowerPoint presentation of the study.

## Abbreviations

BMS: breast milk substitute
DHS: Demographic Health Surveys
IDI: in-depth interview
ILO: International Labor Organization
IRB: Institutional Review Board
MICS: Multiple Indicator Cluster Surveys
NGO: non-governmental organization
PI: principal investigator
PIN: personal identification number
PSU: primary sampling unit
UN: United Nations
UNICEF: United Nations Children’s Fund
WHA: World Health Assembly
WHO: World Health Organization
