# Supplementary material for: Assessing the Effectiveness of Policies Relating to Breastfeeding Promotion, Protection, and Support in Southeast Asia: Protocol for a Mixed Methods Study
Source: JMIR Res Protoc. 2020 Sep 21;9(9):e21286. doi: 10.2196/21286 (PMC7536596; doi:10.2196/21286)
Supplement: Multimedia Appendix 1 [file resprot_v9i9e21286_app1.pdf]

APPENDIX 1. SEMI-QUANTITATIVE DATA EXTRACTION FORM

Reviewer: ……………………………………………. **Review Date:** ……/……/20….

(**Note:** Circle appropriate choice; or fill in number or text)

A. GENERAL INFORMATION ABOUT THE POLICY

A1. Country: 1. Myanmar; 2. the Philippines; 3. Thailand; 4. Vietnam; 9. International policies

A2. Policy basic information:

Policy Name:

Published by:	Year Published:
Adopted by:	Year Adopted:
Start year:	End year:

A3. Available language *(multiple choice)***:**

1. Both local language and English; 2. Local language only; 3. English only

A4. Type of Policy: 1. Maternity protection related policy; 2. BMS Code related policy

A5. Legal status: 1. Voluntary; 2. Mixed; 3. Mandatory; 98. Unclear

A6. Legislation: 1. Constitution; 2. Law, Acts, Codes; 3. Sub-law, decree, orders, regulations; 4. Guidelines, circular, standards; 98. Unclear

A7. Governing Resources: 1. Information or knowledge; 2. Authority; 3. Treasury; 4. Organization structure; 98. Unclear

A8. Categories: 1. Symbolic policy; 2. Material, substantive instrument; 3. Material, procedural instrument; 98. Unclear

Notes: if A4 = 1, complete questions in section B

if A4 = 2, complete questions in section C

B. MATERNITY PROTECTION

Describe the following terms and conditions from the policy:

B1 Eligibility for maternity protection

B2 Maternity protection for employed women

1. Type of leave included:
    - o Sick leave
    - o Annual leave
    - o Marriage leave
2. Type of health protection covered (e.g., health insurance, medical checkup, pregnant or breastfeeding women are not obliged to performed task in hazardous tasks that might affect the mother's health or that of her child):
   ____
3. Duration of paid maternity leave: …………. weeks
4. Benefits paid by:
    - o Social security fund
    - o Employer
    - o Employee
    - o Other
5. Additional leave provided when complication or illness? (Y/N)
6. Employment protection and non-discrimination, including hiring substitute staff, provided? (Y/N)
7. Provide nursing breaks for breastfeeding mothers? If yes:
    - o Break time:……. hours
    - o for the period of ……. months
8. Workplace lactation facility? (Y/N)
9. Daycare facility? (Y/N)

B3. Maternity protection for other groups

1. Paternity leave? (Y/N and provisions)

2. Maternity protection of non-employed women? (Y/N, and provisions, e.g., coverage or support)

Describe the following terms and conditions from the policy:

B4 Related guidelines and regulations

B5 Implementation strategies & coverage

B6 Monitoring of the policy

B7 Enforcement

B8 Changes in the contents of maternity protection policies over time

C. **POLICY IS RELATED TO INTERNATIONAL CODE OF MARKETING OF BREASTMILK SUBSTITUTES**:

C1 Does the policy cover the following items?

1. Milk formula
2. Other milk products (e.g., fresh milk, condensed milk, liquid yogurt)
3. Foods and beverages (e.g., baby cereals, snack, including bottle-fed complementary foods,
4. Feeding bottles and teats

C2 What are milk products under the scope of this policy?

1. Milk for pregnant and lactating women
2. Milk for Newborn
3. Milk for infant < 6 months
4. Milk for infant <12 months
5. Milk for children < 18 months
6. Milk for children < 24 months
7. Milk for children < 36 months

C3 Does the policy indicate acceptable reasons for using BMS?

1. Infants with specific diseases (e.g., galactosemic, maple syrup urine disease, Phenylketonuria)
2. Very low birthweight (< 1500 grams)
3. Very preterm (< 32 weeks of gestational age)
4. Maternal HIV infection

5. Maternal severe illness
6. Maternal medication
7. Other infectious diseases (e.g., Hepatitis B and C and tuberculosis (TB))
8. Mastitis, breast abscess
9. Maternal substance use (e.g., nicotine, alcohol, ecstasy, amphetamines, cocaine, opioids, benzodiazepines and cannabis)
10. Other (specify)

C4 What are components relating to promotion of BMS stated in the policy (mark Y/N and add notes, if needed)?

1. **Article 4. Information and education:**
    1) objective and consistent information is provided on infant and young child feeding;
    2) requirement on informational and educational materials, whether written, audio, or visual, dealing with the feeding of infants and intended to reach pregnant women and mothers of infants and young children;
    3) requirement on donations of informational or educational equipment or materials by manufacturers or distributors
2. **Article 5. The general public and mothers:**
    1) no advertising or other form of promotion to the general public of products within the scope of this Code;
    2) no distribution of samples of products;
    3) no special displays, discount coupons, premiums, special sales, loss-leaders and tie-in sales (at retail level);
    4) no gifts utensils, might promote using BMS;
    5) no marketing personnel to contact pregnant women or mothers of infants and young children
3. **Article 6. Health care systems:**
    1) breastfeeding promotion and protection policies and regulations;
    2) no promotion of BMS;
    3) band BMS representative;
    4) band display of name and company's logo
4. **Article 7. Health workers:**
    1) should encourage and protect breastfeeding;
    2) not use of information provided by company,
    3) no financial or material inducements to promote BMS;
    4) not to provide sample of BMS, equipment, utensils;

5) disclose of benefit from BMS company, including fellowships, study tours, research grants, attendance at professional conferences

5. **Article 8. Persons employed by manufacturers and distributors:**
   1) the volume of sales of BMS products should not be included in the calculation of bonuses,
   2) to perform educational functions in relation to pregnant women or mothers of infants and young children.
6. **Article 9. Labelling:**
   1) information about appropriate use of the product, superior of breastmilk,
   2) products should be used with consultation of health workers,
   3) instruction on preparation and potential harms
7. **Article 10. Quality:**
   1) meet standards recommended by the Codex Alimentarius Commission Foods for Infants and Children
   2) meet standards recommended by the Codex Code of Hygienic Practice for Foods for Infants and Children

Describe the following terms and conditions from the policy:

C5 Related guidelines and regulations

C6 Implementation strategies & coverage

C7 Monitoring of the policy

C8 Enforcement

C9 Changes in the contents of maternity protection policies over time
