# Supplementary material for: Assessing the Effectiveness of Policies Relating to Breastfeeding Promotion, Protection, and Support in Southeast Asia: Protocol for a Mixed Methods Study
Source: JMIR Res Protoc. 2020 Sep 21;9(9):e21286. doi: 10.2196/21286 (PMC7536596; doi:10.2196/21286)
Supplement: Multimedia Appendix 2 [file resprot_v9i9e21286_app2.pdf]

APPENDIX 2. SUGGESTED IDI GUIDES

Contents

IDI Guide: Policy makers and authorities (National and Subnational) 2

IDI Guide: Key Stakeholders 5

IDI Guide: Employers.......... 8

IDI Guide: Health Workers.......... 11

IDI Guide: Pregnant Women or Women with children < 12 months old 14

IDI Guide: Partners of the interviewed mothers or Fathers with children < 12 months 21

IDI Guide: Policy makers and authorities (National and Subnational)

[Note: This guide will need to be adjusted to each country context to reflect the current policy situation.]

Introduction

[Use this text if there is a delay between the consent process and the interview. Otherwise, thank the participant again for making time to speak with you and use the second paragraph to start the interview.]

My name is [x] and I come from [x]. Thank you for taking time to talk with me today. I asked to speak with you because you play an important role in breastfeeding policymaking and/or implementation in your country and I would like to learn from you about your experience and your expectations going forward.

Our time together may last about 1 hour. I will ask you some questions, but I will not say much because I want to learn from you. The questions have no right or wrong answers. I only ask that you share what you truly think and feel.

Warm up

1. Would you please tell me about yourself and your role in this organization?
    a. How long you have worked with your organization?
    b. Please describe briefly your role in your organization?

Breastfeeding Policies and Strategies/Programs

2. What policies and strategies/programs are currently in place to protect and support breastfeeding in [Country name]?

Today we will focus on two policies that impact breastfeeding: maternity protection and the Code of Marketing of Breastmilk substitutes (use local names for policies).

[Probe using the following questions, asking one-by-one for each policy]
- What was/is your/your organization's role in developing these policies?
- In your opinion, are they sufficiently improving breastfeeding practices? (probe for: benefits of the policies)
- If no: How should they be strengthened? (probe for: weaknesses in the current policies)
- If yes: in what ways do you see that they are doing so?

3. Are these policies being implemented? *[Ask one-by-one for each policy]*

[Probe using the following questions:]
- If yes, how is your country able to do so? (probe: how moved from policy execution to implementation, funding source, roles/responsibilities for implementation, coverage)
- If not, what are the barriers to doing so? (probe: technical capacity, funding)

  - What could be done to overcome these barriers? Who needs to take action? *[Probe for each barrier mentioned]*
- What is your/your organization's role in implementing these policies?

4. Are these policies being monitored? *[Ask one-by-one for each policy]*

   [Probe using the following questions:]
   - If yes, how is your country able to do so? (probe: what are the monitoring mechanisms, funding source, roles/responsibilities, coverage)
   - If not, what are the barriers to doing so? (probe: lack of a system, technical capacity, funding)
     - What could be done to overcome these barriers? Who needs to take action? *[Probe for each barrier mentioned]*
   - What was your/your organization's role in monitoring these policies?

5. Are these policies being enforced? *[Ask one-by-one for each policy]*

   [Probe using the following questions:]
   - If yes, how is your country able to do so? (probe: what are the enforcement mechanisms, funding source, roles/responsibilities, coverage)
   - If not, what are the barriers to doing so? (probe: lack of a designated cadre for enforcement, companies not agreeing, BMS companies practices, technical capacity, funding)
     - What could be done to overcome these barriers? Who needs to take action? *[Probe for each barrier mentioned]*
   - What was your/your organization's role in enforcing these policies?

6. Although there are some policies in place, how should these policies be strengthened to better promote, protect and support breastfeeding?

   [Probe using the following questions:]
   - Why isn't this yet happening?
   - What is needed to put this in place?
   - Who can do so?

7. What additional policies or strategies/programs needed to promote, protect and support breastfeeding?

[Probe using the following questions:]
- Why aren't they yet in place?
- What is needed to put this in place?
- Who can do so?

8. In your opinion, who should be responsible for developing breastfeeding policies and strategies that currently is not being held accountable?

[Probe using the following questions:]

- What steps could be taken to make them be accountable? Who has the power to do so?

9. In your opinion, who should be responsible for monitoring and enforcing BF policies and strategies that currently is not being held accountable?

[Probe using the following questions:]

- What steps could be taken to make them be accountable? Who has the power to do so?

10. Who is responsible for ensuring adequate financial support for implementation, monitoring and enforcement of breastfeeding policies and strategies within government budgets?

[Probe using the following questions:]

- What steps could be taken to make them be accountable? Who has the power to do so?

Closing

Thank you so much for sharing your thoughts with me. I have learned so much. Before we close, is there anything else you would like to tell me about the topics we discussed today? *[Record all questions]*

Give participant a chance to say more and then thank the participant again. If s/he has nothing more to say, thank her/him and close.

IDI Guide: Key Stakeholders

[Note: This guide will need to be adjusted to each country context to reflect the current policy situation.]

Introduction

[Use this text if there is a delay between the consent process and the interview. Otherwise, thank the participant again for making time to speak with you and use the second paragraph to start the interview.]

My name is [x] and I come from [x]. Thank you for taking time to talk with me today. I asked to speak with you because you play an important role in breastfeeding policymaking and/or programming in [name country] and I would like to learn from you about your experience and your expectations going forward.

Our time together may last about 1 hour. I will ask you some questions, but I will not say much because I want to learn from you. The questions have no right or wrong answers. I only ask that you share what you truly think and feel.

Warm up

11. Would you please tell me about yourself and your role in this organization?
    a. How long you have worked with your organization?
    b. Please describe briefly your role in your organization?

Breastfeeding Policies and Strategies/Programs

12. What policies and strategies/programs are currently in place to protect and support breastfeeding in [name country]?

Today we will focus on two policies that impact breastfeeding: maternity protection and the Code of Marketing of Breastmilk substitutes (use local names for policies).

[Probe using the following questions, asking one-by-one for each policy]
- What was/is your/your organization's role in developing these policies?
- In your opinion, are they sufficiently improving breastfeeding practices? (probe for: benefits of the policies)
- If no: How should they be strengthened? (probe for: weaknesses in the current policies)
- If yes: in what ways do you see that they are doing so?

13. Are these policies being implemented? *[Ask one-by-one for each policy]*

[Probe using the following questions:]
- If yes, how is [name country] able to do so? (probe: how moved from policy execution to implementation, funding source, roles/responsibilities for implementation, coverage)
- If not, what are the barriers to doing so? (probe: technical capacity, funding)
    - What could be done to overcome these barriers? Who needs to take action? *[Probe for each barrier mentioned]*
- What is your/your organization's role in implementing these policies?

14. Are these policies being monitored? *[Ask one-by-one for each policy]*

[Probe using the following questions:]

- If yes, how is [name country] able to do so? (probe: what are the monitoring mechanisms, funding source, roles/responsibilities, coverage)
- If not, what are the barriers to doing so? (probe: lack of a system, technical capacity, funding)
    - What could be done to overcome these barriers? Who needs to take action? *[Probe for each barrier mentioned]*
- What was your/your organization's role in monitoring these policies?

15. Are these policies being enforced? *[Ask one-by-one for each policy]*

    [Probe using the following questions:]

    - If yes, how is [name country] able to do so? (probe: what are the enforcement mechanisms, funding source, roles/responsibilities, coverage)
    - If not, what are the barriers to doing so? (probe: lack of a designated cadre for enforcement, companies not agreeing, BMS companies practices, technical capacity, funding)
        - What could be done to overcome these barriers? Who needs to take action? *[Probe for each barrier mentioned]*
    - What was your/your organization's role in enforcing these policies (if any)?

16. Although there are some policies in place, how should these policies be strengthened to better promote, protect and support breastfeeding?

    [Probe using the following questions:]

    - Why isn't this yet happening?
    - What is needed to put this in place?
    - Who can do so?

17. What additional policies or strategies/programs needed to promote, protect and support breastfeeding?

[Probe using the following questions:]

- Why aren't they yet in place?
- What is needed to put this in place?
- Who can do so?

18. In your opinion, who should be responsible for developing breastfeeding policies and strategies that currently is not being held accountable?

[Probe using the following questions:]

- What steps could be taken to make them be accountable? Who has the power to do so?

19. In your opinion, who should be responsible for monitoring and enforcing breastfeeding policies and strategies that currently is not being held accountable?

[Probe using the following questions:]

- What steps could be taken to make them be accountable? Who has the power to do so?

20. Who is responsible for ensuring adequate financial support for implementation, monitoring and enforcement of breastfeeding policies and strategies within government budgets?

[Probe using the following questions:]

- What steps could be taken to make them be accountable? Who has the power to do so?

Closing

Thank you so much for sharing your thoughts with me. I have learned so much. Before we close, is there anything else you would like to tell me about the topics we discussed today? *[Record all questions]*

Give participant a chance to say more and then thank the participant again. If s/he has nothing more to say, thank her/him and close.

IDI Guide: Employers

Introduction

[Use this text if there is a delay between the consent process and the interview. Otherwise, thank the participant again for making time to speak with you and use the second paragraph to start the interview.]

My name is [x] and I come from [x]. Thank you for taking time to talk with me today. I asked to speak with you because I'm interested in learning about how women can continue to breastfeed their children after returning to work and you run a business with at least 50 female employees.

Our time together may last about 1 hour. I will ask you some questions, but I will not say much because I want to learn from you. The questions have no right or wrong answers. I only ask that you share what you truly think and feel.

Warm up

21. Would you please tell me a bit about yourself?
    a. Please describe the company for me.
    b. Please describe briefly your role in this company.
    c. How long you been involved in this company?

Feeding Practices and Maternity Protections

Image-based Discussion

[Explain the process by saying:]

I'd like to show you some images and ask you questions about them.

[Show an image of a pregnant woman and ask:]

1. Imagine this woman is working.
    a. What kind of problems do working pregnant women face? (probe for: discrimination, being let go by her employer, lack of peer support, lack of employer support, no benefits)
    b. What can companies do to support her to keep working until delivery?
    c. What can companies do to help her return to work, but still take care of her baby?

[Show an image of a woman working in the type of business of the study participant and ask:]

2. Imagine this woman returned to work when her child is less than [number of months indicated in the maternity legislation] months.
    a. How does she feed her baby? Why? (probe for: feeding breaks, express milk and take home)
    b. Why does she go back to work at that time? (probe for key reasons or considerations from employee, employer, and their negotiation).

c. Would this woman receive maternity leave or benefits *[explain as needed]*? Why or why not? What kinds of benefits?
    i. What is the maternity leave she is entitled to?
    ii. What benefits is she entitled to (include both cash and non-cash benefits)?

3. What role do companies like yours have in making decisions about maternity leave and benefits?

4. What role do companies like yours have in monitoring maternity leave policies?

5. What happens if companies do not follow maternity leave policies? Who enforces these policies?

6. Now I'd like to talk with you about your perceptions of maternity leave and benefits policies. Let's start with the positives.

    a. In your opinion, what are the "pros" of maternity leave and benefits policies? (probe for: happier workers, less turnover, healthy children) *[Write each response on a separate piece of paper / sticky note.]*
    b. Now I'd like you to rank the pros you mentioned from most to least important. Which of these are most important? Next? *[Repeat this process, until all the paper / sticky notes are organized from most to least important. Write down this sequence or take a photo and wrote a note PHOTO [title]. The time of photo taken, and the title will be used to pair the photo with the IDI transcript.]*

    [Repeat this process, but for the cons by asking:]

    c. In your opinion, what are the "cons" of maternity leave and benefits policies? (probe for: cost) *[Write each response on a separate piece of paper / sticky note.]*
    d. Now I'd like you to rank the cons you mentioned from most to least important. Which of these are most important? Next? *[Repeat this process, until all the paper / sticky notes are organized from most to least important. Write down this sequence or take a photo and wrote a note PHOTO [title]. The time of photo taken, and the title will be used to pair the photo with the IDI transcript.]*

    e. You said [x] is the biggest pro and you said [x] is the biggest con. In your opinion, does the pro outweigh the con or is the con too great? Why?

[Before showing an image of a woman in a nursing room in a company eexpressing milk and ask:]

7. I'm interested to learn about what happens when a woman returns to work. If a woman wanted to continue breastfeeding while working, how could she do that?

[Now show the image:]

8. [Women name] returned to work after her maternity leave. She is expressing milk in a lactation room in the company.
    a. In what kinds of companies does this happen?
    b. How is this able to happen?
    c. What are the barriers your company would face in doing so?

d. What do you see as the benefits to doing so?
e. What do you think your company could offer to women working here who want to continue breastfeeding until their child reaches two years of age?

9. Where would a working woman get information on maternity leave and benefits?

10. What information does your company provide to its workers about maternity leave and benefits? (probe for channels)

Closing

Thank you so much for sharing your thoughts with me. I have learned so much about your thoughts and experience. Before we close, is there anything else you would like to tell me about the topics we discussed today? *[Record all questions]*

Give participant a chance to say more and then thank the participant again. If s/he has nothing more to say, thank her/him and close.

IDI Guide: Health Workers

Introduction

[Use this text if there is a delay between the consent process and the interview. Otherwise, thank the participant again for making time to speak with you and use the second paragraph to start the interview.]

My name is [x] and I come from [x]. Thank you for taking time to talk with me today. I asked to speak with you because you play an important role in supporting women and children's health and nutrition and I would like to learn from you about your experience.

Our time together may last about 1 hour. I will ask you some questions, but I will not say much because I want to learn from you. The questions have no right or wrong answers. I only ask that you share what you truly think and feel.

Warm up

22. Would you please tell me a bit about yourself?
    d. Please describe the facility for me.
    e. Please describe briefly your role in this facility.
    f. How long you been working in this facility?

Feeding Practices and the Code

Part A: Image-based Discussion

[Explain the process by saying:]

I'd like to show you some images and ask you questions about them.

[Show an image of a ***newborn child*** *and ask:]*

- What is important for a newborn child like this one? (Probe for: ceremonies; breastfeeding, food/drinks; play; affection; health care)
- What is important for the mother of a child of this age? (Probe for: support to eat more and the right foods – which foods, social support, etc.)
- How soon after delivery do you think the child was breastfed? Why do you say that?
- Some children take liquids or food first, before breastmilk, right? Why? (Probe for: availability of BMS in or nearby the facility; recommendation of health workers)
- What could health workers do to support mothers to breastfeed the child immediately after birth, and not give other liquids or food first? (probe for personal experience – what does the HW do to support mothers?)

[Show images of the ***6, 12 and 22-months-old Children*** *and ask:]*

- Here are three children. One is 6, one is 12 and one is 22 months old. What is important for children of these ages? (Probe for: ceremonies; breastfeeding, food/drinks; play; affection; health care)

- Do you think these children are still breastfed? Why do you say that?
- Some children stop breastfeeding before the age of 2, right? Why?
- What could health workers do to support mothers to continue to breastfeed until 2 years? (probe for personal experience – what does the HW do to support mothers?)

Part 1B: Story
Now I will tell a short story. Please help me to finish the story by answering some questions after each part.

[Popular woman's name in the country] had a baby! Although she felt tired, she put the baby on her chest, and hoped the baby would take breastmilk. When she said she did not feel the milk coming in, a health worker suggested that she give the baby infant formula while [woman's name] rested.

a. Does this happen here sometimes?
    i. Why or why not?
b. Did you ever receive information about formula milk? If so, where? From whom?
c. Do you know about any laws or policies regulating infant formula in health facilities? (If "no", skip to question iii)
    i. **If yes,** please explain what you know. (probe for: perceptions of the regulation, ease of following, challenges in following)
    ii. What do you think are the "pros" and "cons" of this policy?

        1. First, in your opinion, what are the "pros"? *[Write each response on a separate piece of paper / sticky note.]*
        2. Now I'd like you to rank the pros you mentioned from most to least important. Which of these are most important? Next? *[Repeat this process, until all the paper / sticky notes are organized from most to least important. Write down this sequence or take a photo and wrote a note PHOTO [title]. The time of photo taken, and the title will be used to pair the photo with the IDI transcript.]*

        [Repeat this process, but for the cons by asking:]

        3. In your opinion, what are the "cons" of maternity leave and benefits policies? *[Write each response on a separate piece of paper / sticky note.]*
        4. Now I'd like you to rank the cons you mentioned from most to least important. Which of these are most important? Next? *[Repeat this process, until all the paper / sticky notes are organized from most to least important. Write down this sequence or take a photo and wrote a note PHOTO [title]. The time of photo taken, and the title will be used to pair the photo with the IDI transcript.]*

    iii. **If no,** *[explain the country's regulation and ask:]* what do you think of this regulation? (probe for: agreement with it, ease of following, challenges in following)

[Then say:] I'd now like to continue the story.

In town [women name] sees billboards and other advertisements for formula milk. She also sees commercials on TV that say these milks can make children strong and smart. But the midwife says that breastfeeding is best. [Woman's name] feels confused about this.

a. Does this happen here sometimes?
    iv. Why or why not?
b. Do you know about any laws or policies regulating advertising like this? If so, please explain what you know.
    i. If so, please explain what you know. (probe for: perceptions of the regulation, ease of following, challenges in following)
    ii. If not, [explain the country's regulation and ask]: what do you think of this regulation? (probe for: agreement with it, ease of following, challenges in following)
c. As a health worker, do you have any role in monitoring this policy? If so, please describe.

Closing

Thank you so much for sharing your thoughts with me. I have learned so much about your thoughts and experience. Before we close, is there anything else you would like to tell me about the topics we discussed today? *[Record all questions]*

Give participant a chance to say more and then thank the participant again. If s/he has nothing more to say, thank her/him and close.

IDI Guide: Pregnant Women or Women with children < 12 months old

Guiding Research Questions for Pregnant Women and women with children < 12 months

- How do women manage work and child feeding and care?
- What are women's experiences receiving maternity leave and benefits?
- What are perceptions of maternity leave and benefits?
- How do women make decisions about breastfeeding?
- What are current perceptions and practices, including barriers and motivations, of early initiation of breastfeeding, and skin-to-skin contact and opportunities to increase?
- What are current perceptions and practices, including barriers and motivations, of exclusive breastfeeding, and opportunities to increase?
- What are current perceptions and practices, including barriers and motivations, of continued breastfeeding, and opportunities and facilitators to lengthen duration?
- What are current perceptions and practices related to feeding young children breastmilk substitutes and milk products?
- What are women's expectations of their roles and responsibilities?
- Who influences mothers, and by which communication channels or social networks?
- What are opportunities to support women's empowerment and participation?

Introduction

[Use this text if there is a delay between the consent process as well as the survey interview. Otherwise, thank the participant again for making time to speak with you and use the second paragraph to start the interview.]

Hello! My name is [x] and I come from [x]. Thank you for taking time to talk with me today! Our time together may last about 1 hour. I will ask you some questions. I will not say much because I want you to do all the talking; I care about your answers and I want to learn from you. The questions have no right or wrong answers. I only ask that you share what you truly think and feel.

Your answers will represent the thoughts and feelings of hundreds of women just like you, and those women want you to speak for them. Your ideas will help us to strengthen policies and programs that are useful for all women and their children.

[After a self-introduction, ask:]

Please tell me a bit about yourself, your age, how many children you have and their ages and who lives in your home.

[After she introduces herself to you, ask the following questions – note the questions for pregnant women vs mothers of children < 12 months. If the interview is right after the survey, you don't need to ask the questions, but can instead confirm the following information with the woman.]

Introductory questions for pregnant women	***Introductory questions for mothers of children under 12 months***
1. When is your baby due?<br>2. How are you feeling about the pregnancy?	1. How old is your youngest child?<br>2. Is this child a girl or boy?

3. How are you preparing for your baby at home?	3. Who helps you with your child?<br>a. What do they do with the child? (probe: play, feed, bathe, dress)<br>b. When/ how often?
[Then say:] Let's talk about your plans for your new baby today, though first I'd like to show you some photos and share a story and get your ideas about what you see and hear. The focus of my questions is on breastfeeding.	*[Then say:]* Let's talk about this child during our discussion today, though first I'd like to show you some photos and share a story and get your ideas about what you see and hear. The focus of my questions is on breastfeeding.

Depending on 1) participant type (pregnant women or women with children < 12 months), 2) type of policy evaluated (Maternity protection or BMS Code), and 3) responses of the women in the survey, interviewers might select different parts of this IDI guide. The detail instruction on how to select the topics will be covered in the training as well as depend on the saturation of information.

Part 1: Perceptions of Breastfeeding: Ask to all women

Part 1A: Photo-based Discussion

Here are some photos. I'd like to ask you a few questions about them. As you answer, feel free to share your own experiences if you feel comfortable.

[Show the photos of different types women (young, old, modern, old-fashioned) and ask the following questions for each photo:]

1. How does this woman feed her baby? Why?
    a. Do you think she feels happy sometimes? What makes this woman feel happy?
    b. Do you think she feels sad or confused sometimes? When and why?

Part 1B: Story
Now I will tell a short story. Please help me to finish the story by answering some questions after each part of the story. Please feel free to share your own experiences, if you feel comfortable.

> *[Woman name popular in the country] had a baby! Although she felt tired, she put the baby on her chest, and hoped the baby would take breastmilk. When she said she did not feel the milk coming in, a health worker suggested that she give the baby infant formula while [Woman name] rested. Later the health worker brought the baby to [Woman name] to breastfeed. But, [Woman name] was unsure if her milk was enough for the baby.*

d. Does this happen here sometimes?
    i. Why or why not?
    (Probe for: confidence of mother, influence of others)
    ii. When does it happen?
    (Probe for: place of delivery, experience of mothers)
e. Do you think the health worker was right to give that advice?
f. Do some mothers give only breastmilk after birth? How is that possible?

g. What do you think [Woman name] should do?
h. Where could [Woman name] get advice about breastfeeding?

After birth, [Woman name] stayed home from work and gave the baby only breastmilk-- no other food or drinks. Then, after 2 months, [Woman name] returned to work at a shop in a town nearby. Sometimes, when she went out, her mother-in-law cared for the baby and gave the baby infant formula.

a. Does this happen here sometimes?
    i. Why or why not?
b. What liquids are children given before 6 months?
c. What foods are children given before 6 months?
d. What do you think is the best way to feed a baby under 6 months?
e. What are the advantages of breastfeeding? What are the disadvantages (if any)?
f. What are the advantages of formula feeding? What are the disadvantages (if any)?
g. Do some mothers wait until the child is 6 months to give other water or food? How can they do this?

In the town where [Woman name] works, she sees billboards and other advertisements for formula milk. When she watches TV in the evening, [Woman name] sees commercials for formula milk that say these milks can make children strong and smart. But the midwife says that breastfeeding is best. [Woman name] feels confused about this.

d. Does this happen here sometimes?
    ii. Why or why not?
e. Do you know about any laws or policies regulating advertising like this? If so, please explain what you know.
f. Did you ever receive information about formula milk? If so, where? From whom?

When the child of [Woman name] was over 1 year, she stopped giving breastmilk. The child eventually stopped asking. The family bought formula milk for the child and gave that instead.

a. Does this happen here sometimes?
    iii. Why do mothers stop breastfeeding?
    iv. When is it more likely to happen?
    v. How do you think [Woman name] felt when the child stopped breastfeeding?
    vi. Do some families give formula milk powder or other milk? Why? Where is it purchased? How is it prepared?
b. Could [Woman name] find a way to continue breastfeeding until the child reaches 2 years or older?
    vii. Why or why not?
    viii. How could she do this?
c. How could her family support her?
d. Do some mothers here breastfeed the child until 2 years? How is this possible?

Part 1C: Experiences of pregnant women and mothers

As I mentioned earlier, I would now like to ask you about you -- your plans for breastfeeding your baby/ your breastfeeding practices with child [name].

Pregnant women	***Mothers of children under-12 months***
1. Do you plan on breastfeeding? Why or why not? How did you make this decision?<br>2. How long do you plan on breastfeeding? Why did you make this plan? (probe for advice received from health worker, mother-in-law, friend)<br>3. Did you receive any information about breastfeeding? If yes, what kind of information? From whom?<br>4. Do you plan on feeding your baby formula? Why or why not? If yes, when do you plan to start?	1. Are you breastfeeding your child now?<br>a. If yes, how long do you plan to continue?<br>b. If no, did you ever breastfeed your child? Why or why not? When did you stop breastfeeding? Why? Did anyone help you make this decision?<br>2. Have you ever given formula milk to your child?<br>a. If yes, when? How did you make this decision? Did anyone influence your decision? How?<br>b. If no, how did you make this decision? Did anyone influence your decision? How?<br>3. Did anyone provide you with information about breastfeeding? If yes, who? What kind of information?<br>4. How does child feeding affect the way you manage household tasks? Is your partner involved?

Part 2: Employment

Part 2A: Photo-based Discussion

I'm now going to show you some more photos and I'd like to ask you questions about them. Please feel free to share your own experience if you feel comfortable.

Show photos of women working in different settings (factory, shop, agriculture/plantation, market, tailor, etc.) and ask the following questions for each photo:

11. How does this woman feed her baby? Why?
    a. Do you think she feels happy sometimes? What makes this woman feel happy?
    b. Do you think she feels sad or confused sometimes? When and why?

12. Would this woman receive maternity leave or benefits *[explain as needed]*? Why or why not? What kinds of benefits?

13. Where would she go for information on maternity leave and benefits?

14. If she wants to continue breastfeeding while working, how could she do that?

Part 2B: Experiences of pregnant women and mothers

Like before, when I asked you questions about your plans or experience, I would like to ask you some more questions.

<table>
<tr><th>Pregnant women</th><th>Mothers of children under-12 months</th></tr>
<tr><td>
1. Are you currently working? [If no, skip this section]
2. Do you plan to return to work after the birth of your child? Why or why not?
3. Have you received information about maternity leave? If yes, what kind of information? From whom did you receive this information?
4. Will you take maternity leave? Why or why not?
5. What benefits will you receive during maternity leave? What benefits will you receive after maternity leave?
6. How do you plan to feed your baby once you return to work? How did you make this decision?
7. Who will care for your baby while you are at work?
8. Have you ever experienced discrimination at work? If yes, what kind of discrimination?
</td><td>
1. Did you work before the birth of your child, or are you currently working?
2. Did you receive any information about maternity leave or benefits prior to the birth of your child? If yes, what kind of information? From whom did you receive this information?
3. Did you utilize the full duration of maternity leave provided to you by your employer? Why or why not?
4. Did you receive full cash and non-cash benefits available to you under your employer's maternity protection policy? Why or why not? Can you describe the benefits you received?
5. Is there anyone at your workplace that monitors maternity leave and benefits? If so, who monitors? As a mother, do you have any role in monitoring maternity leave and benefits? If so, please describe your role.
6. Can you explain what happens if this policy is not followed? What action (if any) would be taken? By whom?
7. If you had the power to change this policy, what aspects would you change (if any)? Why? How could this policy be improved?
8. Does this policy affect how you and your family care for your child? Why or why not?
9. Does this policy affect how household tasks are managed in your family? Why or why not?
10. Did you continue breastfeeding when you returned to work?
    a. If not, how are you now feeding your child? How did you make this decision?
    b. If yes, how did you manage this? Who supported you and how? How did you make this decision?
</td></tr>
</table>

	11. Did you experience discrimination at your workplace as a result of your pregnancy? If yes, what kind of discrimination? From whom?

Part 2C: "Pros and cons" of maternity leave

1. Now I'd like to talk with you about your perceptions of maternity leave and benefits policies. Let's start with the positives.

    a. In your opinion, what are the "pros" of maternity leave and benefits policies? (probe for: happier workers, less turnover, healthy children) *[Write each response on a separate piece of paper / sticky note.]*
    b. Now I'd like you to rank the pros you mentioned from most to least important. Which of these are most important? Next? *[Repeat this process, until all the paper / sticky notes are organized from most to least important. Write down this sequence or take a photo and wrote a note PHOTO [title]. The time of photo taken, and the title will be used to pair the photo with the IDI transcript.]*
    c.

    [Repeat this process, but for the cons by asking:]

    d. In your opinion, what are the "cons" of maternity leave and benefits policies? (probe for: cost) *[Write each response on a separate piece of paper / sticky note.]*
    e. Now I'd like you to rank the cons you mentioned from most to least important. Which of these are most important? Next? *[Repeat this process, until all the paper / sticky notes are organized from most to least important. Write down this sequence or take a photo and wrote a note PHOTO [title]. The time of photo taken, and the title will be used to pair the photo with the IDI transcript.]*

    f. You said [x] is the biggest pro and you said [x] is the biggest con. In your opinion, does the pro outweigh the con or is the con too great? Why?

Part 3: Communication and Recommendations

1. How would you describe a good Wife? A good Mother?

2. How would you describe a good partner? A good parent?

3. What do fathers usually do around the house?

4. What would mothers like their partners to do more of?

5. Has anyone advised you about raising children?
    a. If yes, who talked to you? What did they say?

6. Who is the best source of information for others like you to learn about child care and feeding?

Closing

Thank you so much for sharing your thoughts with me. I have learned so much about you and life in your community. Before we say goodbye, is there anything else you would like to tell me about the topics we discussed today? *[Record all questions]*

Give participant a chance to say more and then thank the participant again. If she has nothing more to say, thank her and close.

IDI Guide: Partners of the interviewed mothers or Fathers with children < 12 months

Guiding Research Questions for Partners of women with children < 12 months old

- How do families manage work and child feeding and care?
- What are families' experiences receiving maternity leave and benefits?
- What are perceptions of maternity leave and benefits?
- How do families make decisions about child feeding and care?
- What are current perceptions and practices, including barriers and motivations, of early initiation of breastfeeding, and skin-to-skin contact and opportunities to increase?
- What are current perceptions and practices, including barriers and motivations, of exclusive breastfeeding, and opportunities to increase?
- What are current perceptions and practices, including barriers and motivations, of continued breastfeeding, and opportunities and facilitators to lengthen duration?
- What are current perceptions and practices related to feeding young children breastmilk substitutes and milk products?
- What are men's expectations of their roles and responsibilities in child feeding and care?
- Who influences men on these topics, and by which communication channels or social networks?
- What are opportunities to engage men in supporting women's empowerment and participation?

Introduction

[Use this text if there is a delay between the consent process and the interview. Otherwise, thank the participant again for making time to speak with you and use the second paragraph to start the interview.]

Hello! My name is [x] and I come from [x]. Thank you for taking time to talk with me today! I asked to speak with you because I spoke with your partner and I wanted to learn more about your family, but from your perspective.

Our time together may last about 1 hour. I will ask you some questions. I will not say much because I want you to do all the talking; I care about your answers and I want to learn from you. The questions have no right or wrong answers. I only ask that you share what you truly think and feel.

Your answers will represent the thoughts and feelings of hundreds of men just like you, and those men want you to speak for them. Your ideas will help us to strengthen policies and programs to support the health and nutrition of women and children.

[After a self-introduction, say:]
From talking with your partner, I understand you have a child <12 months old. Please tell a little bit about this child (probe for: age, gender).

[Give the participant time to answer, then say:]
I will ask you more questions about [name] in a bit, but first I'd like to show you some images and ask you questions about them. Please feel free to share your own experience if you feel comfortable.

Part 1: Perceptions of Feeding Practices

Part 1A: Photo-based Discussion

[Show a photo of a newborn child and ask:]

- How do families feel about having a newborn child in the household?
- What is important for a child this age? (Probe for: ceremonies; breastfeeding, food/drinks; play; affection; health care)
- What is important for the mother of a child of this age? (Probe for: support to eat more and the right foods – which foods, social support, etc.)
- What concerns do fathers have for children of this age? (Probe for: differences by girls and boys? Illnesses – which illnesses most concern/ what happens?)
- How soon do you think the child was breastfed? Why do you say that?
- Some children take liquids or food first, before breastmilk, right? Why?
- Would partners be willing to help mothers breastfeed the child immediately after birth, and not give other liquids or food first? Why or why not? How could partners help?

[Show photo of the 5-month-old Child and ask:]

- Here is a 5-month old child. What is important for a child this age? (Probe for: ceremonies; breastfeeding, food/drinks; play; affection; health care)
- What is important for the mother of a child of this age? (Probe for: support to eat more and the right foods (which foods), social support, etc.)
- What concerns do partners have for children of this age? (Probe for: differences by girls and boys? Illnesses – which illnesses most concern/ what happens?)
- What is this child drinking or eating? Why do you say that?
- Some children get other food and drinks before 6 months. What is the reason?
- Do some children get only breastmilk until 6 months? Who are these families?
- Would partners be willing to help mothers give breastmilk only to the child until 6 months? Why or why not? How could partners help? (Probe for: wet nurse, bring child to mother's work, mother wait until 6 months to work, other?)
- What age should the child eat semi-solid food? (Probe for: What makes you give children food- signs from child, mother's work situations)
- What are challenges to wait until a child is 6 months to give food and liquids, in addition to breastmilk?
- Would families wait to start food and liquids when the child is 6 months? Why or why not? How?

[Show photos of the 12 and 22-months-old Children and ask:]

- Here are two children. One is 12 and one is 22 months old. What is important for children of these ages? (Probe for: ceremonies; breastfeeding, food/drinks; play; affection; health care)
- For partners, what is good about caring for children of this age? (Probe for: Most enjoyable interactions? Feed child sometimes- why or why not?)
- Do you think the children are still breastfed? Why do you say that?
- Some children stop breastfeeding before the age of 2, right? Why?

- Would partners help mothers continue to breastfeed until 2 years? Why or why not? (Probe for: Advice, taking child to work, other? Who could do this?)

Part 1B: Story

Now I will tell a short story. Please help me to finish the story by answering some questions after each part of the story. Please feel free to share your own experiences, if you feel comfortable.

[Woman name] had a baby! Although she felt tired, she put the baby on her chest, and hoped the baby would take breastmilk. When she said she did not feel the milk coming in, a health worker suggested that she give the baby infant formula while [Woman name] rested.

i. Does this happen here sometimes?
    i. Why or why not?
       (Probe for: confidence of mother, influence of others)
    ii. When does it happen?
       (Probe for: place of delivery, experience of mothers)

j. Do you think the health worker was right to give that advice? If not, what advice should she have given [Woman name] instead?

k. Where else might [Woman name] get advice about breastfeeding?

l. Where do you think [Woman name] should get advice about breastfeeding?

After birth, [Woman name] stayed home from work and gave the baby only breastmilk-- no other food or drinks. Then, after 2 months, [Woman name] returned to work at a shop in a town nearby. Sometimes, when she went out, her mother-in-law cared for the baby and gave the baby infant formula.

h. Does this happen here sometimes?
    i. Why or why not?

i. Some women are able to stay home for 6 months after delivering a baby. How are they able to do that? Why wasn’t [Woman name] able to do this?

j. What would enable [Woman name] to continue breastfeeding even when she returned to work? (probe for: workplace support, family support like bringing the baby to [Woman name] at work)

In town [Woman name] and her partner see billboards and other advertisements for formula milk. They also see commercials on TV that say these milks can make children strong and smart. But the midwife says that breastfeeding is best. [Woman name] feels confused about this.

g. Does this happen here sometimes?
    ii. Why or why not?

h. Do you know about any laws or policies regulating advertising like this? If so, please explain what you know.

i. Did you ever receive information about formula milk? If so, where? From whom?

When the child of [Woman name] was over 1 year, she stopped giving breastmilk. The child eventually stopped asking. The family bought formula milk for the child and gave that instead.

e. Does this happen here sometimes?
    iii. Why do mothers stop breastfeeding?
    iv. When is it more likely to happen?
    v. How do you think *[Woman name]* felt when the child stopped breastfeeding?
    vi. Do some families give formula milk powder or other milk? Why? Where is it purchased? How is it prepared?
f. Could *[Woman name]* find a way to continue breastfeeding until the child reaches 2 years or older?
    vii. Why or why not?
    viii. How could she do this?
g. How could her family support her?
h. Do some mothers here breastfeed the child until 2 years? How is this possible?

Part 1C: Personal experience

I would now like to ask you about you and your family's experience feeding your child.

- Before or after [name] was born, did anyone provide you with information about breastfeeding? If yes, who? What kind of information?
- Who is involved in feeding and caring for your child? *[Ask about each person mentioned and their role]*
- How does child feeding affect the way you and your partner manage household tasks?

[IF the mother is working outside the home, ask:]
- How long after your child's birth did your partner return to work? How did she make the decision to return to work?
- Did you receive any information about maternity leave or benefits prior to the birth of your child? If yes, what kind of information? From whom did you receive this information?
- Did your partner utilize the full duration of maternity leave provided by her employer? Why or why not?
- Did your partner receive any cash and non-cash benefits during her maternity leave? Why or why not? Can you describe the benefits she received?
- As a father, did you receive any paid parental leave after the birth of your child? If so, how long was this leave? Did you receive benefits during that time? If so, what kinds of benefits (cash and non-cash)?
- Did you utilize the full duration of parental leave provided by your employer? Why or why not?
- As a father, do you have any role in monitoring receipt of you or your partner's parental leave and benefits? If so, please describe your role.
- If you had the power to change parental leave policies, what aspects would you change (if any)? Why? How could this policy be improved?
- Does this policy affect how you and your family care for your child? Why or why not?
- Does this policy affect how household tasks are managed in your family? Why or why not?
- Did your partner continue breastfeeding when she returned to work?
    a. If not, how are you now feeding your child? How did you make this decision?
    b. If yes, how did you manage this? Who supported you and how? How did you make this decision?
- Did your partner experience discrimination at your workplace as a result of her pregnancy? If yes, what kind of discrimination? From whom?

Part 1D: "Pros" and "cons" of maternity leave

Now I'd like to talk with you about your perceptions of maternity leave and benefits policies. Let's start with the positives.

g. In your opinion, what are the "pros" of maternity leave and benefits policies? (probe for: happier workers, less turnover, healthy children) *[Write each response on a separate piece of paper / sticky note.]*
h. Now I'd like you to rank the pros you mentioned from most to least important. Which of these are most important? Next? *[Repeat this process, until all the paper / sticky notes are organized from most to least important. Write down this sequence or take a photo and wrote a note PHOTO [title]. The time of photo taken, and the title will be used to pair the photo with the IDI transcript.]*

[Repeat this process, but for the cons by asking:]

i. In your opinion, what are the "cons" of maternity leave and benefits policies? (probe for: cost) *[Write each response on a separate piece of paper / sticky note.]*
j. Now I'd like you to rank the cons you mentioned from most to least important. Which of these are most important? Next? *[Repeat this process, until all the paper / sticky notes are organized from most to least important. Write down this sequence or take a photo and wrote a note PHOTO [title]. The time of photo taken, and the title will be used to pair the photo with the IDI transcript.]*

k. You said [x] is the biggest pro and you said [x] is the biggest con. In your opinion, does the pro outweigh the con or is the con too great? Why?

Part 3: Communication and Recommendations

Now I'd like to turn to my final set of questions for you.

- Whom do you admire as a parent (or whom do you want to be like)? Can you describe them?
  - What do you want for your sons in the future? Daughters?
  - What should your children say about you, as their parent, in the future?
- What do partners usually do around the house?
- What do you think mothers would like partners to do more of?
- Has anyone advised you about raising children?
  - If yes, who talked to you? What did they say?
- Who is the best source of information for others like you to learn about child care and feeding?

Closing

Thank you so much for sharing your thoughts with me. I have learned so much about you and life in your community. Before we say goodbye, is there anything else you would like to tell me about the topics we discussed today? *[Record all questions]*

Give participant a chance to say more and then thank the participant again. If s/he has nothing more to say, thank her/him and close.
