# Supplementary material for: Assessing the Effectiveness of Policies Relating to Breastfeeding Promotion, Protection, and Support in Southeast Asia: Protocol for a Mixed Methods Study
Source: JMIR Res Protoc. 2020 Sep 21;9(9):e21286. doi: 10.2196/21286 (PMC7536596; doi:10.2196/21286)
Supplement: Multimedia Appendix 3 [file resprot_v9i9e21286_app3.pdf]

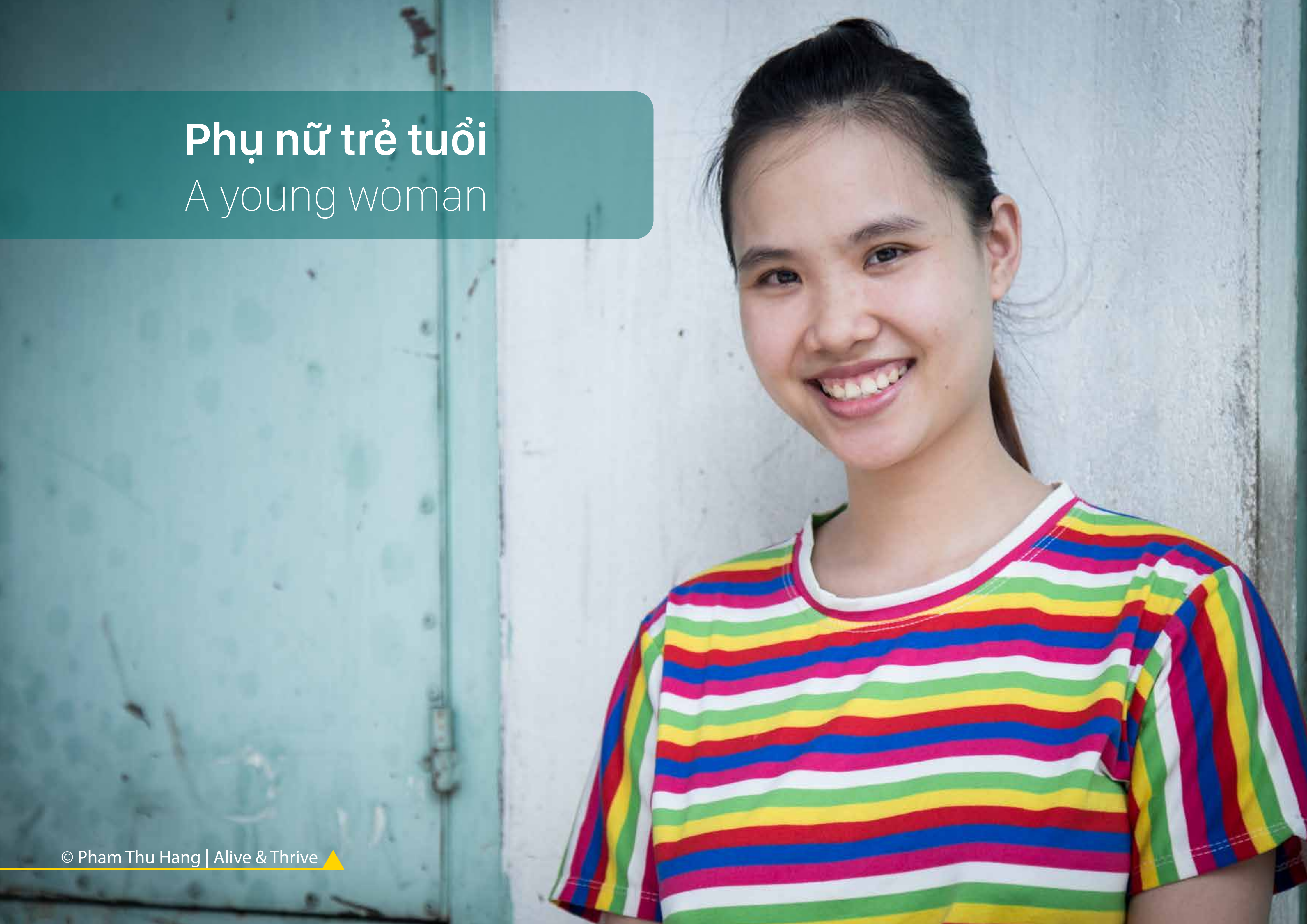

Phụ nữ trẻ tuổi

A young woman

© Pham Thu Hang | Alive & Thrive

Phụ nữ truyền thống
A traditional woman

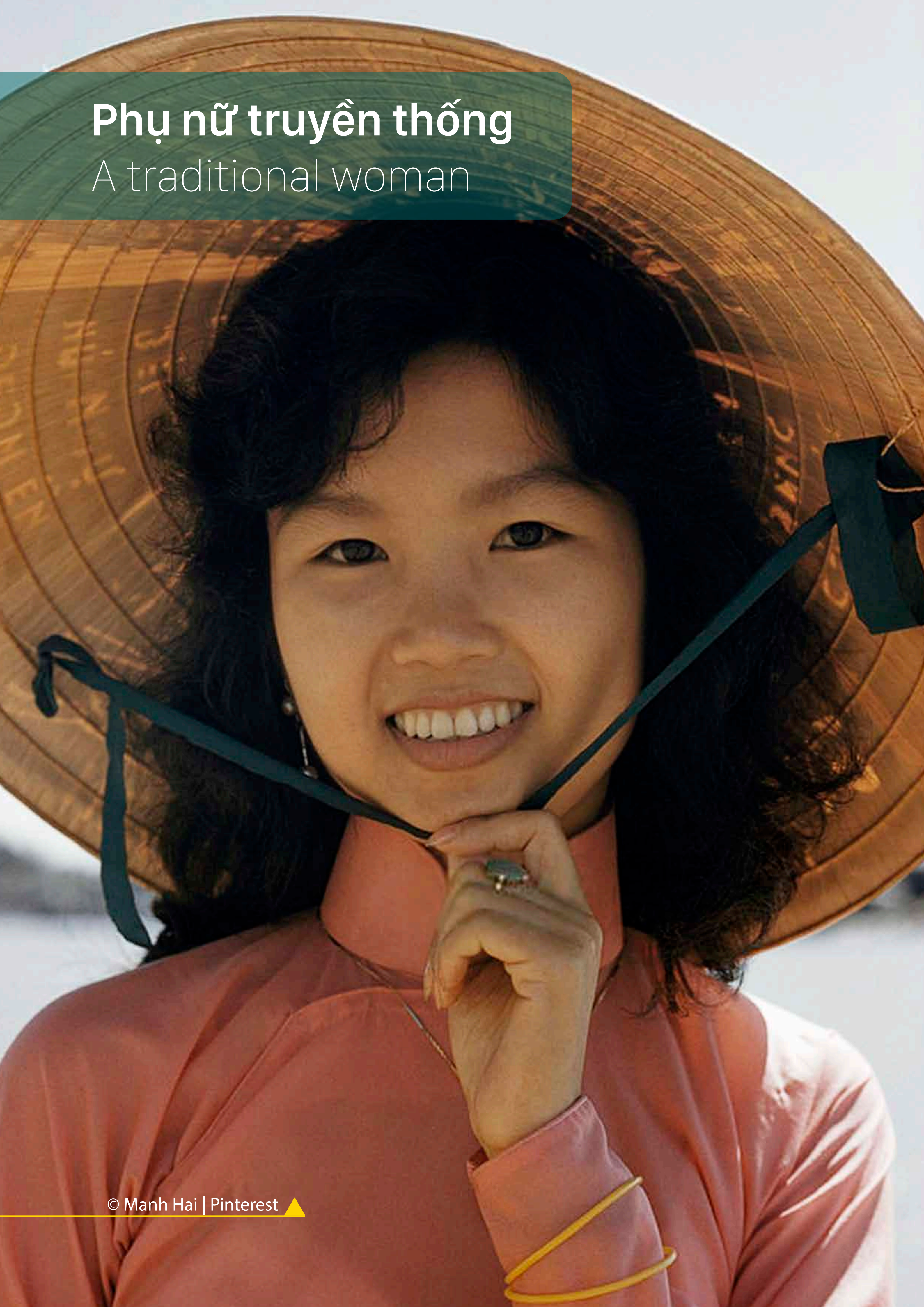

© Manh Hai | Pinterest

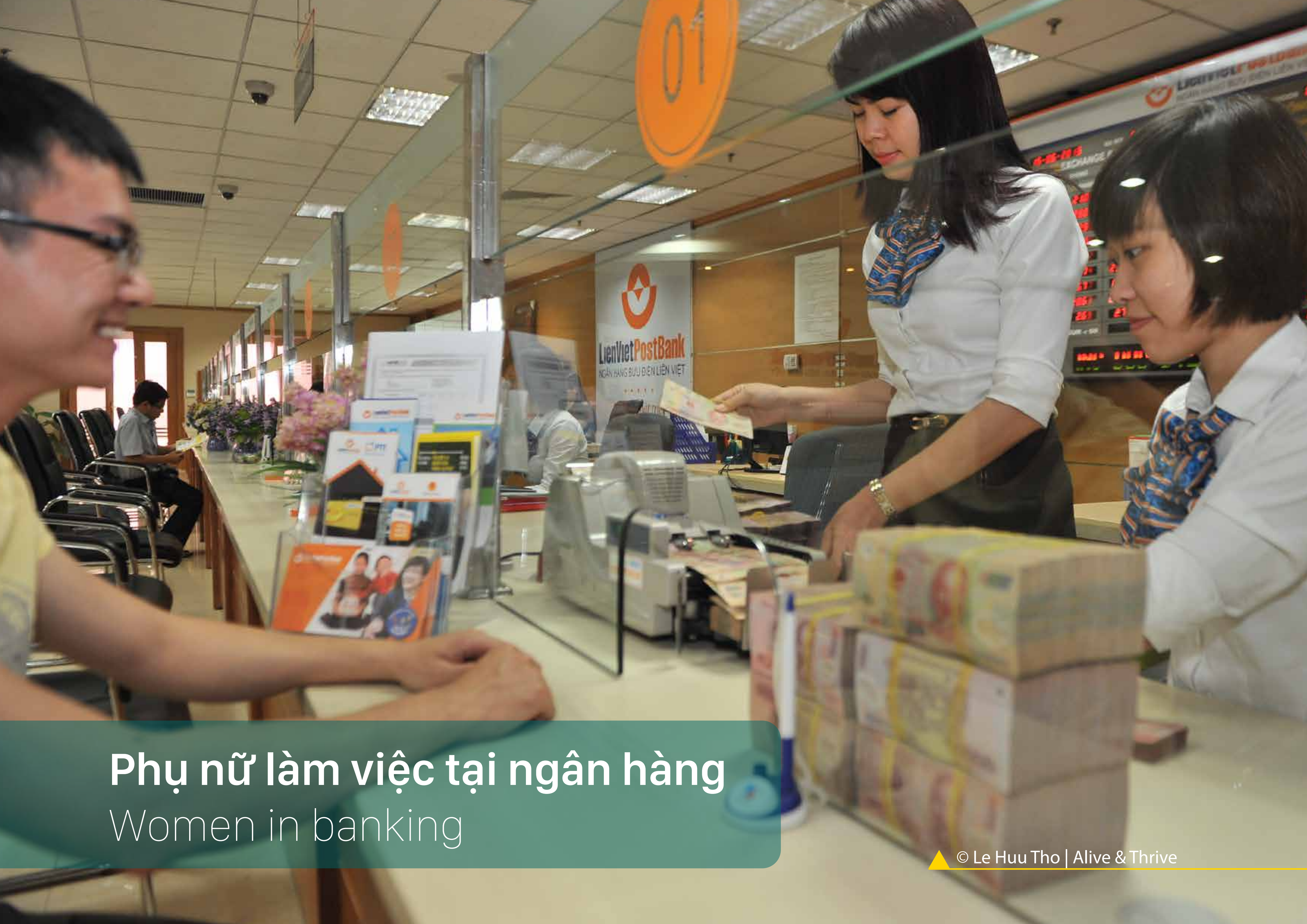

Phụ nữ làm việc tại ngân hàng

Women in banking

© Le Huu Tho | Alive & Thrive

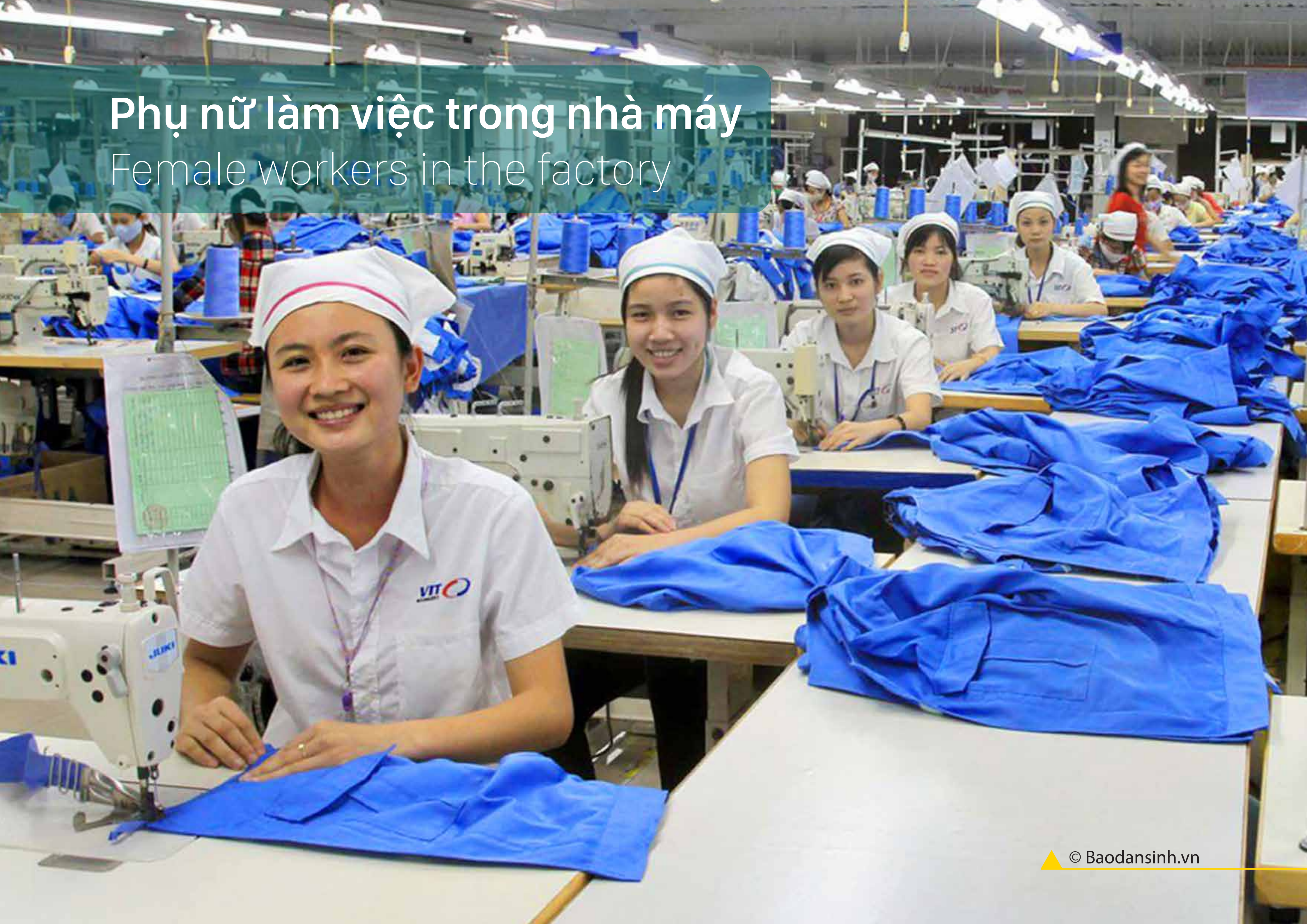

Phụ nữ làm việc trong nhà máy
Female workers in the factory

© Baodansinh.vn

Phụ nữ làm nông
A woman in agriculture

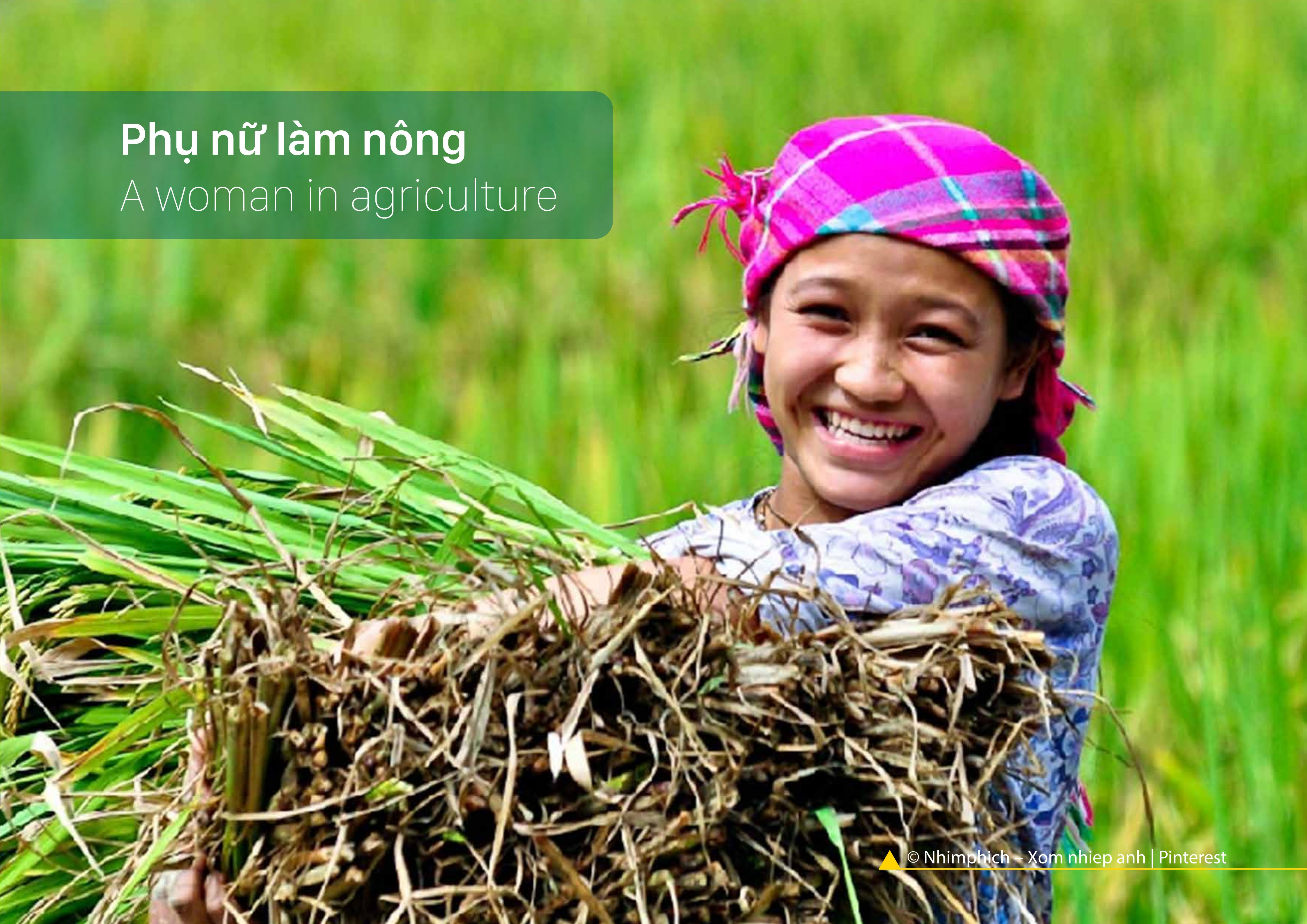

© Nhimphich – Xom nhiep anh | Pinterest

Phụ nữ công sở
Women in the office

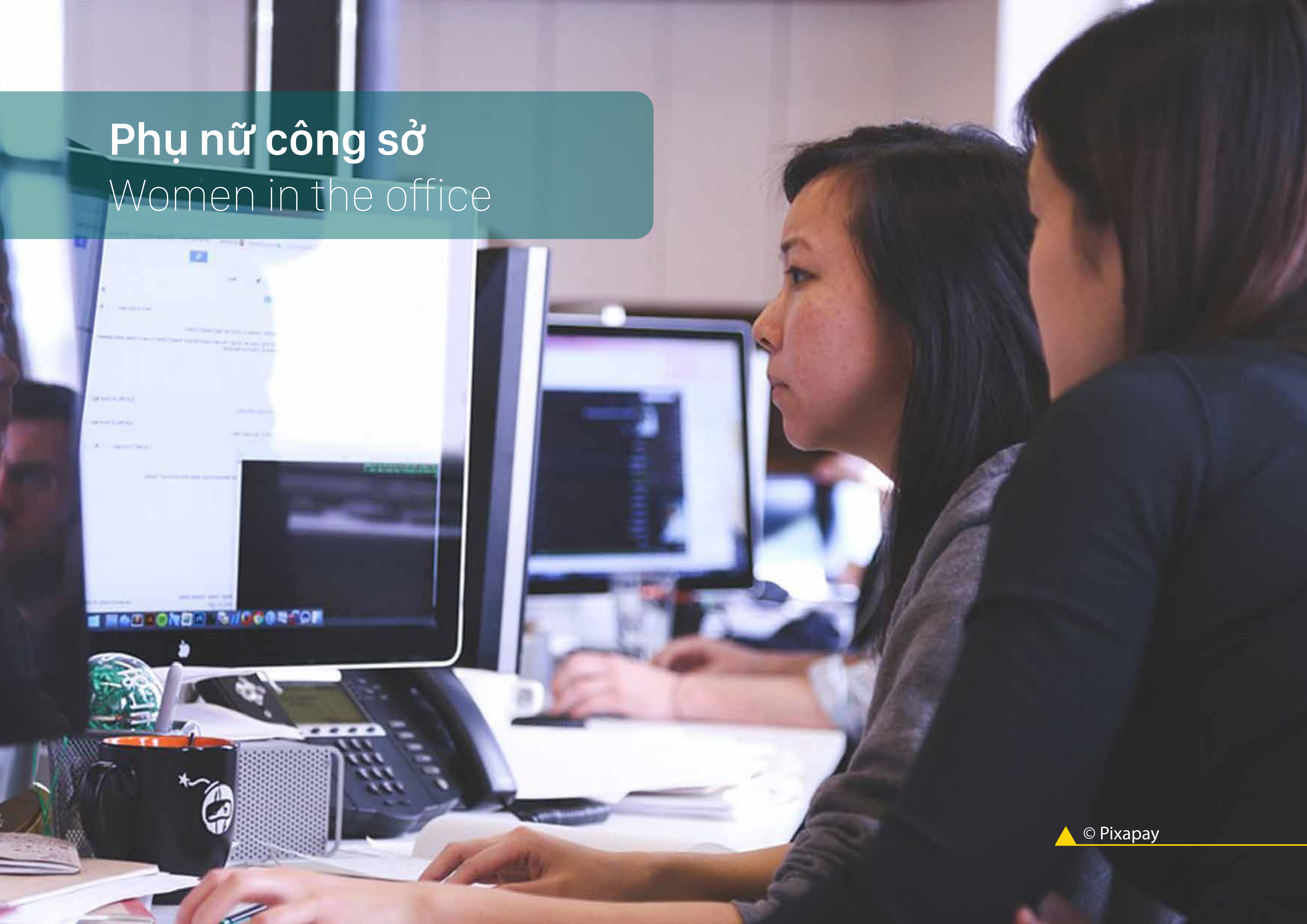

© Pixapay

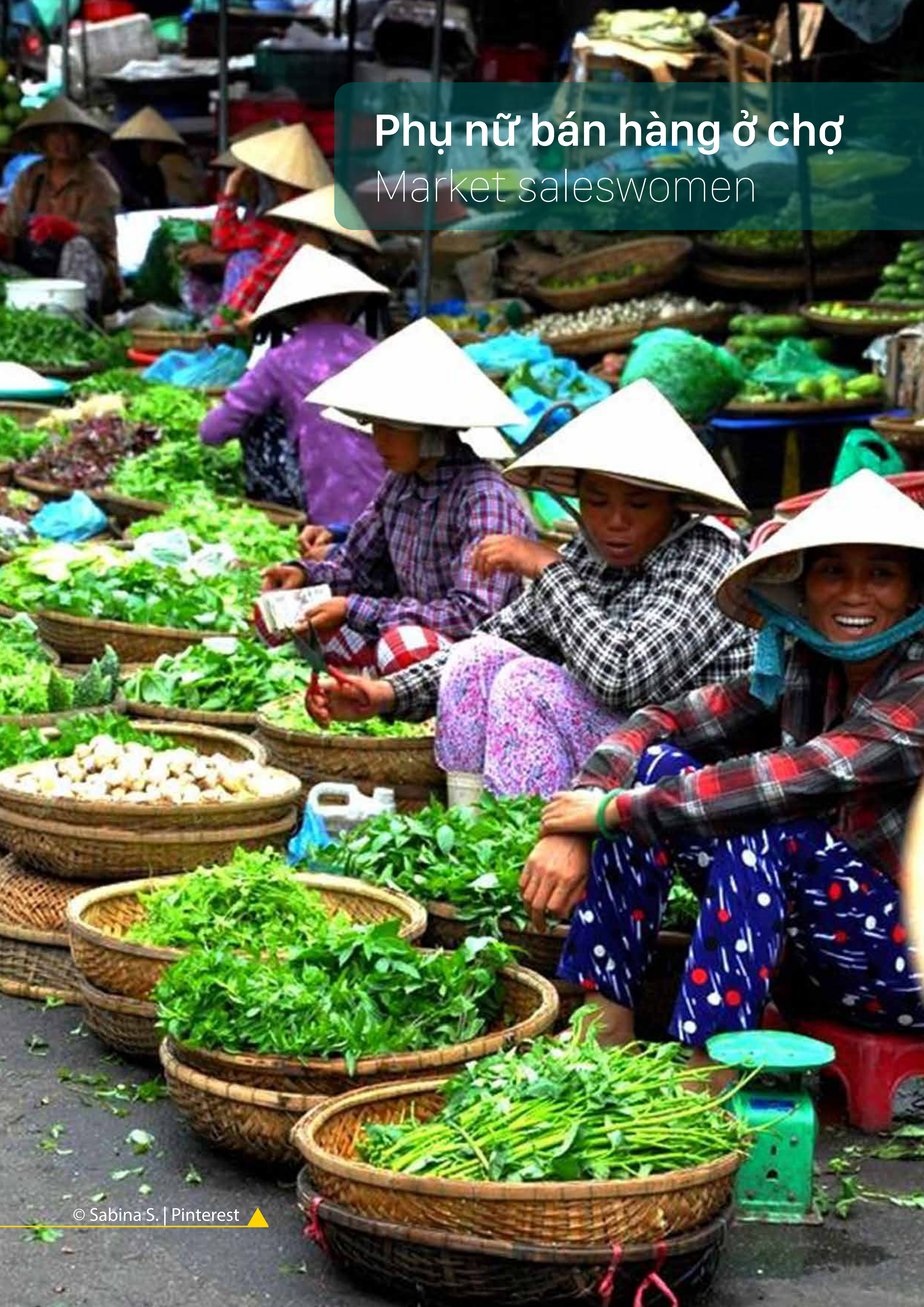

Phụ nữ bán hàng ở chợ
Market saleswomen

© Sabina S. | Pinterest

Phụ nữ mang thai
Pregnant women

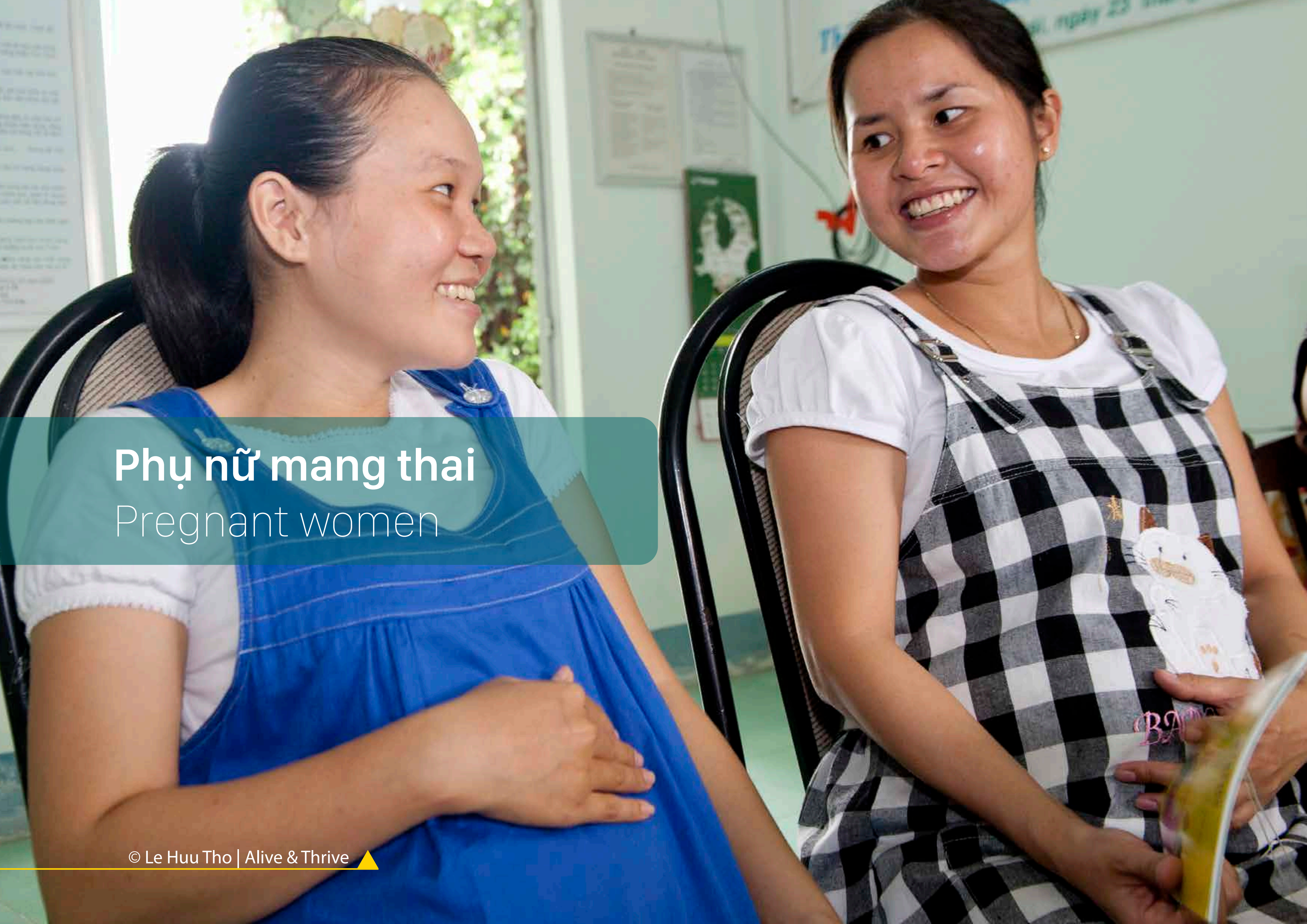

© Le Huu Tho | Alive & Thrive

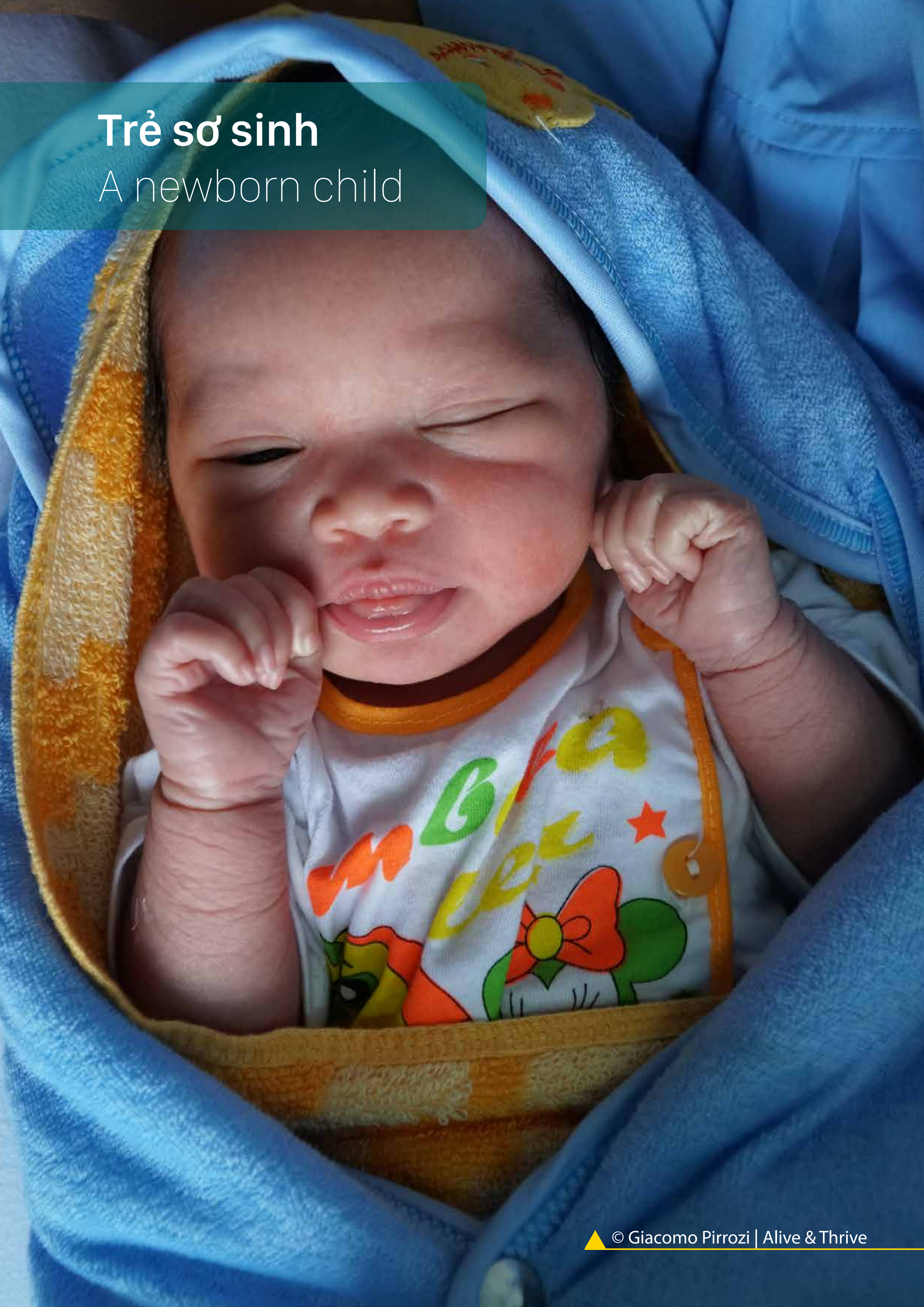

Trẻ sơ sinh
A newborn child

© Giacomo Pirrozi | Alive & Thrive

Trẻ 5 tháng tuổi
A child aged five months

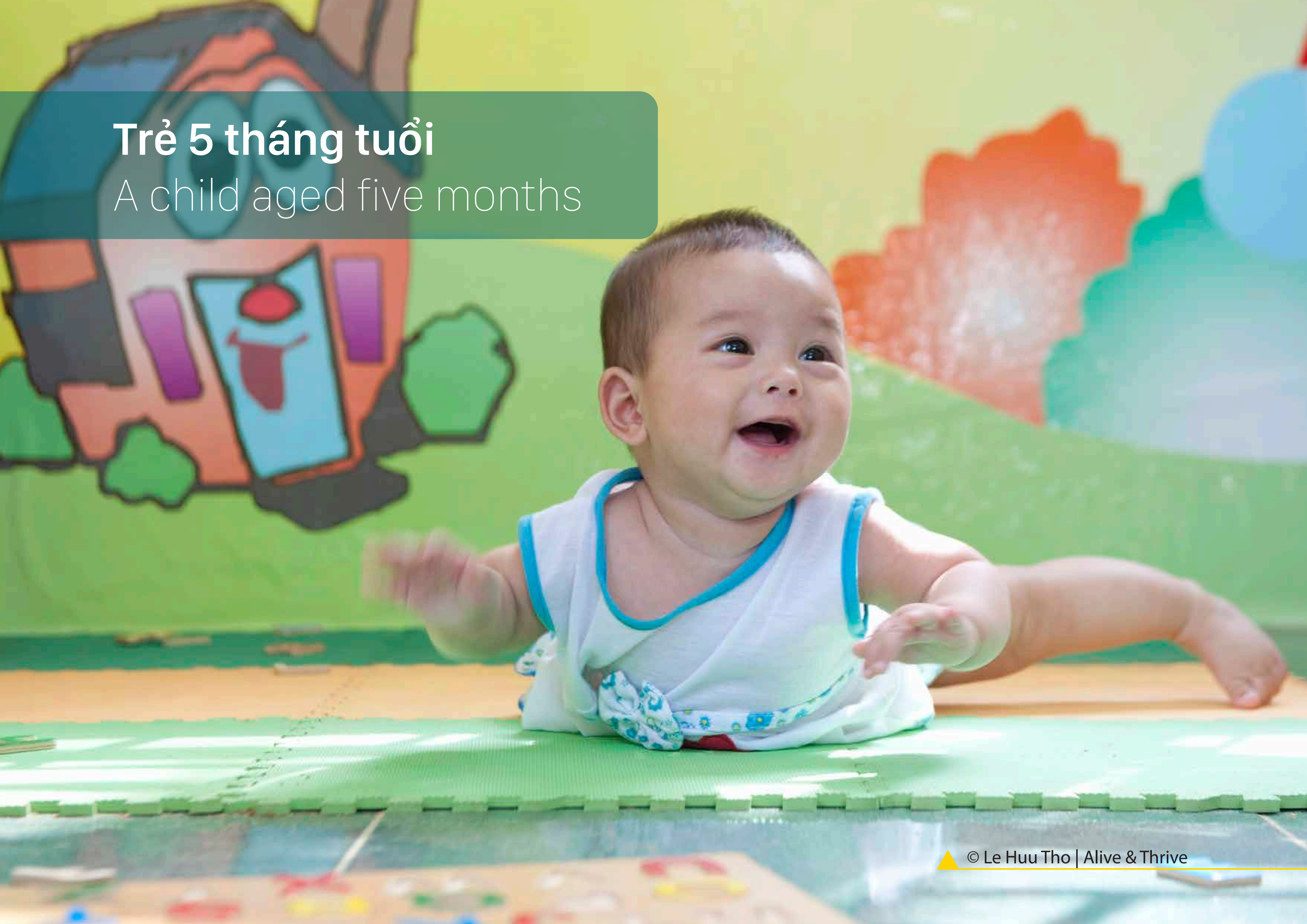

© Le Huu Tho | Alive & Thrive

Trẻ 12 tháng tuổi
A child aged 12 months

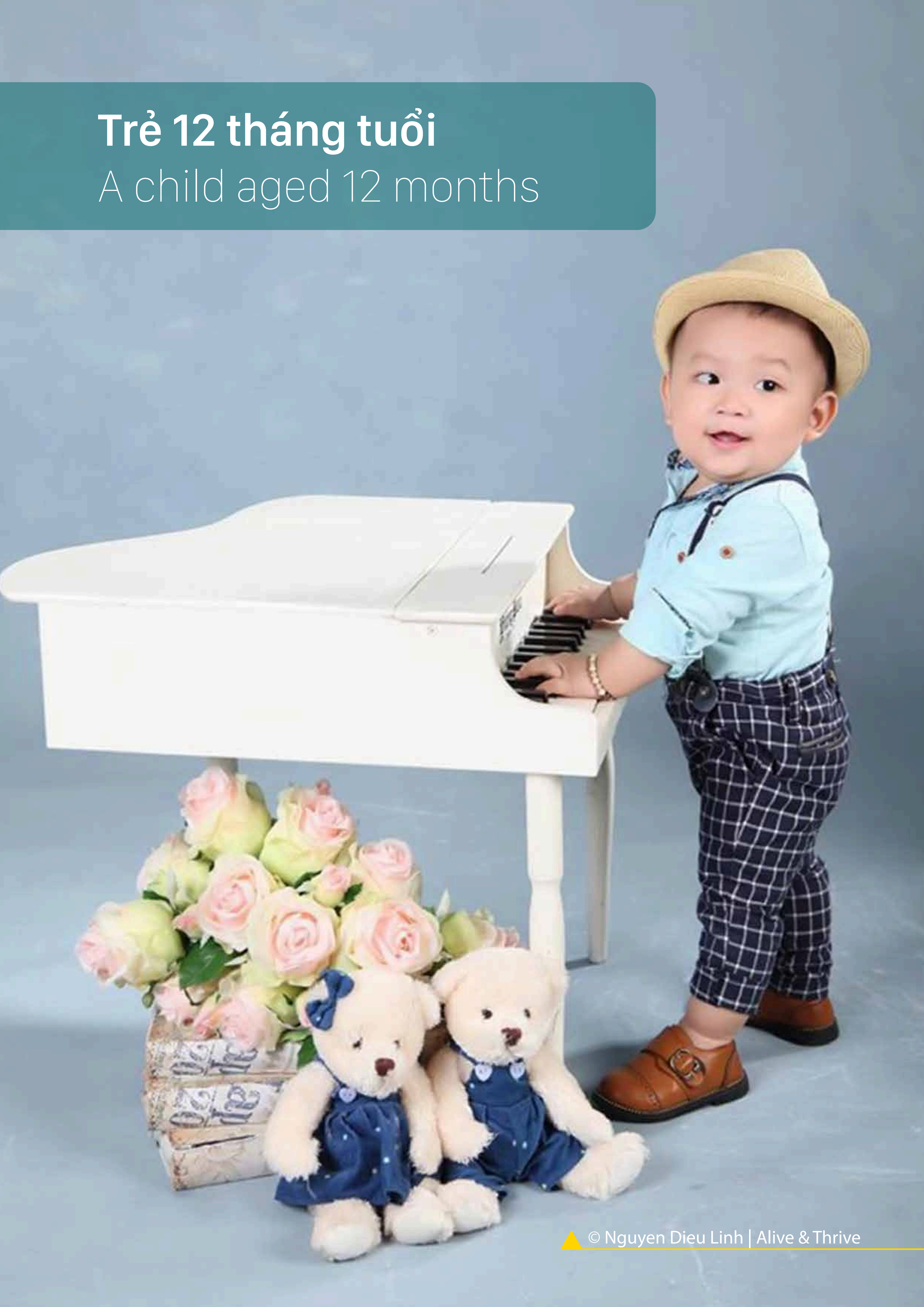

© Nguyen Dieu Linh | Alive & Thrive

Trẻ 22 tháng tuổi
A child aged 22 months

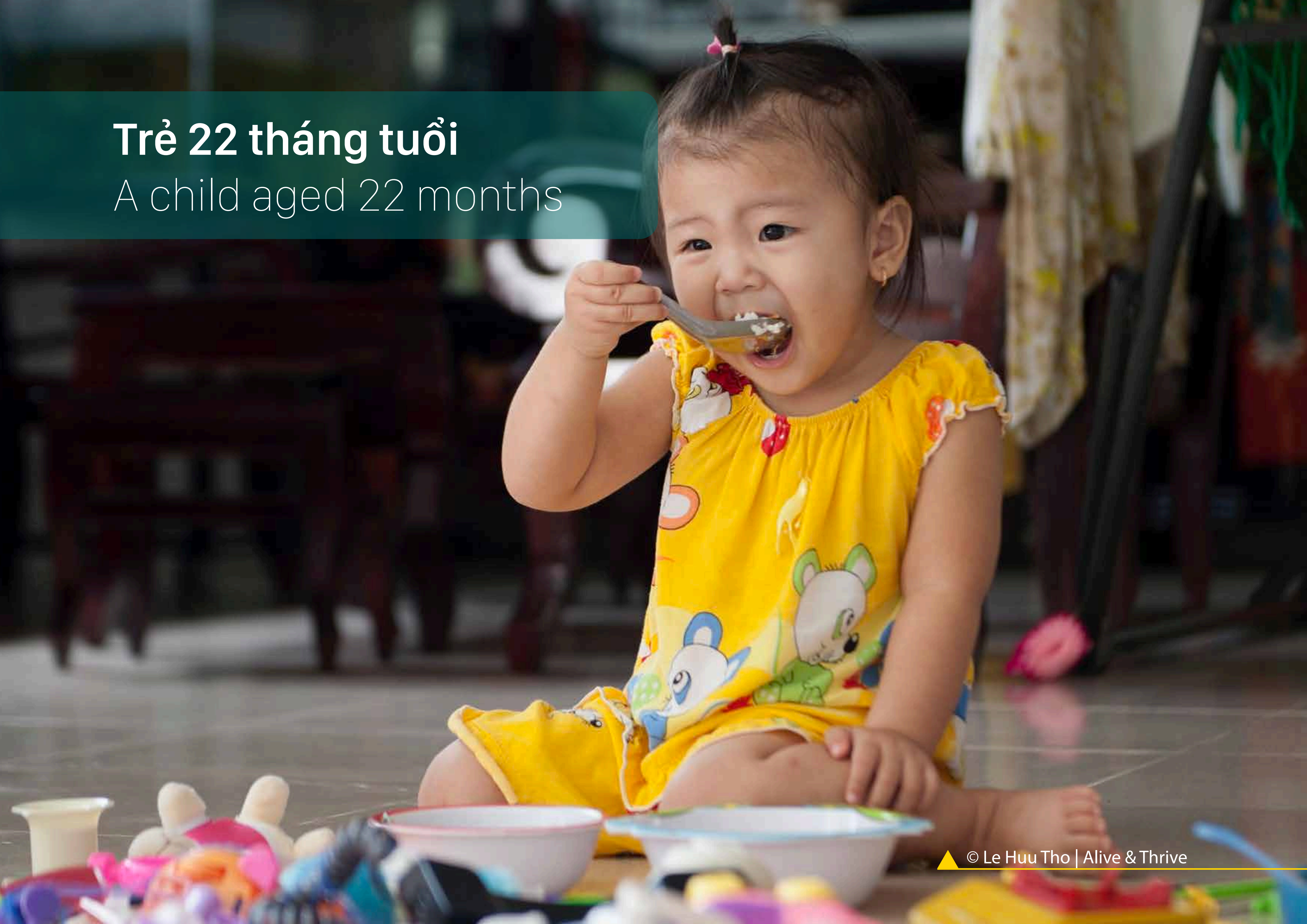

© Le Huu Tho | Alive & Thrive

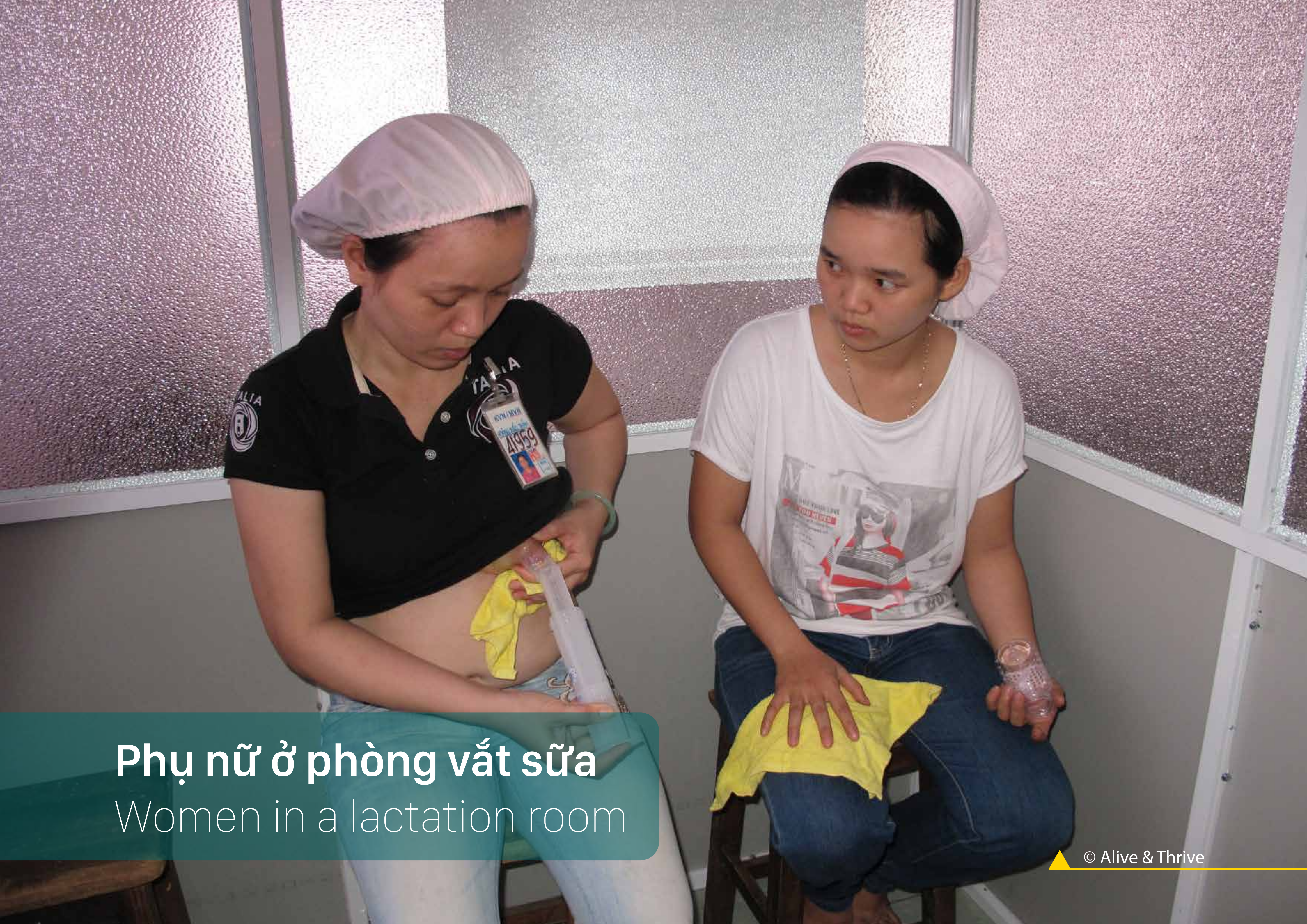

Phụ nữ ở phòng vắt sữa

Women in a lactation room

© Alive & Thrive
