# Supplementary material for: Assessing the Effectiveness of Policies Relating to Breastfeeding Promotion, Protection, and Support in Southeast Asia: Protocol for a Mixed Methods Study
Source: JMIR Res Protoc. 2020 Sep 21;9(9):e21286. doi: 10.2196/21286 (PMC7536596; doi:10.2196/21286)
Supplement: Multimedia Appendix 4 [file resprot_v9i9e21286_app4.pdf]

APPENDIX 4. QUESTIONNAIRE FOR PREGNANT WOMEN

1. BACKGROUND AND CHARACTERISTICS

No	Question	Code	Variable name
1	Interviewer's name	Select name	Int
2	Province/ city that the interview is being conducted in:	1= HCM city<br>2= Binh Duong<br>3= Bac Ninh	Prov
3	PSU code	Enter code 1-30	Psu
4	What is your name/code of mother?	....	Mname
5	Enter women's code	***2 digits***	Mcode
6	Unique ID (automatically generated)	***Provivnce.PSU.Women.Type***<br>***(check with code on the spread sheet)***	Id
7	What is your ethnicity?	Kinh .... 1<br>Other (specify)………………………….. 77	Ethnic
8	What is your year of birth?	____________	Myob
9	What is your marital status?	Unmarried .... 1<br>Married .... 2<br>Widowed/Divorced/Separated .... 3<br>Other (specify)....................................... 77	Marital
10	Are you living with your husband/partner?	1. Yes<br>0. No	plivewt
11	What is the highest level of education you have **<u>completed</u>**?	Never attended school ....0<br>Primary school ....1<br>Junior secondary school ....2<br>Secondary school ....3<br>Diploma ....4<br>Bachelors/Masters/higher……………...5	mEdu
12	How many biological children do you have?	☐ children	bchild
13	How many biological children under 5 years of age do you have?	☐ children	Child
14	How many biological children under 12 months do you have?	☐ children	Under12
18	What is your current <u>main</u> occupation?	Farmer (planting, feeding animals, farming)/fisherfolk .... 1<br>Green-collar: worker, technician, janitor .. 2<br>White-collar: educated staff, office clerk, doctor, teacher, enterprise owner .... 3<br>Small trader/ self-employed / small self-owned business/services (tailor/hairdresser/builder)/freelancer........ 4<br>Unemployed / Homemaker/ student… ... 5<br>Other (specify)…………………………….77	mjob
19	Are you contributing to the Social Security Fund?	Yes .... 1<br>No 0	insur

	This is the money taken from your paycheck. Then the fund provides some money to you during sickness, maternity, labor accident, old age…	Don't know .... 98<br>No Response 99	
21	How long after giving birth do you plan to go back to work?	……….. month(s)……….. days	Backdate
27	What is your husband/partners' current <u>main</u> occupation?	Farmer (planting, feeding animals, farming)/fisherfolk .... 1<br>Green-collar: worker, technician, janitor .. 2<br>White-collar: educated staff, office clerk, doctor, teacher, enterprise owner .... 3<br>Small trader/ self-employed / small self-owned business/services (tailor/hairdresser/builder)/freelancer........ 4<br>Unemployed / Homemaker/ student… 5<br>Other (specify)…………………………….77	pjob
28	When applying for a job, have you ever been required to **have a pregnancy test** or **doctor's diagnostic certificate** to confirm that you are not pregnant?	Yes .... 1<br>No... 0<br>Have not applied for a job 2	pretest
29	When applying for a job, have you ever **been asked about your plans to have a baby** or **your marital status**?	Yes .... 1<br>No... 0<br>Have not applied for a job 2	famask

2. PRENATAL CARE

Now I will ask you some questions about your pregnancy, childbirth and breastfeeding

No	Question	Code	Variable name
1	What was the first day of your last menstrual period?	__ __ / __ _ / _ _ _ _<br>Don't know………….98 → **Move on Q2**	lmp
1b	Current pregnancy weeks (automatically generated)	__ __ pregnancy weeks → **Move on Q3**	Gestage1
2	As of today, what is your baby's gestational age?	__ __ pregnancy weeks	Gestage2
3	Did you receive antenatal care or an ultrasound during this pregnancy?	Yes .... 1<br>No …… 0 → **Move on Q11**	Anc
4	Where did you go for antenatal care or ultrasound?<br><br>***Multiple responses possible***	Public hospital (provincial and central levels) .... 1<br>Public polyclinic, district health center .. 2<br>Private/International hospital/clinic .... 3<br>Commune health center......................... 4<br>At home…………………………………5<br>Other (specify)…………………………..77	ancplace
5	How many times did you receive antenatal care during this pregnancy that included the following?<br><br>*(ANC: Lying on a bed or obstetric examination table, health worker measuring blood pressure, waist circumference; checking the uterus, fetal heart rate, the appendages and asking the health situation. In some cases, having more tests)*	Number of times................................ __ __<br>Don't know... 98	Anctime

	Probe *to identify the number of times antenatal care was received. If a range is given, record the minimum number of times antenatal care received.*<br>(Note: If you get both ANC and ultrasound in one visit, count 1)		
6	Of the <mentioned the number in question 5> times you received antenatal care, **how many times was it paid mainly by <read each option>?**<br><br>***Multiple responses possible***<br>***Probe:*** Anyone else?<br>*(Note: the add-up total must equal to the total in Q4)*	Health insurance …… 1 __ __ times<br>Company/private health insurance 3 __ __ times<br>Out of pocket (e.g., Myself)……2 __ __ times<br>Other (specify)…. 77 __ __ times	Ancpay1<br>Ancpay2<br>Ancpay3<br>Ancpay4
7	To go for antenatal care, how many times did you **<read each option>**?	Take a break during working hours, with salary __ __ times<br>Take a break during working hours, without salary __ __ times<br>Have checkup out of working hours: ……………… __ __ times	Offpay<br>Notpay<br>offwork
8	What is the total number of times you received ultrasound ONLY during your pregnancy?	__ __ times	ulttot
9	During any of your antenatal care visits at a health facility, did a representative from a milk company <u>approach</u> you to give you information or advice about milk formula, bottles or pacifiers?	Yes……………………………………….1<br>No 0	Prohosp
10	During any of your antenatal care visits at a health facility, did a representative from a milk company <u>approach</u> you to ask for your personal information such as your address, phone number, email address, Facebook, Zalo…?	Yes……………………………………….1<br>No 0	Prohosp2
11	During this pregnancy, did you receive any advice about breastfeeding from anyone?<br>***Multiple responses possible***	Health worker from a health facility....... 1<br>Hamlet health worker............................ 2<br>Nutrition collaborator............................ 3<br>Women Union staff.............................. 4<br>Husband .... 5<br>Mother/Mother in law .... 6<br>Other Family members.......................... 7<br>Neighbors/ Friends/co-workers .... 8<br>Other (*specify*) .... 77<br>No one………………..…………………9	Ancadv
12	During this pregnancy, did you receive any advice about breastfeeding from **any other sources**?<br><br>***Multiple responses possible***	Television……………………………...1<br>Internet (social media, search engine [e.g., Google])………….2<br>Loudspeaker/Radio……………………….3<br>Books/newspapers/magazines/leaflets…...4<br>Advertisement boards, posters……………5<br>Prenatal courses…………………………..6<br>Other events (specify)……………………77<br>Nowhere 9	Ancinfo

13	During this pregnancy, did you drink milk for pregnant women?	1. Yes<br>0. No → **Move on Q14**	pregmilk
13b	Did you drink milk for pregnant women yesterday?	1. Yes<br>0. No	yesterd
14	During this pregnancy, did you take iron folate or micronutrient supplement?	1. Yes<br>0. No	Suppl
15	Where do you plan to give birth to this coming child?	Public hospital (provincial and central levels) .... 1<br>Public polyclinic, district health center .. 2<br>Private/International hospital/clinic .... 3<br>Commune health center......................... 4<br>At home…………………………………5<br>Other (specify)…………………………..77	Bplace
16	Do you plan to give birth vaginally or by cesarean delivery?	Vaginal delivery……………………………1<br>Cesarean delivery .... 0<br>Don't know (depending on health situation and prescription of the doctor) …………98	cesarean

3. DETERMINANTS

3.1. (Intention)

Now I want to ask you about how you plan to nurture the baby that you are going to birth. Please tell me whether you strongly disagree, disagree, disagree somewhat, agree somewhat, agree or strongly agree with each of the following statements.

If the answer is "don't know," code as 98.

1	2	3	4	5	6
**Strongly disagree**	**Disagree**	**Somewhat Disagree**	**Somewhat Agree**	**Agree**	**Strongly agree**

No	**Question**<br>***Please provide your opinion on the following statements:***<br>***('I' here means talking about you and not talking about the person interviewing you)***	Code
1	I will, or I will continue drinking milk for pregnant women	Plan1
2	For the coming baby, I will breastfeed him/her within 1 hour after giving birth	Plan2
3	For the coming baby, I will not give him/her anything other than breastmilk in the first 3 days after birth	Plan3
4	For the coming baby, I will not give him/her any infant formula for the first 6 months.	Plan4

3.2. **(Knowledge of breastfeeding)** Now I would like to ask your opinion about infant feeding.

No	Question	Code	
1	How long after birth should a newborn start breastfeeding?<br><br>If the mother answers "immediately", interviewers ask the mother again about the exact time and record the appropriate time.<br>*If less than 1 hour, circle 1.*<br>*If less than 24 hours, circle 2 and record hours.*	Within 1 hour .... 1<br>Number of hours .... 2<br>Number of days .... 3<br>[ ][ ] Hours [ ][ ] Days<br><br>Don't know 98	keibf

	If more than 24 hours, circle 3 and record days.		
2	If a mother thinks her **4-month-old** infant is not getting enough breastmilk, what should she do?<br><br>***Multiple responses possible.***<br><br>***Probe once:*** Anything else?	Breastfeed more often/more frequently .... 1<br>Give infant formula .... 2<br>Give infant other liquids/foods (e.g. water/fruits juice /Rice porridge/ rice flour) .... 3<br>Mother needs to drink more water .... 4<br>Mother needs to eat more food .... 5<br>Mother needs to eat special food .... 6<br>Consult with health care workers (doctor, nurse, midwife, nutrition collaborator/village health worker) .... 7<br>Seek opinions from social media networks .... 6<br>Other (Specify): .... 77<br>Don't know 98	klackm
3	Until what month should a mother give her infant **<u>ONLY</u>** breastmilk and **<u>NO</u>** other food, water or infant formula?	☐☐ Months<br><br>Don't know……………………………..……........98<br>No response…………………………………….. 99	kebf
4	Until what month should a mother continue to breastfeed?<br><br>***Write down the age as months.***	☐☐ Months<br>Don't know ....98<br>No response...99	kcbf

3.3. (Beliefs)

I would like to ask your opinion about some other feeding practices. Please tell me whether you strongly disagree, disagree, disagree somewhat, agree somewhat, agree or strongly agree with each of the following statements.

If the answer is "don't know," code as 98.

1	2	3	4	5	6
**Strongly disagree**	**Disagree**	**Somewhat Disagree**	**Somewhat Agree**	**Agree**	**Strongly agree**

**No**	**Question**	**Variable name**
	Please tell me your opinion about the following statements.<br>***"I" in all of the following statements refers to the respondent and not the interviewer.***	
1	If I **breastfeed** my infant **within 1 hour after giving birth**, it'll be good for my child's health	Belief1
2	If I am breastfeeding, but **<u>DO NOT</u>** give my newborn infant formula during the first 24 hours after birth, s/he will be hungry.	Belief2
3	If I feed my infant **<u>ONLY</u>** breastmilk and **<u>NO</u>** other food, water or infant formula, until s/he completes 6 months, I am giving my infant all the nutrients s/he needs to be healthy.	Belief3
4	If I feed my infant a combination of breastmilk and infant formula until s/he completes 6 months, I am giving him/her the **<u>BEST</u>** possible nutrition.	Belief4
5	After I start to give infant formula to my infant, my body will produce less breastmilk.	Belief5
6	If I continue breastfeeding until my infant completes 2 years, it is good for my infant's health.	Belief6
7	If I drink milk for pregnant women, my newborn will be smart and healthy.	Belief7
8	I would feel uncomfortable to breastfeed my infant in a public place.	Belief8

3.4. (Social Norms)

I would like to ask your opinion about some social norms of other feeding practices. Please tell me whether you **strongly disagree, disagree, disagree somewhat, agree somewhat, agree or strongly agree** with each of the following statements.

If the answer is "don't know," code as 98.

1	2	3	4	5	6
**Strongly disagree**	**Disagree**	**Somewhat Disagree**	**Somewhat Agree**	**Agree**	**Strongly agree**

No	Question	Variable name
1	**Most people who are important to me (e.g. family members, friends…)** think that an infant needs formula milk in the first week after birth.	Sn1
2	Most people who are important to me (e.g. family members, friends…) would approve of my giving my baby infant formula before she/he reaches 6 months of age.	Sn2
3	Most people who are important to me (e.g. family members, friends…) would approve of me breastfeeding my baby in public places.	Sn3
4	**Most of the pregnant women I know** consume(d) milk for pregnant women.	Sn4
5	**Most of the women with an infant I know** fed their infant breastmilk within 1 hour after vaginal delivery.	Sn5
6	Most of the women with an infant I know fed their infant breastmilk within 1 hour after caesarean delivery.	Sn6
7	Most of the women with an infant I know fed their infant formula milk in the 1 first week of the infant's life.	Sn7
8	Most of the women with an infant I know fed their infant only breastmilk, and no other food, water, or infant formula for the first 6 months of the infant's life.	Sn8
9	Most of the women with an infant I know breastfed their infant in a public place.	Sn9
10	Most of the women with an infant I know feed their infant milk formula when they take them out of the house and need to feed them.	Sn10

3.5. (Self-Efficacy)

I would like to ask your opinion about some other feeding practices. Please tell me whether you are **very unconfident, unconfident, somewhat unconfident, somewhat confident, confident, or very confident** in response to the statement.

As you read the responses, point to each box.

If the respondent does not know, code 98.

1	2	3	4	5	6
**Very unconfident**	**Unconfident**	**Somewhat unconfident**	**Somewhat confident**	**Confident**	**Very confident**

No	Question	Code
1	My body can produce enough colostrum to feed my newborn within one hour after birth.	Conf1
2	My breastmilk is of good enough quality to nourish my infant so that my infant does not need any other food, water, or infant formula until s/he has completed 6 months.	Conf2
3	If I go back to work before my infant is six months old, I will have to start feeding him infant formula or semi-solid/solid foods.	Conf3
4	I can continue to breastfeed my infant until she/he **reaches 24 months of age**.	Conf4
5	I can breastfeed in a public place if my infant is hungry.	Conf5
7	If money were not an issue, I would stay home to feed my infant during his/her first 6 months.	Conf7

8	If money were not an issue and I could stay at home for the first 6 months, I would only breastfeed my infant and wouldn't feed him/her with any other food or drink or formula milk during the first 6 months.	Conf8
9	When I look at the formula label in a store or supermarket, I will recognize which age range the formula is for.	Conf9
10	If a representative from a milk company recommends a new infant formula, I will buy it for my infant.	Conf10
11	If a health worker recommends a new infant formula, I will buy it for my infant.	Conf11
12	If a friend of mine recommends a new infant formula, I will buy it for my infant.	Conf12
13	If members of my social network group(s) recommend a new infant formula, I will buy it for my infant.	Conf13

4. MEDIA EXPOSURE AND SOCIAL GROUP AND EVENT PARTICIPATION

Now I would like to ask you some questions about media exposure

No	Question	Code	Variable name
1	During last 30 days, did you see any information **on breastfeeding** on television, screens at a health care center, supermarkets, elevators or outdoors?	Yes ....1<br>No ………………0 → **Move on Q3**<br>Don't know/don't remember 98	Media1
2	In the past 30 days, how often did you see the information on breastfeeding from any of the above media? Was it…<br>***Use Show card***	Daily (7 days/week) .... 1<br>Several times a week (2-6 days/week) .... 2<br>About once a week .... 3<br>Less than once a week (≤ 3 days/month) .... 4<br>Don't know/don't remember 98	Media2
3	**Now, I am asking about milk formula advertisements**. During the last 30 days, did you see any information on **milk formula** on television, screens at supermarkets, elevators or outdoors?	Yes .... 1<br>No …………….. 0 → **Move on Q5**<br>Don't know/don't remember 98	Media3
4	In the past 30 days, how often did you see information on **milk formula** on television, screens at supermarkets, elevators or outdoors? Was it…<br>***Use show card***	Daily (7 days/week) .... 1<br>Several times a week (2-6 days/week) .... 2<br>About once a week .... 3<br>Less than once a week (≤ 3 days/month) .... 4<br>Don't know/don't remember 98	Media4
5	In the past 6 months, have you been a member of any **online social group** for mothers and other caregivers of infants and young children, such as infant clubs or parenting groups?	Yes ....1<br>No ....0<br>Don't know/don't remember 98	Media5
6	In the past 6 months, have you participated in any **online events or activities** hosted for mothers and other caregivers of infants and young children, such as photo contests or promotional sales on e-commerce platforms?	Yes ....1<br>No ....0<br>Don't know/don't remember 98	Media6
7	In the past 6 months, have you been a member of any **in person social groups** for mothers and other caregivers of	Yes ....1<br>No ....0<br>Don't know/don't remember 98	Media7

	infants and young children, such as infant club or parenting group?		
8	In the past 6 months, have you attended **any classes** on parenting or infant and young child feeding?	Yes ....1<br>No ....0<br>Don't know/don't remember 98	Media8
9	In the past 6 months, have you attended any **events or activities** hosted for mothers and other caregivers of infants and young children, such as infant fairs/ festivals?	Yes ....1<br>No ....0<br>Don't know/don't remember 98	Media9

5. BREASTMILK SUBSTITUTE PROMOTION
(Questions are multiple-choice)

No	Question	Code	Variable name
1	In the past 30 days, did you receive **<read choice one by one>** of Milk for pregnant women?	Advice or recommendation ....1<br>Free sample ....2<br>Coupons ....3<br>Gift..4<br>Did not receive 5	Assess1<br><br><br><br>Chọn 5-> Q4
2	**From whom** did you receive <list what indicated in questions 1>?	Health workers in public health facilities......... 1<br>Health workers in private health facilities........ 2<br>Village health workers, nutrition / population collaborators, women union representatives .... 3<br>Partner/relative/friend .... 4<br>Shop/pharmacy personnel .... 5<br>Representative of a BMS company .... 6<br>Can't remember .... 98<br>Other (Specify) 77	Alwho1
3	**Where did** you receive <list what indicated in questions 1-4 >**?**	Public health facilities .... 1<br>Private health facilities .... 2<br>Home .... 3<br>Shops/ Pharmacies... 4<br>Onsite events, conferences .... 5<br><br>Television, Radio, Magazine, newspapers........ 6<br>Poster, Billboard.. 7<br><br>Social media (e.g. Facebook, Instagram, mobile chat apps) .... 8<br>Internet (e.g., online shops of a BMS company or food company, and of health workers) .... 9<br>Online events, conferences .... 10<br><br>Can't remember... 98<br>Other (Specify) 77	Alwhere1
4	In the past 30 days, did you receive **<read choice one by one>** of Formula milk for infants and toddlers (0+ months)?	Advice or recommendation ....1<br>Free sample ....2<br>Coupons ....3<br>Gift..4<br>Did not receive 5	Assess2<br><br><br>Chọn 5-> Q7
5	**From whom** did you receive <list what indicated in questions 1>?	Health workers in public health facilities......... 1<br>Health workers in private health facilities........ 2	Alwho2

		Village health workers, nutrition / population collaborators, women union representatives .... 3<br>Partner/relative/friend .... 4<br>Shop/pharmacy personnel .... 5<br>Representative of a BMS company .... 6<br>Can't remember... 98<br>Other (Specify)..77	
6	**Where did** you receive <list what indicated in questions 1-4 >**?**	Public health facilities .... 1<br>Private health facilities .... 2<br>Home .... 3<br>Shops/ Pharmacies.. 4<br>Onsite events, conferences .... 5<br><br>Television, Radio, Magazine, newspapers........ 6<br>Poster, Billboard... 7<br><br>Social media (e.g. Facebook, Instagram, mobile chat apps) .... 8<br>Internet (e.g., online shops of a BMS company or food company, and of health workers) .... 9<br>Online events, conferences .... 10<br><br>Can't remember.. 98<br>Other (Specify)..77	Alwhere2
7	In the past 30 days, did you receive **<read choice one by one>** of Bottle and artificial teats?	Advice or recommendation...........................1<br>Free sample ....2<br>Coupons ....3<br>Gift..4<br>Did not receive 5	Assess4<br><br><br><br>Chọn 5-> Part 6
8	**From whom** did you receive <list what indicated in questions 1>?	Health workers in public health facilities......... 1<br>Health workers in private health facilities........ 2<br>Village health workers, nutrition / population collaborators, women union representatives .... 3<br>Partner/relative/friend .... 4<br>Shop/pharmacy personnel .... 5<br>Representative of a BMS company .... 6<br>Can't remember... 98<br>Other (Specify)..77	Alwho4
9	**Where did** you receive <list what indicated in questions 1-4 >**?**	Public health facilities .... 1<br>Private health facilities .... 2<br>Home .... 3<br>Shops/ Pharmacies.. 4<br>Onsite events, conferences .... 5<br><br>Television, Radio, Magazine, newspapers........ 6<br>Poster, Billboard... 7<br><br>Social media (e.g. Facebook, Instagram, mobile chat apps) .... 8<br>Internet (e.g., online shops of a BMS company or food company, and of health workers) .... 9<br>Online events, conferences .... 10<br><br>Can't remember.. 98<br>Other (Specify)..77	Alwhere4

6. MATERNITY PROTECTION (ONLY ASK FOR EMPLOYED WOMEN)

(Only for women with a child who were in paid or unpaid work before birth, namely codes 2, 3 in mother occupation Q 18-section 1)

Now, I would like to ask about maternity leave benefits that you received or will receive

No	Question	Answer		Variable name
1	For this pregnancy, did / does your work involve **<read choice one by one>**?<br><br>Please use the following scale:<br>1 = Never/ Not<br>2 = Rarely<br>3 = Sometimes<br>4 = Often<br>5 = Always<br>98 = Don't know<br>99 No response	1. Manual lifting, carrying, pushing or pulling of loads…………………………….. _____<br>2. Exposure to biological, chemical or physical agents……………………………. _____<br>3. Prolonged periods of sitting or standing _____<br>4. Exposure to extreme temperatures or to vibration…………………………. _____<br>5. Night work (working for at least 2 hours between 22.00 and 05.00)…………… _____<br>6. Require plus working time.................. _____<br>7. Other hazards/unhealthy conditions (specify) .... _____<br>8. Business trip away from home when pregnant over 7 months……………………….. _____		expose
2	For this pregnancy, did you request lighter duties or to be transferred to a safer job?	1. Yes, and my request was accepted<br>2. Yes, but my request was not accepted<br>3. No, I did not request lighter duties/safer job even I wanted to<br>4. No, I did not request because my current job is safe and suitable		request
3	Do you think during this pregnancy, you were treated unfairly at work in any of the following ways?<br>*I will read each option and you choose what situation you fell into?*	1. I was given unsuitable work or workloads	1 Yes<br>0 No	fair
		2. I was moved to a less favorable position in terms of tasks and responsibilities (less senior position)	1 Yes<br>0 No	
		3. I had a reduction in my salary or bonus	1 Yes<br>0 No	
		4. I received a pay rise or bonus that was less than my peers at work	1 Yes<br>0 No	
		5. I received unpleasant comments from my employer and/or colleagues	1 Yes<br>0 No	
		6. I was unfairly criticized or disciplined about my performance at work	1 Yes<br>0 No	
		7. I failed to gain a promotion I felt I deserved or was otherwise sidelined	1 Yes<br>0 No	

		8. I was denied access to training that I would otherwise have received	1 Yes<br>0 No	
		9. I was treated so poorly that I felt I had to leave	1 Yes<br>0 No	
		10. I was dismissed	1 Yes<br>0 No	
		77. Other (specify)__ ___________________	1 Yes<br>0 No	

4	Will you get either paid or unpaid maternity leave for the coming child?	1. Yes<br>0. No<br>98. Don't know	Momoff
7	For the coming maternity leave, how many **days** do you plan to take?<br><br>*(convert answer to days if given in weeks or months)*	_ _ days	planoff
9b	Will you receive any cash benefits during the coming maternity leave?	1. Yes<br>0. No<br>98. Don't know	benyesb
13	Will your husband / partner get paternity leave when you give birth to the coming child?<br>(Ask this question if code of the husband's occupation are 2,3)	1. Yes<br>0. No<br>98. Don't know	dadoff
19	Which factors relating to the workplace have enable a woman in your company to continue breastfeeding her child?<br><br>(***multiple responses possible,*** tick according to mother's words)	1. Mothers can bring the child to work<br>2. Mothers live/lived close to workplace<br>3. Mothers can work/ worked from home<br>4. Mothers can take one or more daily breaks for breastfeeding/milk expression at the workplace<br>5. Mothers can enjoy a reduction in daily working hours to breastfeed at home<br>6. The employer provides / provided a lactation room<br>7. The employer provides / provided daycare or subsidizes the cost for daycare for employees	support

		8. There is a childcare facility close to the workplace<br><br>9. None of the above support factors. Mothers have to try themselves.<br><br>77. Other (specify)<br><br>_________________________	
20	According to the Vietnam Labor Code, what are, what are the rights of a working woman who is pregnant or nursing a child under 12 months?<br><br>(Multiple choice, don't read the option, can tick when mentioned a part of the answer)	1. Not to work at night, work overtime, or go on a long-distance travel if 7 months pregnant or more or nursing a child under 12 months.<br>2. No disciplining or terminating the job of a woman during her pregnancy or while nursing a child < 12 months.<br>3. Pregnant women can unilaterally terminate the employment contract, or to temporarily suspend the employment contract during pregnancy with certificate from a competent health care institution that continue working may adversely affect the pregnancy.<br>4. Paid leave during working hour for antenatal care.<br>5. Antenatal care fee covered by health insurance.<br>6. Paid leave for abortion, still birth, family planning, nursing a child < 6 months, caring for sick children < 7 years.<br>7. Paid maternity leave of 6 months (before plus after birth); and extended by one month for each additional child in multiple birth.<br>8. Negotiation about additional unpaid leave with the employer after completion of 6 months paid maternity leave.<br>9. One paid hour for nursing break (rest, express milk, going home to feed the child…)<br>10. Return to the same or at least similar position after maternity leave.<br>11. Received both salary from employer and social insurance if return at 5-6 months (with approval from doctor and mutual agreement with the employer).<br>98. Don't know / did not mention any<br><br>99. No response	aware

21. Maternity protection policy in Vietnam allows employed women 6 months paid maternity leave and 60-minute breastfeeding breaks a day in the first year after birth.

According to your opinion, what is the effect of of the policy on the following subjects (Variable name: begood**)**

1	2	3	4	5	6
**Very unprofitable**	**Unprofitable**	**Partly unprofitable**	**Partly profitable**	**Profitable**	**Very profitable**

	**Code**
0. Overall benefit for the society	
1. Employers	
2. Mother income	
3. Child health	
4. Mother health	
5. Mother career	
6. My preference on connecting with friends and coworkers	
7. The whole family	

7. Follow up questions

Occationnary, we need to contact respondent to clarify certain information provided or to ask about the interview experience and would need the contact information. The contact information will be used to reach the participant for further information only.

7.1 It is optional, but are you willing to share the best way to follow up with you?<br>0. No-> Thanks the women and stop<br>1. Yes-> fill in the mean of contact	OKcont
7.2 Reviewer self-evaluate if the woman would be a good candidate for IDI<br>0. No-> Thanks the women and stop<br>1. Yes	IDIellig
7.3 In the coming one or two days, we would like to meet you again to learn more about your experiences. It will last for about 45 minutes. Are you willing to participate in the meeting?<br>0. No-> Stop<br>1. Yes-> Read: a colleague of mine will contact you to set up the appropriate date, time, and location for the interview.	IDIagree

THANK YOU VERY MUCH FOR PARTICIPATING IN THIS SURVEY

Notes of the interviewer about the interview session (note_final)

1. Interview venue was quiet
2. No other adults were in the meeting
3. Minimal distraction of children

4. The woman was focus
5. The woman answered correctly
6. No issues relating to the questionnaire
7. No issues relating to the tablet
8. No other issues

Specify (when one of the 8 notes unchecked)

Getting the GEO code outdoor.
