# Supplementary material for: Assessing the Effectiveness of Policies Relating to Breastfeeding Promotion, Protection, and Support in Southeast Asia: Protocol for a Mixed Methods Study
Source: JMIR Res Protoc. 2020 Sep 21;9(9):e21286. doi: 10.2196/21286 (PMC7536596; doi:10.2196/21286)
Supplement: Multimedia Appendix 6 [file resprot_v9i9e21286_app6.pdf]

APPENDIX 2. INFORMED CONSENT FORMS

Oral informed consent: Policy makers' and authorities' IDI 2
Oral informed consent: Key Stakeholders' IDI 5
Oral informed consent: Employers' IDI 8
Oral informed consent: Health workers' IDI 11
Oral informed consent: Women's Survey Interview 14
Oral informed consent: Women's IDI 17
Oral informed consent: IDI for partners of the interviewed women or Father of children < 12 months 20

Oral informed consent: Policy makers' and authorities' IDI

Title: Assessing the effectiveness of policies relating to breastfeeding promotion, protection, and support in Southeast Asia.

Protocol Number: 1383644.

Sponsor: Bill & Melinda Gates Foundation.

Principal Investigator: Tuan Nguyen and Amy Weissman, Alive & Thrive Southeast Asia, FHI 360.

Address: R702, 7th Floor, Opera Business Center, 60 Ly Thai To Street, Hanoi, Viet Nam.

Site(s): Myanmar, Philippines, Thailand, Viet Nam (This is a multi-country research, so this will be specified in the country-specific informed consent).

Research Related Phone Numbers: *[specified phone number - would vary depending on the research firm and country]*.

Information about Taking Part in this Research
Good morning / afternoon. My name is *[state interviewer's name]*. I am working with *[institution's name, may include Alive & Thrive, FHI 360]*. You are being asked to take part in a research that will help us understand the implementation and benefit of policies relating to breastfeeding promotion, protection, and support in [Country Name] and in other countries in the region. This research will help to improve your country's policies and programs for mothers and children.

You were selected as a potential participant because you are a policy maker/authority who has played a role in developing, implementing, or monitoring polices related to [maternity protection OR the Code of Marketing of Breastmilk Substitutes]. Maternity protections are the benefits the government has put in place for families with young children. The Code of Marketing of Breastmilk Substitutes is an international regulation issued by World Health Assembly (WHA) to limit inappropriate marketing practices and the harmful effects of marketing of Breastmilk Substitutes, feeding bottles, and teats. About 15 other policy makers like you in [Country name] will also participate in this research.

Taking part in this research is voluntary. That means you do not have to participate. It also means that even if you say yes now, you can stop the interview at any time. If you choose to participate, the interviewer will ask you some questions about your experience developing, implementing, or monitoring policies related to [maternity protection OR the Code of Marketing of Breastmilk Substitutes]. We are also interested in learning about your perceptions of the current policy and any ways that the policy could be improved. The interview will take about 1 hour.

As we tell you more about this research, please ask us to explain any words or information that you may not understand. We want to be sure you can make the right decision for you about participating in this research.

Possible Risks
We anticipate only minimal risks associated with your participation in this research. Some questions may make you feel uncomfortable. If that happens, you can tell me that you do not want to answer that question. And at any time, you can tell me you would like to stop the interview. No one will know you've asked this and there will be no negative consequences if you do not participate in or stop the interview.

Possible Benefits
Your answers may not benefit you directly. However, they may help to improve your country's policies and programs for mothers and children.

Voluntary Participation
Being part of this research is voluntary. You are free to decide if you want to be in this research. You do not have to answer any questions you do not want to answer. If you agree to participate and then you change your mind, you are free to withdraw your consent and stop the interview at any time. It will not affect the benefits, medical care, or other services to which you are entitled.

Confidentiality
We will do our best to protect information about you and your participation in this research. All information you share will be kept confidential. It will be used only for the research purpose. We will assign each participant an ID that links your personal information and your answers. We will keep your personal information separately so that no one can link your answers with you. Any research information collected in paper form will be kept in a locked file cabinet in the FHI 360 office. It will remain there throughout the duration of the research and for three years after the end of the research and then it will be destroyed. Computer data will be password protected and only accessible by designated research staff.

We would also like to audio record the interview with a digital recorder. This will help us to remember all the information you share. Your name will not be recorded, and only the research team will listen to the audio recording. Once the data are used, the audio recording will also be permanently deleted. If you prefer not to be recorded, we will take notes of your responses instead.

Payment
After completing the interview, we would like to give you [name of the gift or amount of money]. This to thank you for your participation and to recognize the time you've given to this research.

If You Have a Questions About the Research
If you have any questions about the research contact Dr. Tuan Nguyen in Viet Nam at telephone +84-243-001 1544, email: tnguyen@fhi360.org or Dr. Amy Weissman at telephone + 66-2-263-5200 x20226, email: AWeissman@fhi360.org.

Your rights as a Participant
This research was reviewed and approved by the Institutional Review Board of FHI 360 [*and the local site*]. If you have any questions about how you are being treated by the research or your rights as a participant, you may contact *[specified name and contact info for local IRB].*

NOTE: *We will provide the local IRB information to FHI 360's PHSC once the contract with the local firm is signed and the local IRB identified.*

This research was also reviewed by FHI 360's Protection of Human Subjects Committee (PHSC) in the United States. You may contact the PHSC at FHI 360 at 359 Blackwell Street, Suite 200, Durham, NC 27701 or by phone number: [International Access Code]-1-919-405-1445, or by e-mail: PHSC@fhi360.org

STATEMENT OF CONSENT

I would like to remind you that participating in this research is your decision. You can tell me that you do not want to participate. Please take a moment to make your decision. *[Interviewer: Pause until the participant is ready.]*

Do you agree to take part in the interview (if you qualify)? [Interviewer: Check the box next to 'yes' or 'no' below, depending on what the person says.]
☐ Yes → *[Interviewer signs; the participant does not sign her/his name) to indicate that the participant has provided oral consent to take part in the interview.]*
☐ No → *[Interviewer: STOP, do not continue.]*

I certify that the nature and purpose, the potential benefits, and possible risks associated with participating in this research have been explained to the above individual.

____ __________________
Signature of Person Who Obtained Consent Date

[If a waiver of documentation of informed consent form is not approved, participants' signature will be required.]

Do you agree to having this conversation audio recorded? [Interviewer: Check the box next to 'yes' or 'no' below, depending on what the person says.]
☐ Yes → *[Interviewer: Proceed with recording.]*
☐ No → *[Interviewer: Take notes in place of audio recording.]*

Oral informed consent: Key Stakeholders' IDI

Title: Assessing the effectiveness of policies relating to breastfeeding promotion, protection, and support in Southeast Asia.

Protocol Number: 1383644.

Sponsor: Bill & Melinda Gates Foundation.

Principal Investigator: Tuan Nguyen and Amy Weissman, Alive & Thrive Southeast Asia, FHI 360.

Address: R702, 7th Floor, Opera Business Center, 60 Ly Thai To Street, Hanoi, Viet Nam.

Site(s): Myanmar, Philippines, Thailand, Viet Nam (This is a multi-country research, so this will be specified in the country-specific informed consent).

Research Related Phone Numbers: *[specified phone number - would vary depending on the research firm and country]*.

Information about Taking Part in this Research
Good morning / afternoon. My name is *[state interviewer's name]*. I am working with *[institution's name, may include Alive & Thrive, FHI 360]*. You are being asked to take part in a research that will help us understand the implementation and benefit of policies relating to breastfeeding promotion, protection, and support in [Country Name] and in other countries in the region. This research will help to improve your country's policies and programs for mothers and children.

You were selected as a potential participant because you are a key stakeholder who played a role in developing, implementing, or monitoring polices related to [maternity protection OR the Code of Marketing of Breastmilk Substitutes]. Maternity protections are the benefits the government has put in place for families with young children. The Code of Marketing of Breastmilk Substitutes is an international regulation issued by World Health Assembly (WHA) to limit inappropriate marketing practices and the harmful effects of marketing of Breastmilk Substitutes, feeding bottles, and teats. About 6 other keys stakeholders like you in [Country name] will also participate in this research.

Taking part in this research is voluntary. That means you do not have to participate. It also means that even if you say yes now, you can stop the interview at any time. If you choose to participate, the interviewer will ask you some questions about your experience developing, implementing, or monitoring policies related to [maternity protection OR the Code of Marketing of Breastmilk Substitutes]. We are also interested in learning about your perceptions of the current policy and any ways that the policy could be improved. The interview will take about 1 hour.

As we tell you more about this research, please ask us to explain any words or information that you may not understand. We want to be sure you can make the right decision for you about participating in this research.

Possible Risks

We anticipate only minimal risks associated with your participation in this research. Some questions may make you feel uncomfortable. If that happens, you can tell me that you do not want to answer that question. And at any time, you can tell me you would like to stop the interview. No one will know you've asked this and there will be no negative consequences if you do not participate in or stop the interview.

Possible Benefits

Your answers may not benefit you directly. However, they may help to improve your country's policies and programs for mothers and children.

Voluntary Participation

Being part of this research is voluntary. You are free to decide if you want to be in this research. You do not have to answer any questions you do not want to answer. If you agree to participate and then you change your mind, you are free to withdraw your consent and stop the interview at any time. It will not affect the benefits, medical care, or other services to which you are entitled.

Confidentiality

We will do our best to protect information about you and your participation in this research. All information you share will be kept confidential. It will be used only for the research purpose. We will assign each participant an ID that links your personal information and your answers. We will keep your personal information separately so that no one can link your answers with you. Any research information collected in paper form will be kept in a locked file cabinet in the FHI 360 office. It will remain there throughout the duration of the research and for three years after the end of the research and then it will be destroyed. Computer data will be password protected and only accessible by designated research staff.

We would also like to audio record the interview with a digital recorder. This will help us to remember all the information you share. Your name will not be recorded, and only the research team will listen to the audio recording. Once the data are used, the audio recording will also be permanently deleted. If you prefer not to be recorded, we will take notes of your responses instead.

Payment

After completing the interview, we would like to give you [name of the gift or amount of money]. This to thank you for your participation and to recognize the time you've given to this research.

If You Have a Questions About the Research

If you have any questions about the research contact Dr. Tuan Nguyen in Viet Nam at telephone +84-243-001 1544, email: tnguyen@fhi360.org or Dr. Amy Weissman at telephone + 66-2-263-5200 x20226, email: AWeissman@fhi360.org.

Your rights as a Participant

This research was reviewed and approved by the Institutional Review Board of FHI 360 [*and the local site*]. If you have any questions about how you are being treated by the research or your rights as a participant, you may contact *[specified name and contact info for local IRB].*

NOTE: *We will provide the local IRB information to FHI 360's PHSC once the contract with the local firm is signed and the local IRB identified.*

This research was also reviewed by FHI 360's Protection of Human Subjects Committee (PHSC) in the United States. You may contact the PHSC at FHI 360 at 359 Blackwell Street, Suite 200, Durham, NC 27701 or by phone number: [International Access Code]-1-919-405-1445, or by e-mail: PHSC@fhi360.org

STATEMENT OF CONSENT

I would like to remind you that participating in this research is your decision. You can tell me that you do not want to participate. Please take a moment to make your decision. *[Interviewer: Pause until the participant is ready.]*

Do you agree to take part in the interview (if you qualify)? [Interviewer: Check the box next to 'yes' or 'no' below, depending on what the person says.]
☐ Yes → *[Interviewer signs; the participant does not sign her/his name) to indicate that the participant has provided oral consent to take part in the interview.]*
☐ No → *[Interviewer: STOP, do not continue.]*

I certify that the nature and purpose, the potential benefits, and possible risks associated with participating in this research have been explained to the above individual.

____ __________________
Signature of Person Who Obtained Consent Date

[If a waiver of documentation of informed consent form is not approved, participants' signature will be required.]

Do you agree to having this conversation audio recorded? [Interviewer: Check the box next to 'yes' or 'no' below, depending on what the person says.]
☐ Yes → *[Interviewer: Proceed with recording.]*
☐ No → *[Interviewer: Take notes in place of audio recording.]*

Oral informed consent: Employers' IDI

Title: Assessing the effectiveness of policies relating to breastfeeding promotion, protection, and support in Southeast Asia.

Protocol Number: 1383644.

Sponsor: Bill & Melinda Gates Foundation.

Principal Investigator: Tuan Nguyen and Amy Weissman, Alive & Thrive Southeast Asia, FHI 360.

Address: R702, 7th Floor, Opera Business Center, 60 Ly Thai To Street, Hanoi, Viet Nam.

Site(s): Myanmar, Philippines, Thailand, Viet Nam (This is a multi-country research, so this will be specified in the country-specific informed consent).

Research Related Phone Numbers: *[specified phone number - would vary depending on the research firm and country]*.

Information about Taking Part in this Research

Good morning / afternoon. My name is *[state interviewer's name]*. I am working with *[institution's name, may include Alive & Thrive, FHI 360]*. You are being asked to take part in a research that will help us understand the implementation and benefit of policies relating to breastfeeding promotion, protection, and support in [Country Name] and in other countries in the region. This research will help to improve your country's policies and programs for mothers and children.

You were selected as a potential participant because you a representative of a business that employs fifty or more women, and maternity protection would be relevant to you. As you know, Maternity protections are the benefits the government has put in place for families with young children. About 12 representatives from businesses like yours in [Country name] will also participate in this research.

Taking part in this research is voluntary. That means you do not have to participate. It also means that even if you say yes now, you can stop the interview at any time. If you choose to participate, the interviewer will ask you some questions about your experience in implementing and monitoring maternity protection policies within your business. I will also ask your opinion on how to improve this policy. The interview will take about 1 hour.

As we tell you more about this research, please ask us to explain any words or information that you may not understand. We want to be sure you can make the right decision for you about participating in this research.

Possible Risks

We anticipate only minimal risks associated with your participation in this research. Some questions may make you feel uncomfortable. If that happens, you can tell me that you do not want to answer that question. And at any time, you can tell me you would like to stop the interview. No one will know you've asked this and there will be no negative consequences if you do not participate in or stop the interview.

Possible Benefits

Your answers may not benefit you directly. However, they may help to improve your country's policies and programs for mothers and children.

Voluntary Participation

Being part of this research is voluntary. You are free to decide if you want to be in this research. You do not have to answer any questions you do not want to answer. If you agree to participate and then you change your mind, you are free to withdraw your consent and stop the interview at any time. It will not affect the benefits, medical care, or other services to which you are entitled.

Confidentiality

We will do our best to protect information about you and your participation in this research. All information you share will be kept confidential. It will be used only for the research purpose. We will assign each participant an ID that links your personal information and your answers. We will keep your personal information separately so that no one can link your answers with you. Any research information collected in paper form will be kept in a locked file cabinet in the FHI 360 office. It will remain there throughout the duration of the research and for three years after the end of the research and then it will be destroyed. Computer data will be password protected and only accessible by designated research staff.

We would also like to audio record the interview with a digital recorder. This will help us to remember all the information you share. Your name will not be recorded, and only the research team will listen to the audio recording. Once the data are used, the audio recording will also be permanently deleted. If you prefer not to be recorded, we will take notes of your responses instead.

Payment

After completing the interview, we would like to give you [name of the gift or amount of money]. This to thank you for your participation and to recognize the time you've given to this research.

If You Have a Questions About the Research

If you have any questions about the research contact Dr. Tuan Nguyen in Viet Nam at telephone +84-243-001 1544, email: tnguyen@fhi360.org or Dr. Amy Weissman at telephone + 66-2-263-5200 x20226, email: AWeissman@fhi360.org.

Your rights as a Participant

This research was reviewed and approved by the Institutional Review Board of FHI 360 [*and the local site*]. If you have any questions about how you are being treated by the research or your rights as a participant, you may contact *[specified name and contact info for local IRB].*

NOTE: *We will provide the local IRB information to FHI 360's PHSC once the contract with the local firm is signed and the local IRB identified.*

This research was also reviewed by FHI 360's Protection of Human Subjects Committee (PHSC) in the United States. You may contact the PHSC at FHI 360 at 359 Blackwell Street, Suite 200, Durham, NC 27701 or by phone number: [International Access Code]-1-919-405-1445, or by e-mail: PHSC@fhi360.org

STATEMENT OF CONSENT

I would like to remind you that participating in this research is your decision. You can tell me that you do not want to participate. Please take a moment to make your decision. *[Interviewer: Pause until the participant is ready.]*

Do you agree to take part in the interview (if you qualify)? [Interviewer: Check the box next to 'yes' or 'no' below, depending on what the person says.]
☐ Yes → *[Interviewer signs; the participant does not sign her/his name) to indicate that the participant has provided oral consent to take part in the interview.]*
☐ No → *[Interviewer: STOP, do not continue.]*

I certify that the nature and purpose, the potential benefits, and possible risks associated with participating in this research have been explained to the above individual.

____ ____________________
Signature of Person Who Obtained Consent Date

[If a waiver of documentation of informed consent form is not approved, participants' signature will be required.]

Do you agree to having this conversation audio recorded? [Interviewer: Check the box next to 'yes' or 'no' below, depending on what the person says.]
☐ Yes → *[Interviewer: Proceed with recording.]*
☐ No → *[Interviewer: Take notes in place of audio recording.]*

Oral informed consent: Health workers' IDI

Title: Assessing the effectiveness of policies relating to breastfeeding promotion, protection, and support in Southeast Asia.

Protocol Number: 1383644.

Sponsor: Bill & Melinda Gates Foundation.

Principal Investigator: Tuan Nguyen and Amy Weissman, Alive & Thrive Southeast Asia, FHI 360.

Address: R702, 7th Floor, Opera Business Center, 60 Ly Thai To Street, Hanoi, Viet Nam.

Site(s): Myanmar, Philippines, Thailand, Viet Nam (This is a multi-country research, so this will be specified in the country-specific informed consent).

Research Related Phone Numbers: *[specified phone number - would vary depending on the research firm and country]*.

Information about Taking Part in this Research
Good morning / afternoon. My name is *[state interviewer's name]*. I am working with *[institution's name, may include Alive & Thrive, FHI 360]*. You are being asked to take part in a research that will help us understand the implementation and benefit of policies relating to breastfeeding promotion, protection, and support in [Country Name] and in other countries in the region. This research will help to improve your country's policies and programs for mothers and children.

You were selected as a potential participant because you are healthcare professional responsible for providing maternal and child health services, and the Code of Marketing of Breastmilk Substitutes would be relevant to you. As you know, the Code of Marketing of Breastmilk Substitutes is an international regulation issued by World Health Assembly (WHA) to limit inappropriate marketing practices and the harmful effects of marketing of breast milk substitutes, feeding bottles, and teats. About 12 health workers like you like you in [Country name] will also participate in this research.

Taking part in this research is voluntary. That means you do not have to participate. It also means that even if you say yes now, you can stop the interview at any time. If you choose to participate, the interviewer will ask you some questions about your experience implementing and monitoring of breastmilk substitutes at health facilities. The interview will take about 1 hour.

As we tell you more about this research, please ask us to explain any words or information that you may not understand. We want to be sure you can make the right decision for you about participating in this research.

Possible Risks
We anticipate only minimal risks associated with your participation in this research. Some questions may make you feel uncomfortable. If that happens, you can tell me that you do not want to answer that question. And at any time, you can tell me you would like to stop the interview. No one will know you've asked this and there will be no negative consequences if you do not participate in or stop the interview.

Possible Benefits
Your answers may not benefit you directly. However, they may help to improve your country's policies and programs for mothers and children.

Voluntary Participation
Being part of this research is voluntary. You are free to decide if you want to be in this research. You do not have to answer any questions you do not want to answer. If you agree to participate and then you change your mind, you are free to withdraw your consent and stop the interview at any time. It will not affect the benefits, medical care, or other services to which you are entitled.

Confidentiality
We will do our best to protect information about you and your participation in this research. All information you share will be kept confidential. It will be used only for the research purpose. We will assign each participant an ID that links your personal information and your answers. We will keep your personal information separately so that no one can link your answers with you. Any research information collected in paper form will be kept in a locked file cabinet in the FHI 360 office. It will remain there throughout the duration of the research and for three years after the end of the research and then it will be destroyed. Computer data will be password protected and only accessible by designated research staff.

We would also like to audio record the interview with a digital recorder. This will help us to remember all the information you share. Your name will not be recorded, and only the research team will listen to the audio recording. Once the data are used, the audio recording will also be permanently deleted. If you prefer not to be recorded, we will take notes of your responses instead.

Payment
After completing the interview, we would like to give you [name of the gift or amount of money]. This to thank you for your participation and to recognize the time you've given to this research.

If You Have a Questions About the Research
If you have any questions about the research contact Dr. Tuan Nguyen in Viet Nam at telephone +84-243-001 1544, email: tnguyen@fhi360.org or Dr. Amy Weissman at telephone + 66-2-263-5200 x20226, email: AWeissman@fhi360.org.

Your rights as a Participant
This research was reviewed and approved by the Institutional Review Board of FHI 360 [*and the local site*]. If you have any questions about how you are being treated by the research or your rights as a participant, you may contact *[specified name and contact info for local IRB].*

NOTE: *We will provide the local IRB information to FHI 360's PHSC once the contract with the local firm is signed and the local IRB identified.*

This research was also reviewed by FHI 360's Protection of Human Subjects Committee (PHSC) in the United States. You may contact the PHSC at FHI 360 at 359 Blackwell Street, Suite 200, Durham, NC 27701 or by phone number: [International Access Code]-1-919-405-1445, or by e-mail: PHSC@fhi360.org

STATEMENT OF CONSENT

I would like to remind you that participating in this research is your decision. You can tell me that you do not want to participate. Please take a moment to make your decision. *[Interviewer: Pause until the participant is ready.]*

Do you agree to take part in the interview (if you qualify)? [Interviewer: Check the box next to 'yes' or 'no' below, depending on what the person says.]
☐ Yes → *[Interviewer signs; the participant does not sign her/his name) to indicate that the participant has provided oral consent to take part in the interview.]*
☐ No → *[Interviewer: STOP, do not continue.]*

I certify that the nature and purpose, the potential benefits, and possible risks associated with participating in this research have been explained to the above individual.

____ __________________
Signature of Person Who Obtained Consent Date

[If a waiver of documentation of informed consent form is not approved, participants' signature will be required.]

Do you agree to having this conversation audio recorded? [Interviewer: Check the box next to 'yes' or 'no' below, depending on what the person says.]
☐ Yes → *[Interviewer: Proceed with recording.]*
☐ No → *[Interviewer: Take notes in place of audio recording.]*

Oral informed consent: Women's Survey Interview

Title: Assessing the effectiveness of policies relating to breastfeeding promotion, protection, and support in Southeast Asia.

Protocol Number: 1383644.

Sponsor: Bill & Melinda Gates Foundation.

Principal Investigator: Tuan Nguyen and Amy Weissman, Alive & Thrive Southeast Asia, FHI 360.

Address: R702, 7th Floor, Opera Business Center, 60 Ly Thai To Street, Hanoi, Viet Nam.

Site(s): Myanmar, Philippines, Thailand, Viet Nam (This is a multi-country research, so this will be specified in the country-specific informed consent).

Research Related Phone Numbers: *[specified phone number - would vary depending on the research firm and country]*.

Information about Taking Part in this Research
Good morning / afternoon. My name is *[state interviewer's name]*. I am working with *[institution's name, may include Alive & Thrive, FHI 360]*. You are being asked to take part in a research that will help us understand the implementation and benefit of policies relating to breastfeeding promotion, protection, and support in [Country Name] and in other countries in the region. This research will help to improve your country's policies and programs for mothers and children.

You were selected as a potential participant because you are a [pregnant woman and/ or a mother of a child under 12 months of age]. About 1000 women like you in [Country name] will also participate in this research.

Taking part in this research is voluntary. That means you do not have to participate. It also means that even if you say yes now, you can stop the interview at any time. If you choose to participate, the interviewer will ask you some questions about your knowledge, belief and practices on child health and nutrition and related factors. The interview will take about 1 hour.

As we tell you more about this research, please ask us to explain any words or information that you may not understand. We want to be sure you can make the right decision for you about participating in this research.

Possible Risks
We anticipate only minimal risks associated with your participation in this research. Some questions may make you feel uncomfortable. If that happens, you can tell me that you do not want to answer that question. And at any time, you can tell me you would like to stop the

interview. No one will know you've asked this and there will be no negative consequences if you do not participate in or stop the interview.

Possible Benefits
Your answers may not benefit you directly. However, they may help to improve your country's policies and programs for mothers and children.

Voluntary Participation
Being part of this research is voluntary. You are free to decide if you want to be in this research. You do not have to answer any questions you do not want to answer. If you agree to participate and then you change your mind, you are free to withdraw your consent and stop the interview at any time. It will not affect the benefits, medical care, or other services to which you are entitled.

Confidentiality
We will do our best to protect information about you and your participation in this research. All information you share will be kept confidential. It will be used only for the research purpose. We will assign each participant an ID that links your personal information and your answers. We will keep your personal information separately so that no one can link your answers with you. Any research information collected in paper form will be kept in a locked file cabinet in the FHI 360 office. It will remain there throughout the duration of the research and for three years after the end of the research and then it will be destroyed. Computer data will be password protected and only accessible by designated research staff.

Payment
After completing the interview, we would like to give you [name of the gift or amount of money]. This to thank you for your participation and to recognize the time you've given to this research.

If You Have a Questions About the Research
If you have any questions about the research contact Dr. Tuan Nguyen in Viet Nam at telephone +84-243-001 1544, email: tnguyen@fhi360.org or Dr. Amy Weissman at telephone + 66-2-263-5200 x20226, email: AWeissman@fhi360.org.

Your rights as a Participant
This research was reviewed and approved by the Institutional Review Board of FHI 360 [*and the local site*]. If you have any questions about how you are being treated by the research or your rights as a participant, you may contact *[specified name and contact info for local IRB].*

NOTE: *We will provide the local IRB information to FHI 360's PHSC once the contract with the local firm is signed and the local IRB identified.*

This research was also reviewed by FHI 360's Protection of Human Subjects Committee (PHSC) in the United States. You may contact the PHSC at FHI 360 at 359 Blackwell Street, Suite 200, Durham, NC 27701 or by phone number: [International Access Code]-1-919-405-1445, or by e-mail: PHSC@fhi360.org

STATEMENT OF CONSENT

I would like to remind you that participating in this research is your decision. You can tell me that you do not want to participate. Please take a moment to make your decision. *[Interviewer: Pause until the participant is ready.]*

Do you agree to take part in the interview (if you qualify)? [Interviewer: Check the box next to 'yes' or 'no' below, depending on what the person says.]
☐ Yes → *[Interviewer signs; the participant does not sign her/his name) to indicate that the participant has provided oral consent to take part in the interview.]*
☐ No → *[Interviewer: STOP, do not continue.]*

I certify that the nature and purpose, the potential benefits, and possible risks associated with participating in this research have been explained to the above individual.

____ ___________________

Signature of Person Who Obtained Consent Date

[If a waiver of documentation of informed consent form is not approved, participants' signature will be required.]

Oral informed consent: Women's IDI

Title: Assessing the effectiveness of policies relating to breastfeeding promotion, protection, and support in Southeast Asia.

Protocol Number: 1383644.

Sponsor: Bill & Melinda Gates Foundation.

Principal Investigator: Tuan Nguyen and Amy Weissman, Alive & Thrive Southeast Asia, FHI 360.

Address: R702, 7th Floor, Opera Business Center, 60 Ly Thai To Street, Hanoi, Viet Nam.

Site(s): Myanmar, Philippines, Thailand, Viet Nam (This is a multi-country research, so this will be specified in the country-specific informed consent).

Research Related Phone Numbers: *[specified phone number - would vary depending on the research firm and country]*.

Information about Taking Part in this Research

Thank you for already participating in the survey. Of the 1000 women participated in the survey in [Country Name], we will ask about 36 women more in-depth questions. Given your experience we'd like to ask some more in-depth questions about reasons behind your practices. The interview will take about 1 hour. This study will help to improve your country's policies and programs for mothers and children.

Like with the survey, taking part in this research study is voluntary. That means you do not have to participate. It also means that even if you say yes now, you can stop the interview at any time.

As we tell you more about this research study, please ask us to explain any words or information that you may not understand. We want to be sure you can make the right decision for you about participating.

Possible Risks

We anticipate only minimal risks associated with your participation in this research. Some questions may make you feel uncomfortable. If that happens, you can tell me that you do not want to answer that question. And at any time, you can tell me you would like to stop the interview. No one will know you've asked this and there will be no negative consequences if you do not participate in or stop the interview.

Possible Benefits

Your answers may not benefit you directly. However, they may help to improve your country's policies and programs for mothers and children.

Voluntary Participation

Being part of this research is voluntary. You are free to decide if you want to be in this research. You do not have to answer any questions you do not want to answer. If you agree to participate and then you change your mind, you are free to withdraw your consent and stop the interview at any time. It will not affect the benefits, medical care, or other services to which you are entitled.

Confidentiality
We will do our best to protect information about you and your participation in this research. All information you share will be kept confidential. It will be used only for the research purpose. We will assign each participant an ID that links your personal information and your answers. We will keep your personal information separately so that no one can link your answers with you. Any research information collected in paper form will be kept in a locked file cabinet in the FHI 360 office. It will remain there throughout the duration of the research and for three years after the end of the research and then it will be destroyed. Computer data will be password protected and only accessible by designated research staff.

We would also like to audio record the interview with a digital recorder. This will help us to remember all the information you share. Your name will not be recorded, and only the research team will listen to the audio recording. Once the data are used, the audio recording will also be permanently deleted. If you prefer not to be recorded, we will take notes of your responses instead.

Payment
After completing the interview, we would like to give you [name of the gift or amount of money]. This to thank you for your participation and to recognize the time you've given to this research.

If You Have a Questions About the Research
If you have any questions about the research contact Dr. Tuan Nguyen in Viet Nam at telephone +84-243-001 1544, email: tnguyen@fhi360.org or Dr. Amy Weissman at telephone + 66-2-263-5200 x20226, email: AWeissman@fhi360.org.

Your rights as a Participant
This research was reviewed and approved by the Institutional Review Board of FHI 360 [*and the local site*]. If you have any questions about how you are being treated by the research or your rights as a participant, you may contact *[specified name and contact info for local IRB].*

NOTE: *We will provide the local IRB information to FHI 360's PHSC once the contract with the local firm is signed and the local IRB identified.*

This research was also reviewed by FHI 360's Protection of Human Subjects Committee (PHSC) in the United States. You may contact the PHSC at FHI 360 at 359 Blackwell Street, Suite 200, Durham, NC 27701 or by phone number: [International Access Code]-1-919-405-1445, or by e-mail: PHSC@fhi360.org

STATEMENT OF CONSENT

I would like to remind you that participating in this research is your decision. You can tell me that you do not want to participate. Please take a moment to make your decision. *[Interviewer: Pause until the participant is ready.]*

Do you agree to take part in the interview (if you qualify)? [Interviewer: Check the box next to 'yes' or 'no' below, depending on what the person says.]

☐ Yes → *[Interviewer signs; the participant does not sign her/his name) to indicate that the participant has provided oral consent to take part in the interview.]*

☐ No → *[Interviewer: STOP, do not continue.]*

I certify that the nature and purpose, the potential benefits, and possible risks associated with participating in this research have been explained to the above individual.

____ __________________

Signature of Person Who Obtained Consent Date

[If a waiver of documentation of informed consent form is not approved, participants' signature will be required.]

Do you agree to having this conversation audio recorded? [Interviewer: Check the box next to 'yes' or 'no' below, depending on what the person says.]

☐ Yes → *[Interviewer: Proceed with recording.]*

☐ No → *[Interviewer: Take notes in place of audio recording.]*

Oral informed consent: IDI for partners of the interviewed women or Father of children < 12 months

Title: Assessing the effectiveness of policies relating to breastfeeding promotion, protection, and support in Southeast Asia.

Protocol Number: 1383644.

Sponsor: Bill & Melinda Gates Foundation.

Principal Investigator: Tuan Nguyen and Amy Weissman, Alive & Thrive Southeast Asia, FHI 360.

Address: R702, 7th Floor, Opera Business Center, 60 Ly Thai To Street, Hanoi, Viet Nam.

Site(s): Myanmar, Philippines, Thailand, Viet Nam (This is a multi-country research, so this will be specified in the country-specific informed consent).

Research Related Phone Numbers: *[specified phone number - would vary depending on the research firm and country]*.

Information about Taking Part in this Research

Good morning / afternoon. My name is *[state interviewer's name]*. I am working with *[institution's name, may include Alive & Thrive, FHI 360]*. You are being asked to take part in a research that will help us understand the implementation and benefit of policies relating to breastfeeding promotion, protection, and support in [Country Name] and in other countries in the region. This research will help to improve your country's policies and programs for mothers and children.

You were selected as a potential participant because you are a father of a child under 12 months of age. About 12 men like you in [Country name] will also participate in this research.

Taking part in this research study is voluntary. That means you do not have to participate. It also means that even if you say yes now, you can stop the interview at any time. If you choose to participate, the interviewer will ask you some questions about your childcare and feeding arrangements. The interview will take about 1 hour.

As we tell you more about this research, please ask us to explain any words or information that you may not understand. We want to be sure you can make the right decision for you about participating in this research.

Possible Risks

We anticipate only minimal risks associated with your participation in this research. Some questions may make you feel uncomfortable. If that happens, you can tell me that you do not want to answer that question. And at any time, you can tell me you would like to stop the interview. No one will know you've asked this and there will be no negative consequences if you do not participate in or stop the interview.

Possible Benefits
Your answers may not benefit you directly. However, they may help to improve your country's policies and programs for mothers and children.

Voluntary Participation
Being part of this research is voluntary. You are free to decide if you want to be in this research. You do not have to answer any questions you do not want to answer. If you agree to participate and then you change your mind, you are free to withdraw your consent and stop the interview at any time. It will not affect the benefits, medical care, or other services to which you are entitled.

Confidentiality
We will do our best to protect information about you and your participation in this research. All information you share will be kept confidential. It will be used only for the research purpose. We will assign each participant an ID that links your personal information and your answers. We will keep your personal information separately so that no one can link your answers with you. Any research information collected in paper form will be kept in a locked file cabinet in the FHI 360 office. It will remain there throughout the duration of the research and for three years after the end of the research and then it will be destroyed. Computer data will be password protected and only accessible by designated research staff.

We would also like to audio record the interview with a digital recorder. This will help us to remember all the information you share. Your name will not be recorded, and only the research team will listen to the audio recording. Once the data are used, the audio recording will also be permanently deleted. If you prefer not to be recorded, we will take notes of your responses instead.

Payment
After completing the interview, we would like to give you [name of the gift or amount of money]. This to thank you for your participation and to recognize the time you've given to this research.

If You Have a Questions About the Research
If you have any questions about the research contact Dr. Tuan Nguyen in Viet Nam at telephone +84-243-001 1544, email: tnguyen@fhi360.org or Dr. Amy Weissman at telephone + 66-2-263-5200 x20226, email: AWeissman@fhi360.org.

Your rights as a Participant
This research was reviewed and approved by the Institutional Review Board of FHI 360 [*and the local site*]. If you have any questions about how you are being treated by the research or your rights as a participant, you may contact *[specified name and contact info for local IRB].*

NOTE: *We will provide the local IRB information to FHI 360's PHSC once the contract with the local firm is signed and the local IRB identified.*

This research was also reviewed by FHI 360's Protection of Human Subjects Committee (PHSC) in the United States. You may contact the PHSC at FHI 360 at 359 Blackwell Street, Suite 200, Durham, NC 27701 or by phone number: [International Access Code]-1-919-405-1445, or by e-mail: PHSC@fhi360.org

STATEMENT OF CONSENT

I would like to remind you that participating in this research is your decision. You can tell me that you do not want to participate. Please take a moment to make your decision. *[Interviewer: Pause until the participant is ready.]*

Do you agree to take part in the interview (if you qualify)? [Interviewer: Check the box next to 'yes' or 'no' below, depending on what the person says.]
☐ Yes → *[Interviewer signs; the participant does not sign her/his name) to indicate that the participant has provided oral consent to take part in the interview.]*
☐ No → *[Interviewer: STOP, do not continue.]*

I certify that the nature and purpose, the potential benefits, and possible risks associated with participating in this research have been explained to the above individual.

____ __________________
Signature of Person Who Obtained Consent Date

[If a waiver of documentation of informed consent form is not approved, participants' signature will be required.]

Do you agree to having this conversation audio recorded? [Interviewer: Check the box next to 'yes' or 'no' below, depending on what the person says.]
☐ Yes → *[Interviewer: Proceed with recording.]*
☐ No → *[Interviewer: Take notes in place of audio recording.]*
