# Supplementary material for: Assessing the Effectiveness of Policies Relating to Breastfeeding Promotion, Protection, and Support in Southeast Asia: Protocol for a Mixed Methods Study
Source: JMIR Res Protoc. 2020 Sep 21;9(9):e21286. doi: 10.2196/21286 (PMC7536596; doi:10.2196/21286)
Supplement: Multimedia Appendix 7 [file resprot_v9i9e21286_app7.pdf]

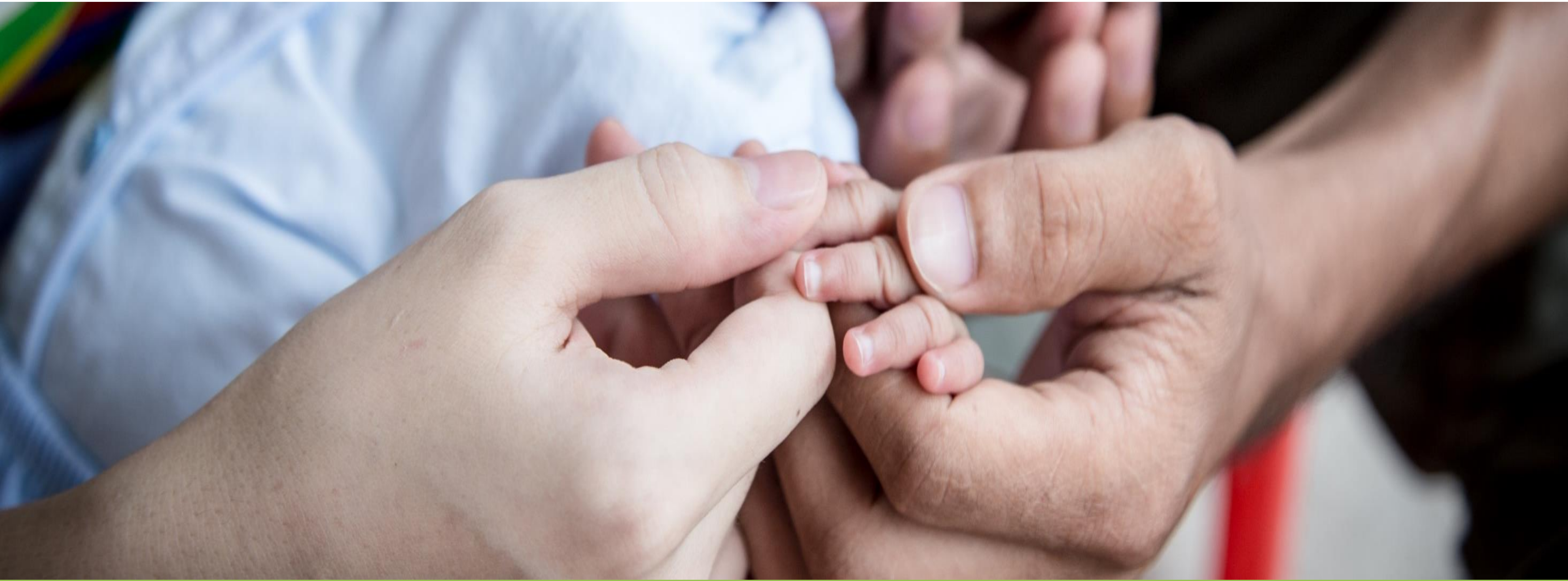

Assessing the effectiveness of policies relating to breastfeeding promotion, protection, and support in Southeast Asia

alive&thrive
nourish. nurture. grow.

Background

- Despite all that is known about the benefits of breastfeeding, breastfeeding practices remain sub-optimal worldwide.
- Various factors from individual to global levels would be associated with breastfeeding practices.
- Maternity protection legislation and the International Code of Marketing of Breast-Milk Substitutes (The Code) are key policies relating to maternity promotion, protection, and support.

Goal

To review the content, implementation, and potential impact of policies relating to breastfeeding promotion, protection, and support in Myanmar, the Philippines, and Viet Nam.

Aims / Objectives:

1. Review the content of national maternity protection and BMS Code policies;
2. Review the implementation, coverage, monitoring, and enforcement of these policies across countries;
3. Examine the potential impact of these policies on relevant outcomes (e.g., work force participation of women for maternity protection; and exposure to BMS marketing for BMS Code); and
4. Examine perceptions of relevant stakeholders and beneficiaries (e.g. pregnant and lactating women) about these policies (e.g. perceived benefit, limitations, difficulties, areas for improvement, recommendations).

Conceptual model

Policies and Contextual Factors

Structural
Population
Economics
Political regime
Sociocultural context
Labor and work force

Policies relating to the Code
Contents
Financing
Monitoring and enforcement

Maternity protection policies
Contents
Financing
Monitoring and enforcement

Facilitators and Barriers

	Facilitators, interventions	Barriers, challenges
**Mass media (Traditional or modern channels)**	Promotion of breastfeeding<br>Maternity protection<br>Reporting of violations	Marketing of BMS:<br>• Cross-promotion<br>• Coupons or discounts<br>• Advice
**Individual, family and community**	Counselling, education, and support<br>Family or social support<br>Supportive knowledge, beliefs, social norms, self-efficacy	Marketing of BMS:<br>• At points of sale<br>• Professional or peer advice<br>• Events, clubs
**Health system and services**	Baby-friendly environment<br>Breastfeeding counselling, education, and support<br>Early essential newborn care<br>Human milk bank	Marketing of BMS:<br>• Cross-promotion<br>• Professional or peer advice<br>• Free sample<br>• At points of sale<br>Cesarean birth<br>Mother child separation
**Work environment**	Maternal leave policy<br>Workplace support<br>Employment status	Discrimination against pregnant women and women with young children<br>Low awareness about the rights

Impacts

Recommended breastfeeding practices

Improved nutrition for women and children

Decreased maternal and child morbidity and mortality

Improved cognition, growth, and development

Increased work capacity and productivity

Socioeconomic development and increased equity

Study approaches for each study aim

Aims	Desk reviews	Trend data analysis	In-depth interview	Surveys
Aim 1. To review the content of national policies focusing on maternity protection and BMS Codes.	√			
Aim 2. To review implementation, coverage, monitoring, and enforcement of these policies across countries;	√		√	
Aim 3. To examine the potential impact of these policies on relevant outcomes		√		√
Aim 4. To examine perceptions of relevant stakeholders and beneficiaries about these policies			√	

List of key outcomes and indicators

	Maternity protection	BMS Codes
**Aim 1. Review policy content**		
Policy general information (Title; Published by; Year Published; Adopted by; Year Adopted; Start year; End year)	√	√
Policy legal status (Legal status; Legislation; Governing Resources; Categories)	√	√
Eligibility for the maternity protection<br>Maternity protection for employed women<br>Maternity protection for other groups<br>Related guidelines and regulations	√	
Product coverage<br>Duration of the coverage<br>Inclusion of acceptable reasons for using BMS<br>Related guidelines and regulations		√
**Aim 2. Review policy implementation strategies; coverage; monitoring and enforcement**		
Implementation strategies & Coverage<br>Monitoring; Enforcement; Modification Documentation	√	√

Sample size in each country

	In-depth interview (Up to)	Quantitative survey
Policy makers or authorities at national levels	7	
Stakeholders from UN, NGOs, research organizations, media	7	
Policy makers or authorities at sub-national levels (from up to 4 provinces)	10	
Employers	12	
Health workers	12	
Women:		
Pregnant	12	310
Mothers of children aged 0-11 months	24	620
Partners of the interviewed mothers with children 0-11 months (Sampling 1) or Fathers of children 0-11 months (Sampling 2)	12	
Total	96	930

Sampling procedure

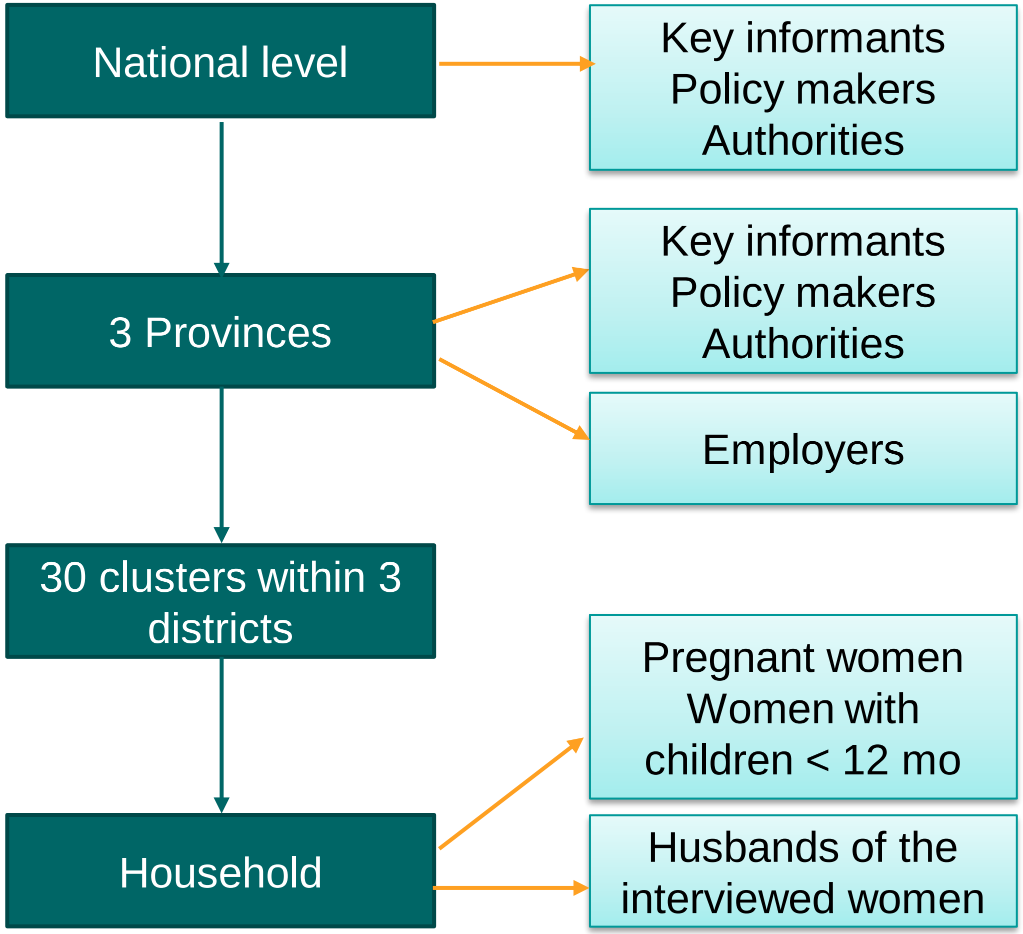

Sampling strategies for women and partners in each province

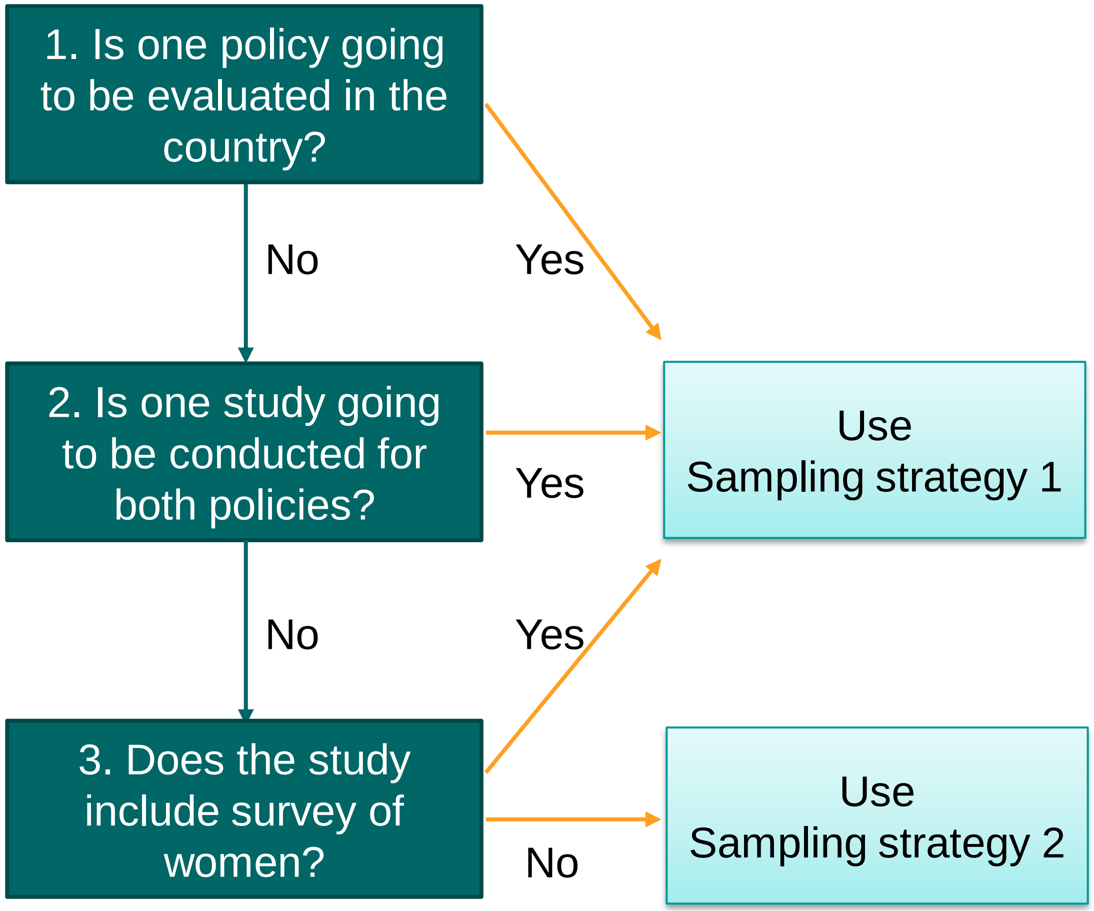

Domains of in-depth interviews by study participant

	Policy makers or authorities	Other stakeholders[1]	Employers or health workers	Women with child 0-11 months	Partners of the women
Policy(ies) development	√	√			
Policy(ies) implementation	√	√	√		
Policy(ies) monitoring and enforcement	√	√	√	√	√
Perceptions of and experience with policy(ies)	√	√	√	√	√
Suggestions for improvement	√	√	√	√	√
Sharing responsibilities caring for children and domestic tasks				√	√

List of key outcomes and indicators

	Maternity protection	BMS Codes
**Aim 3. Examine potential association between the policies on relevant outcomes**		
Quantitative survey of women and / or secondary data analysis:		
Women characteristics (Age; education; working status; contribution to social security fund)	√	√
Antenatal history (Content of antenatal care; Skilled attendant at delivery; Institutional deliveries; Caesarean section)	√	√
Child characteristics (age; gender)	√	√
Child feeding practices (Children ever breastfed; Early initiation of breastfeeding; Exclusive breastfeeding under 6 months; Feeding infant formula; Using bottle and artificial nipple)	√	√
Knowledge; beliefs; social norms and self-efficacy relating to infant feeding	√	√
Access to breastfeeding information (mass media; social media; interpersonal advice; events)	√	√
Exposure to BMS marketing via various channels (mass media; social media; interpersonal advice; events; free samples; coupons; gifts)		√
Exposure to information about maternity protection (mass media; social media; interpersonal advice; events; free samples; coupons; gifts)	√	
Duration of maternity leave eligible and actually take	√	
Maternity benefits (cash; medical)	√	
Work protection (health; employment and discrimination)	√	
Breastfeeding upon return to work	√	√
Childcare arrangements	√	

List of key outcomes and indicators by policy assessment

	Maternity protection	BMS Codes
**Aim 3. Examine potential association between the policies on relevant outcomes**		
Trend data:		
Time series data relating to maternity protection (Female labor force participation; Job retention; Payments and wages; Timing of return to work<br>The use of maternity leave; Use of nursing breaks and/or flexible schedules)	√	
Time series data relating to BMS codes (Advertising expenditure of BMS companies by product and channel; Sales of BMS by BMS type; Early initiation of breastfeeding; Exclusive breastfeeding)		√
**Aim 4. Explore perceptions of stakeholders and beneficiaries about the policies**		
In-depth interview: (policy development; implementation; monitoring; enforcement; usage; bottleneck; recommendation – See Table 4)	√	√

Domains of quantitative survey with women

Domains	Pregnant women	Mothers with children 0-11 months
Socio-demographic characteristics and working status	√	√
History antenatal care	√	√
Child characteristics		√
Child feeding practices		√
Social norms and self-efficacy relating to breastfeeding		√
Knowledge relating to breastfeeding	√	√
Intention relating to breastfeeding	√	
Access to breastfeeding information (mass media, social media, interpersonal advice, events)	√	√
Exposure to BMS marketing via various channels (mass media, social media, interpersonal advice, events, free samples, coupons, gifts)	√	√
Knowledge about maternity leave policies	√	√
Duration of maternity leave		√
Maternity benefits (cash, medical)		√
Work protection (health, employment and discrimination)		√
Breastfeeding upon return to work		√
Childcare arrangements		√

STAY CONNECTED WITH ALIVE & THRIVE

@aliveandthrive
facebook.com/fhi360.aliveandthrive
youtube.com/aliveandthrive

www.aliveandthrive.org

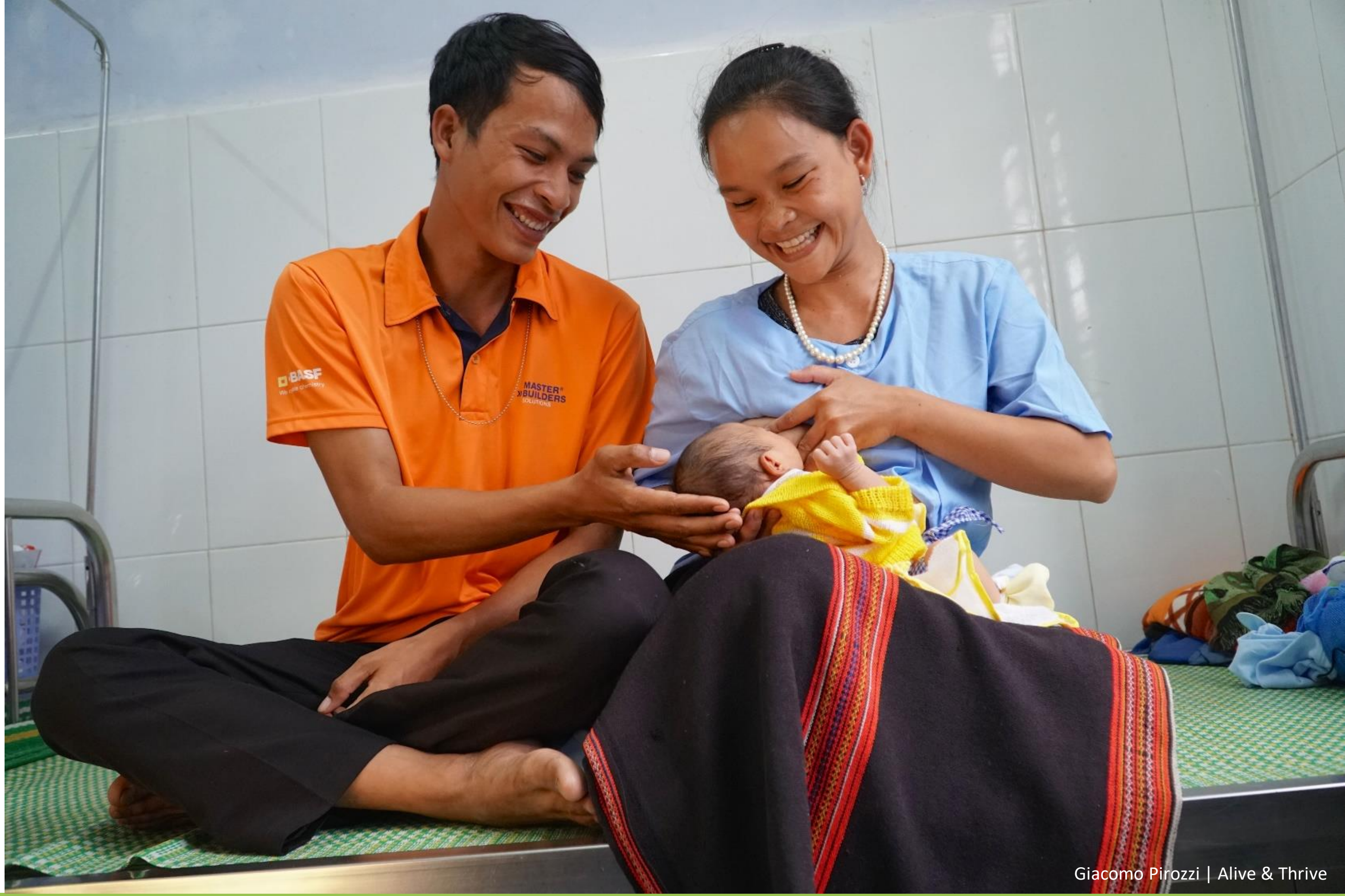

The Alive & Thrive initiative, managed by FHI 360, is currently funded by the Bill & Melinda Gates Foundation, Irish Aid, the Tanoto Foundation, and UNICEF.

alive&thrive
nourish. nurture. grow.
